# Synthesis and Biological Evaluation of Tetrahydroisoquinoline Derivatives as Trypanocidal Agents

**DOI:** 10.1021/acsomega.5c11033

**Published:** 2026-01-21

**Authors:** João Paulo de Moura Lopes, Gabriel Vitor de Lima Marques, Lucas Abreu Diniz, Viviane Côrrea Santos, Daniela de Melo Resende, Silvane Maria Fonseca Murta, Markus Kohlhoff, Vinícius Gonçalves Maltarollo, Rafaela Salgado Ferreira, Renata Barbosa Oliveira

**Affiliations:** † Departamento de Produtos Farmacêuticos, 28114 Universidade Federal de Minas Gerais, Belo Horizonte 31270901, Brazil; ‡ Departamento de Bioquímica e Imunologia, Universidade Federal de Minas Gerais, Belo Horizonte 31270-901, Brazil; § Instituto René Rachou—FIOCRUZ Minas, Belo Horizonte 30190002, Brazil; ∥ Department of Chemistry, Grand Valley State University, Allendale, Michigan 49401, United States

## Abstract

American trypanosomiasis is a parasitic illness of major public health relevance, resulting from infection with the protozoan *Trypanosoma cruzi* and predominantly impacting populations in low-resource settings. Current treatments, benznidazole and nifurtimox, are limited by their efficacy in the chronic phase, toxicity, and side effects, necessitating the search for new therapeutic agents. Cruzain, a key protease for parasite survival and infection, is a validated drug target. This work involved the synthesis and characterization of novel amides derived from 1,2,3,4-tetrahydroisoquinoline-3-carboxylic acid. Their activity was evaluated against both cruzain and *T. cruzi*. The hydrochloride salts **4a** and **4b** showed moderate cruzain inhibition (60.2 ± 2.4% and 69.3 ± 2.6% inhibition at 100 μM, respectively). Notably, compound **3d** and its hydrochloride salt **4d** demonstrated significant antiparasitic activity with IC_50_ values of 10.5 and 13.7 μM, respectively. However, their low cruzain inhibition (∼15%) suggests that their mechanism of action is likely through a different biological target.

## Graphical abstract

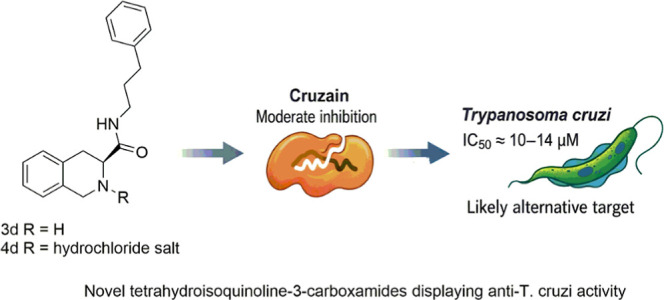

## 1 Introduction

Infection with the protozoan *Trypanosoma cruzi* gives rise to a persistent disorder that affects multiple organs and represents a major challenge in parasitic disease research. Transmission to humans occurs mainly through exposure to the feces of infected insects belonging to the Triatominae subfamily, popularly known as kissing bugs.^1^ The disease initially presents with an acute phase that lasts for approximately two months following infection and is marked by high levels of parasitemia, often accompanied by absent or nonspecific clinical symptoms, and a late chronic phase, in which the protozoan is found in its intracellular form, primarily in the muscle cells of the heart and gastrointestinal system. After a period of 10–30 years, about a third of infected individuals develop symptoms related to cardiac disorders and about a tenth develop digestive, neurological, or mixed changes. These changes are potentially fatal, primarily due to the destruction of the nervous system and heart muscle, resulting in arrhythmia and progressive heart failure.^2^

The World Health Organization has recognized Chagas disease since 2005 as 1 of the 20 conditions classified as neglected tropical diseases. It is estimated that approximately 6–7 million individuals worldwide are infected with *T. cruzi*, with the main affected regions being endemic areas in 21 Latin American countries.^2,3^ According to the drugs for neglected diseases initiative (DNDi), approximately 100 million people are at risk of infection, and an estimated 40,000 new cases occur annually, of which only 10% are diagnosed. Furthermore, Chagas disease is responsible for 10,000 deaths annually, making it the parasitic disease that causes the most deaths annually in Latin America.^4^

Currently, treatment of *T. cruzi* infection relies exclusively on the nitroheterocyclic drugs benznidazole (BZN) and nifurtimox (NFX), which are recommended for use in cases of acute infections (including newborns or infants infected through congenital transmission), reactivation during the chronic phase, chronic patients up to 18 years of age, and infected women of reproductive age. In addition to their use being limited to acute infections, other limitations of these drugs include prolonged treatment (60–90 days), contraindication during pregnancy due to genotoxic effects, and the occurrence of adverse events that result in significant treatment discontinuation rates.^5^ Therefore, the search for new treatment strategies/regimens is justified, as well as the search for new drugs capable of treating patients in both phases of the disease, in a shorter time frame, and providing a better safety profile.

Among the parasite’s molecular targets considered for the development of new anti-*T. cruzi* agents, the protease cruzain (also known as Cruzipain) is particularly noteworthy. It is a cysteine protease initially expressed as a zymogen in all stages of the parasite’s development and in this context plays a central role in multiple biological functions, including metabolic support, regulation of immune cell activity, and facilitation of entry into host tissues.^6^

Via a docking-based virtual screening, Santos et al.^7^ identified the commercially acquired racemic mixture of furfurylamide of *R* and *S*-1,2,3,4-tetrahydroisoquinolin-3-carboxylic acids (THIQA) as cruzain-competitive inhibitors. Thus, the present work aims to synthesize new derivatives of this acid and to evaluate their potential to inhibit cruzain as well as their trypanocidal activity.

## 2 Results and Discussion

To synthesize the intended THIQA amides, the first synthetic step consisted of protection of the amine group of the tetrahydroisoquinoline ring, in order to avoid cross side reactions during the respective amides’ formation. For this purpose, (Boc)_2_O was employed in alkaline medium, with THF as the solvent^8^ (Scheme 1).

**Scheme 1:**
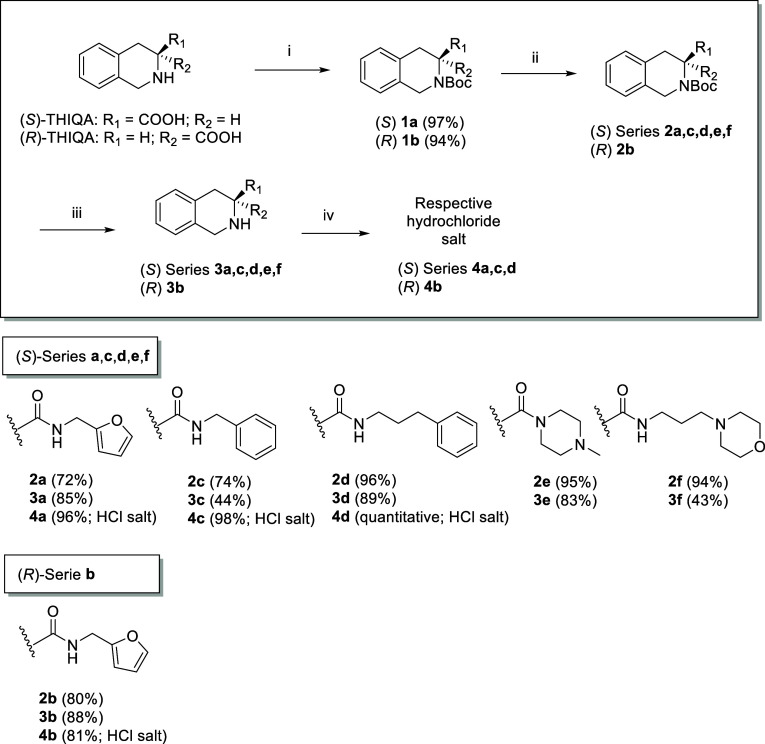
Synthesis Route Used for Preparation of THIQA Amides^a^

a Reaction conditions: (i) NaOH_(aq)_ 1 mol/L, (Boc)_2_O, THF; (ii) DMAP, EDAC, respective amine, DCM; (iii) TFA, 0 °C, DCM; (iv) HCl_(aq)_ 1 mol/L, acetone, 0 °C.

^1^H and ^13^C nuclear magnetic resonance (NMR) data confirmed the presence of the Boc group, indicating the successful protection of the amine nitrogen atoms in both THIQAs, affording **1a** (*S*-enantiomer) and **1b** (*R*-enantiomer) in excellent yields. It is worth highlighting that the characteristic signals for *tert*-butyl *N*-Boc groups are found duplicate in their respective NMR spectra. This phenomenon may be explained by the conformation variations of the *N*-Boc groups, and for that, in silico conformational analyses were performed.

The initial conformational analysis of **1a** and **1b** generated 8 conformers and 10 low-energy conformers using the molecular mechanics level of theory, respectively. After optimizing the geometry of all conformers with PM6 and density functional theory (DFT) levels of theory, we ordered the obtained conformations according to the calculated energies from the lowest to highest. At this point, we analyzed the differences between conformers and found two major conformational differences (molecular motions) for both compounds: (i) a “breathing” movement of the aliphatic ring (Figure 1, vertical transitions) similar to a boat-chair transition and (ii) the rotation of the nitrogen and carbonylic carbon atom bond (Figure 1, horizontal transitions). The other conformers (not shown) are just minor variations of the carboxylic acid position and intermediates of the “breathing” transition (one carbon positioned in front of the ring and the nitrogen atom behind, or vice versa, present only in **1b**). Interestingly, the first movement ranges between 0.33 and 0.62 kcal/mol means that they practically coexist in energy terms. The second movement occurs in an energy window lower than 5 kcal/mol. Therefore, both movements contribute to the *tert*-butyl position and affect the NMR signals.

**Figure 1:**
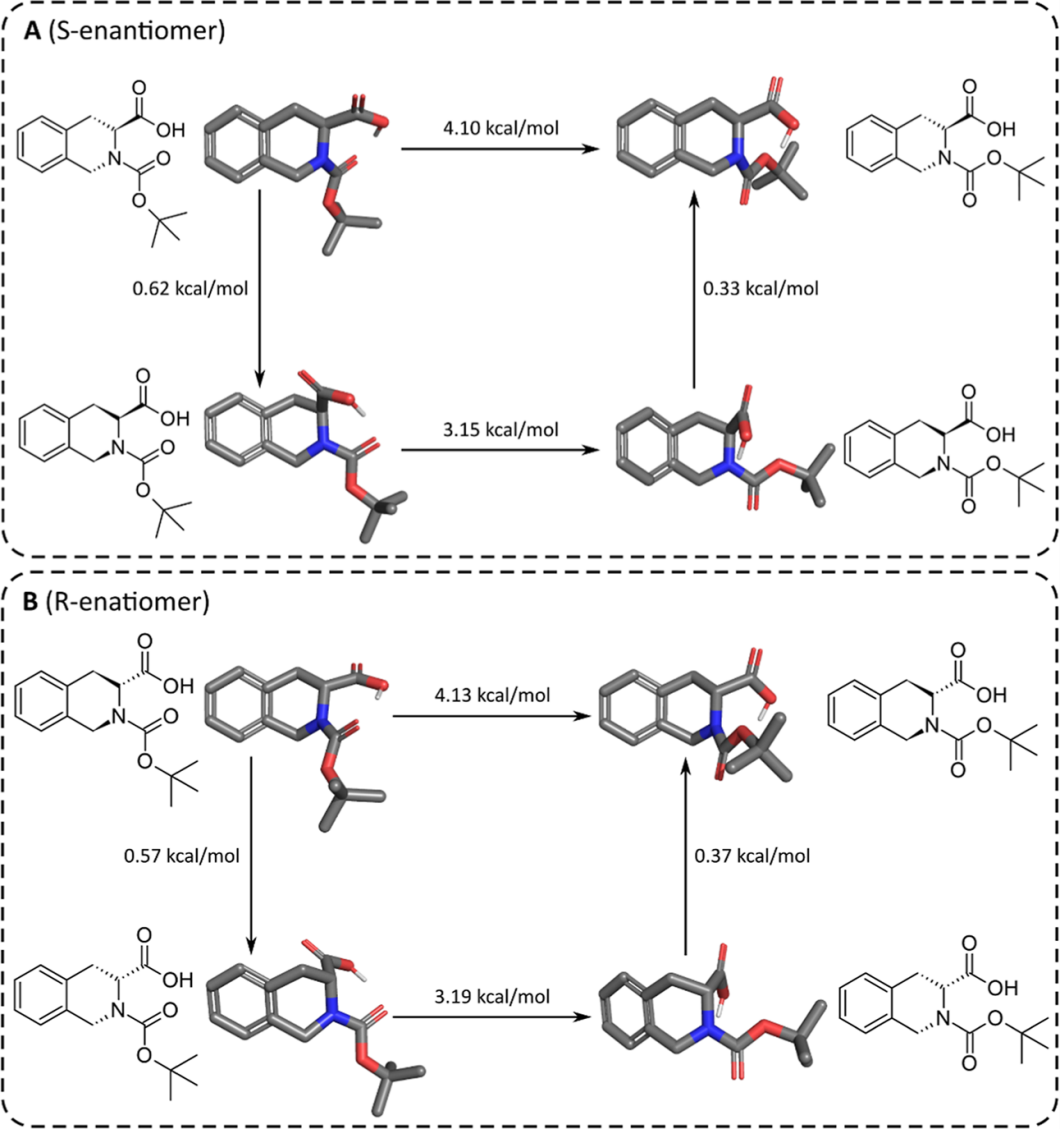
Conformational analysis of **1a** and **1b** (A and B, respectively) showing the most representative conformers and their transition energies.

The second step of the proposed synthetic route consisted of the formation of amides **2a**–**f** with different amines, using EDAC as a coupling reagent and DMAP as a catalyst, with yielding ranging from 72 to 96%, as shown in Scheme 1.

The presence of the amide side chains also contributes to the duplication of *N*-Boc *tert*-butyl signals. Exemplified with 4-methylpiperazine amide **2e**, this compound generated 34 conformers at the molecular mechanics level of theory, as expected due to the larger and more flexible structure. We analyzed the 10 first lowest energy conformers also calculated with PM6 followed by DFT (Figure 2), which ranged 1.33 kcal/mol, suggesting the easy conversion between them. The main observed molecular movements are related to the flip of the amide bond of the piperazine ring. This motion indirectly influenced the position of the *tert*-butyl group. Furthermore, the minor conformation within this sampling comprised the flipping of the carbamate *N*-carbonyl bond with a lower-energy transition than that of **1a** and **1b** (1.24 kcal/mol). Both conformational effects change the position of the *tert*-butyl group and could affect the NMR spectra, generating the observed duplicated signals.

**Figure 2:**
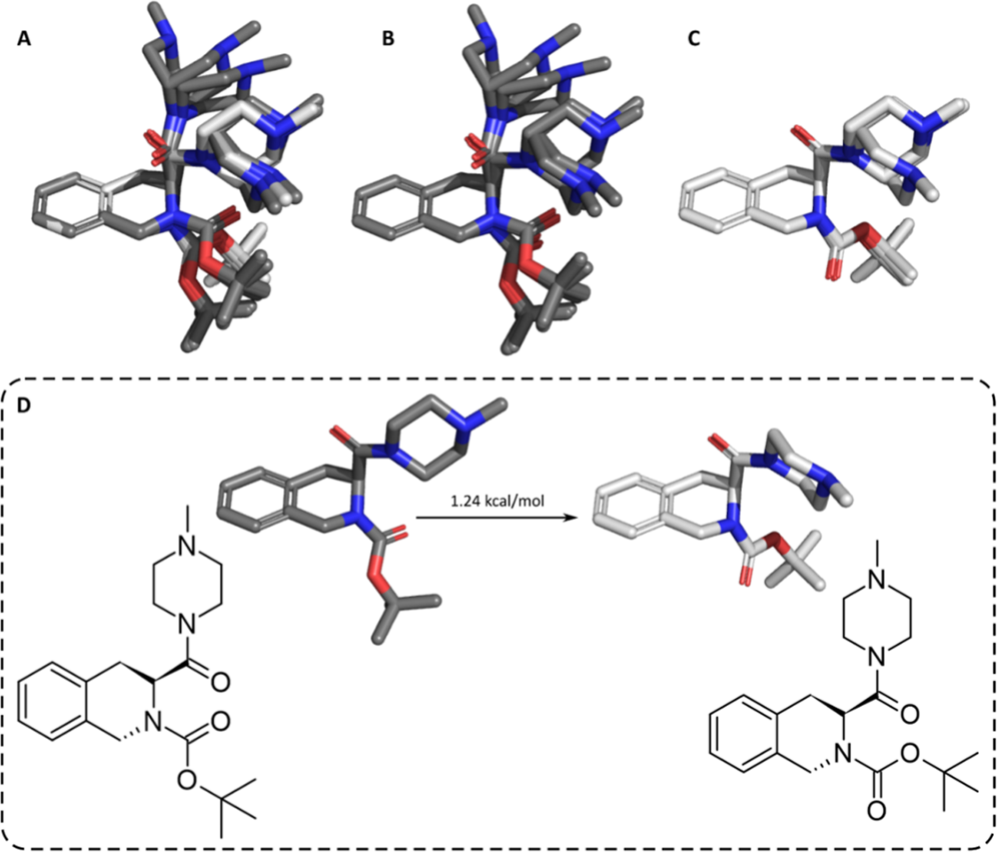
Conformational analysis of **2e**: superimposition of the 10 lowest energy conformers (A, major conformation is represented in dark gray carbons, and the minor conformation is represented in light gray carbons), the seven conformers with the conserved *N*-carbonyl bond (B) and the three others with this bond flipped (C). The transition between two states of the carbamate *N*-carbonyl bond and their transition energy (D).

Besides the duplicated specious NMR spectra due to the conformer equilibrium of *N*-Boc groups, another noteworthy feature related to the characterization of the synthesized compounds is the absence of a protector group in the mass spectra. With the exception of **2e** and **2f**, all the *N*-Boc compounds had lost their carbamoyl moiety in-source during the ESI-QTOF mass spectrometer analysis, leading to detection of the corresponding free tetrahydroisoquinoline amines only. Although electrospray ionization is well known to be a milder technique when it comes to fragmentation in comparison to harder ionization, for example, electron impact, this phenomenon may occur, especially to labile *N*-carbonyl bonds, such as the synthesized substances in the present work.

As the third step of the adopted synthetic route to obtain unprotected tetrahydroisoquinoline rings, the synthesized amides **2a**–**f** were subjected to reaction with trifluoroacetic acid (TFA) at low temperature in dichloromethane,^9^ affording **3a**–**f** as the final amides. Additionally, the hydrochlorides of furfuryl, benzyl, and 3-phenylpropylamides were obtained (**4a**–**d**) (Figure 3). This strategy is based on the improvement of their hydrosolubility and potential gain of new ionic molecular interactions (concerning the *N*-heteroatom).

**Figure 3:**
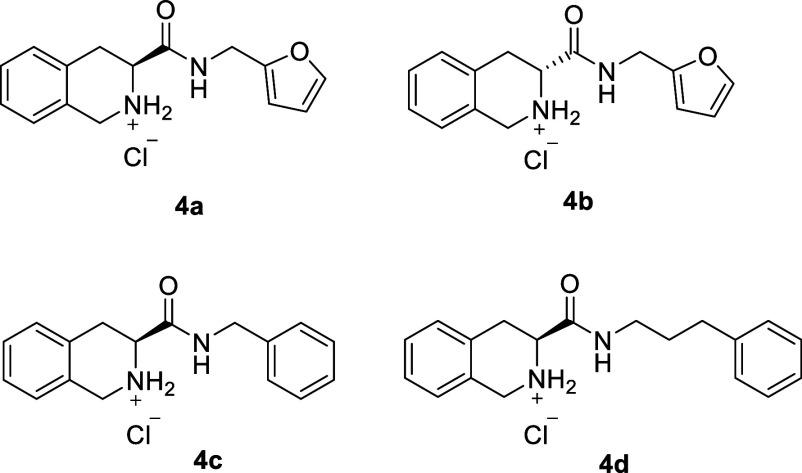
Chemical structures of synthesized hydrochloride salts.

Substances **3a–f** were assessed for their inhibitory potential toward cruzain and for their effects on intracellular amastigotes and bloodstream trypomastigotes of the β-galactosidase-expressing Tulahuen strain of *T. cruzi*. Compounds were initially evaluated in the cruzain assay after 10 min of preincubation with the enzyme (Table 1). The hydrochloride salts **4a** and **4b** exhibited the highest inhibition percentages, whereas all other analogues were essentially inactive. The compounds were subsequently tested without preincubation, and the inhibition values under both conditions were compared to assess time-dependent inhibition, typically associated with slow-binding or covalent inhibitors. However, no meaningful time-dependent inhibition was observed for any compounds, suggesting noncovalent binding.

**Table 1:** Substance Inhibition against Cruzain

substance	Cruzain inhibition at 100 μM^b^ (mean ± SEM %)	
	0′	10′ inc
**3a**	9.3 ± 1.7	16.3 ± 0.8
**3b**	ND^a^	6.4 ± 0.7
**3c**	ND^a^	14.1 ± 1.9
**3d**	ND^a^	14.1 ± 3.5
**3e**	ND^a^	8.4 ± 2.8
**3f**	ND^a^	9.6 ± 4.0
**4a** ^c^	55.0 ± 1.0	60.2 ± 2.4
**4b** ^c^	47.9 ± 5.3	69.3 ± 2.6
**4c** ^c^	ND^a^	14.5 ± 3.8
**4d** ^c^	ND^a^	15.0 ± 2.8
**E64** ^d^	73.4 ± 3.1	100 ± 5.3

a ND – nondetermined.

b Inhibition of cruzain at 100 μM for each compound following a 10 min preincubation with the enzyme (10′) or without preincubation (0′). Mean over two independent experiments, in triplicate ± standard error of the mean.

c Hydrochloride salt.

d E64 – epoxy succinate (positive control of inhibition).

Analysis of the structure–activity relationship (SAR) demonstrated that the tetrahydroisoquinoline and furan rings are important for enzyme inhibition, which likely accounts for the low activity of compounds **3c**–**f**, **4c**, and **4d**. Although **4a** and **4b** were active, the free bases (**3a** and **3b**) showed very low inhibition, suggesting that the positively charged amino group may be important for activity or solubility under the assay conditions.

In previous work, the commercially acquired racemic mixture of THIQA-furfurylamides (**4a** + **4b**) was reported as a potent competitive cruzain inhibitor (100 ± 2% inhibition at 100 μM; IC_50_ = 3.0 ± 2.0 μM).^7^ Surprisingly, in the present study, the pure synthesized and characterized isomers exhibited lower potencies, with IC_50_ values of 90 ± 13 μM for **4a** and 53 ± 5 μM for **4b** (Figure 4). Due to this result, we also tested a 1:1 mixture of the two isomers; however, we observed intermediate cruzain inhibition values when compared to individual isomers. Thus, the difference observed cannot be explained based on any synergistic effect involving the isomers and might be related to unknown differences between the commercially acquired samples and the ones synthesized in this study.

**Figure 4:**
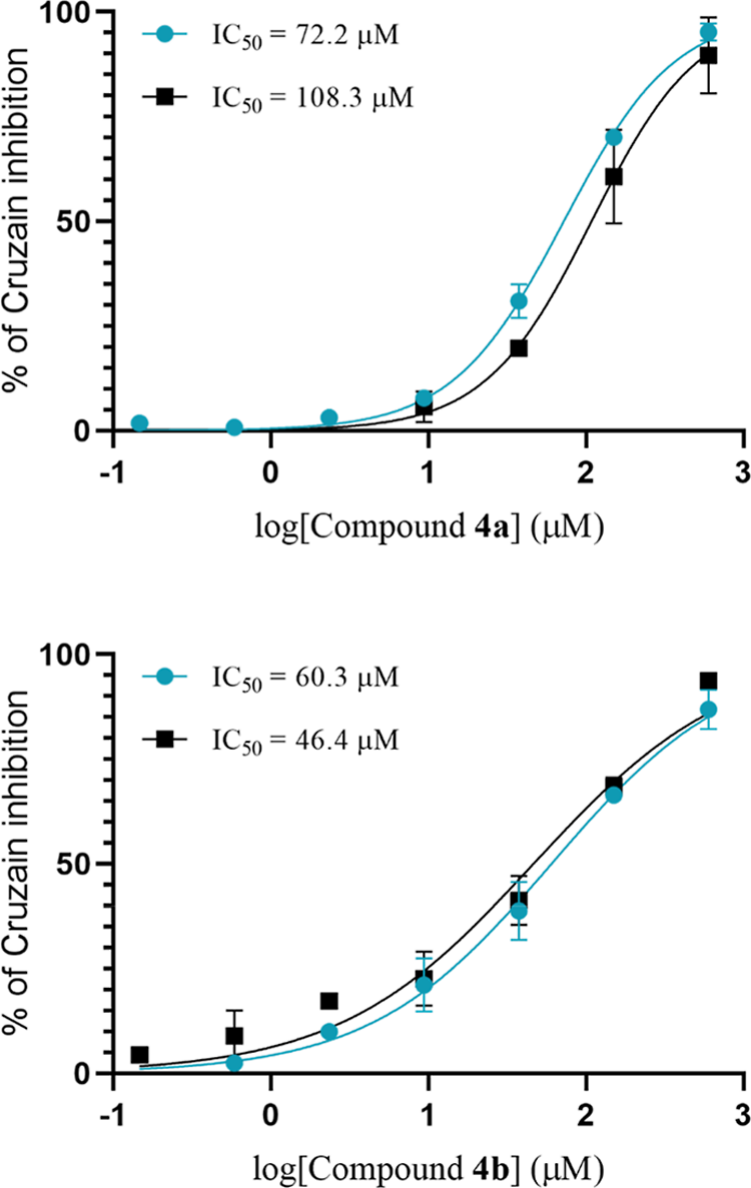
IC_50_ curves for compounds **4a** and **4b** against cruzain. Curves were obtained with a 10 min (10′) preincubation of the compound with the enzyme. Two independent experiments were performed: experiment 1 (blue curves) and experiment 2 (black curves).

In the phenotypic assays against *T. cruzi*, L929 fibroblasts were infected with 10 trypomastigotes per cell, and the intracellular infection was allowed to progress for 2 days. During this period, internalized trypomastigotes differentiated into intracellular amastigotes, ensuring that both early intracellular trypomastigotes and replicative amastigotes were present in the culture. The test compounds were then added for an additional four day incubation period, allowing the determination of inhibitory activity across the full intracellular developmental cycle of the parasite. Thus, the IC_50_ values reported here represent the compounds’ cumulative phenotypic activity against both intracellular trypomastigotes and amastigotes. The compounds were tested in serial dilutions prepared from freshly made stock solutions (20 mg/mL in DMSO). The results of the anti-*T. cruzi* assays are summarized in Table 2. IC_50_ and CC_50_ values were calculated only for compounds that exhibited ≥ 70% inhibition at 100 μg/mL, following the standard screening cascade for *T. cruzi* drug discovery.

**Table 2:** Substance Percentage of Inhibition of Parasitic Growth and Cell Death

substance	IC_50_ ^a^ (μM)	CC_50_ ^b^ (μM)	SI^c^
**3a**	ND^d^	ND^d^	ND^d^
**3b**	ND^d^	ND^d^	ND^d^
**3c**	211.4 ± 23.6	478.0	2.2
**3d**	10.5 ± 0.3	184.1	17.5
**3e**	ND^d^	ND^d^	ND^d^
**3f**	ND^d^	ND^d^	ND^d^
**4a** ^e^	79.6 ± 0.0	ND^d^	ND^d^
**4b** ^e^	32.4 ± 0.0	ND^d^	ND^d^
**4c** ^e^	227.5 ± 12.2	210.0	0.9
**4d** ^e^	13.7 ± 0.0	390.8	28.5
benznidazole	3.8	2401.7	625

a IC_50_, concentration of substance that reduces parasitic growth by 50%.

b CC_50_, concentration of substance that induces 50% cell death (L929).

c SI, selectivity index, calculated by the ratio CC_50_/ IC_50_.

d ND, nondetermined.

e Hydrochloride salt.

The most promising results were obtained for substances **3d** and **4d** with IC_50_ values of 10.5 ± 0.3 and 13.7 ± 0.0 μM, respectively, as well as for **4b**, which exhibited an IC_50_ of 32.4 ± 0.0 μM. Additionally, substances **3d** and **4d** presented selectivity indices significantly higher than those of the other substances, with values of 17.5 and 28.5, respectively. In contrast, substances **3d** and **4d** presented low inhibitory activity against cruzain (14.1 ± 3.5% for the free base and 15.0 ± 2.8% for the hydrochloride), suggesting that their antiparasitic effects may be mediated through alternative mechanisms. Substances **3c** and **4c** exhibited low activity, with IC_50_ values of 211.4 ± 23.6 and 227.5 ± 12.2, respectively. They also displayed extremely low selectivity indices (0.9 to 2.2), indicating that although they showed some inhibitory effect on *T. cruzi* growth, they also induced marked cytotoxicity, suggesting an unfavorable toxicity profile.

Although cruzain was initially evaluated as a potential molecular target, the absence of inhibitory activity for the most active compounds suggests that this protease is unlikely to be the primary target responsible for the observed anti-*T. cruzi* effects. At this stage, the molecular target underlying the intracellular activity remains to be elucidated, and proposing a specific mechanism of action would be speculative without further experimental validation. Alternative targets relevant to Chagas disease drug discovery include proteases other than cruzain, the sterol biosynthesis pathway, the antioxidant defense system, the mitochondrial electron transport chain, and pathways involved in mRNA processing, as discussed in a recent review by our group.^8^

## 3 Experimental Section

### 3.1 Chemistry

The NMR analyses were carried out by using a Bruker AVANCE NEO spectrometer operating at 600 MHz (Bruker, MA, USA). Tetramethylsilane served as the internal standard. Chemical shifts are reported as δ values in parts per million (ppm), while spin–spin coupling constants (*J*-values) are expressed in hertz (Hz). Signal multiplicities were described as s (singlet), bs (broad singlet), d (doublet), dd (double doublet), t (triplet), td (triplet of doublets), qt (quintet), or m (multiplet). Compounds **1a**,^9^ **1b**,^10^ **2c** and **3c**,^11^ and **2e** and **3e** ^12^ have been previously reported in the literature, and their synthetic routes were reproduced with minor adaptations based on the described procedures.

UHPLC–HRMS/MS experiments were carried out using a Nexera UPLC-system (Shimadzu) coupled to a maXis ESI-QTOF high-resolution mass spectrometer (Bruker), controlled with Compass 1.7 software (Bruker). Chromatographic separation was achieved on a Shim-Pack XR-ODS-III reversed-phase column (C18, 2.2 μm, 2.0 × 150 mm) at 40 °C, with a constant flow of 0.40 mL min^–1^. Elution was performed with a binary solvent system composed of Milli-Q water containing 0.1% formic acid (phase A) and acetonitrile with 0.1% formic acid (phase B), using the following gradient: 5% B maintained for 0.5 min, a linear increase to 100% B over 10 min, and an isocratic step at 100% B for an additional 1.5 min. UV-PDA detection was conducted in the 190–450 nm range, after which mass spectra were recorded in positive ion mode over an *m*/*z* interval of 100–1500, with a spectral acquisition frequency of 5 Hz. The electrospray source was operated with an end-plate offset of 500 and 4500 V capillary voltage. Nebulization was achieved at 3.0 bar, while dry gas flow was delivered at 8 L min^–1^ and maintained at 200 °C. Fragmentation data were recorded in a data-dependent acquisition mode using collision energies ranging from 15 to 60 eV. Mass accuracy was ensured through external calibration performed by direct infusion of 20 μL of a 1 mM sodium formate solution prepared in 50% 2-propanol, followed by postacquisition recalibration of the acquired data sets. Compound detection was performed using chromatographic peak dissection with subsequent formula determination based on exact mass and isotope pattern.

All reagents were obtained from Sigma-Aldrich (Missouri, USA) and employed as received, without additional purification steps.

#### 3.1.1 General Methodology for the Synthesis of Substances **2a**–**2f**

To a 50 mL round-bottom flask was added 1 equiv of the *N*-Boc-THIQA (**1a** or **1b**), 1 equiv of respective amine, and catalytic amount of 4-dimethylaminopyridin (DMAP) and dichloromethane. The mixture was kept under magnetic stirring and 0 °C and then 1 equiv of 1-ethyl-3-(3-(dimethylamino)­propyl)­carbodiimide hydrochloride (EDAC.HCl). The reaction was kept under low temperature for 20 min; then, ice bath was discontinued, and the mixture was allowed to warm to ambient conditions. Reaction progress was followed by thin-layer chromatography using an ethyl acetate/hexane (1:1, v/v) solvent system, with visualization performed by using iodine vapor and CAM staining. Once the starting material was no longer detected, dichloromethane (15 mL) was added, and the reaction mixture was transferred to a separatory funnel. The organic phase was subsequently washed with distilled water in three successive portions of 15 mL. The organic layer was dried over anhydrous Na_2_SO_4_ filtered, and the solvent was removed under reduced pressure, obtaining the desired product.

#### 3.1.2 *tert*-Butyl (*S*)-3-((Furan-2-ylmethyl)­carbamoyl)­3,4-dihydroisoquinoline-2­(1*H*)-carboxylate (**2a**)

According to the general methodology described in 3.1.1, the reaction was conducted using 505 mg (1.8 mmol) of carboxylic acid **1a**, 177 μL (194 mg, 2 mmol, 1.1 equiv) of furfurylamine, 3 mg of DMAP, and 5 mL of dichloromethane. After the reaction mixture reached 0 °C, 286 mg (1.5 mmol, 0.82 equiv) of EDAC.HCl was added. The reaction was kept under an inert N_2_ atmosphere, magnetic stirring at room temperature for 5 h, to obtain 467 mg (72% yield) of 2a as an orange pasty solid. [α]_D_ (MeOH, 1% w/v): −22°; ^1^H NMR (600 MHz, CDCl_3_), δ/ppm: 7.18–7.03 (m, 5H, H-1, H-2, H-3, H-6 and H-15); 6.15 (m, 1H, H-14); 5.86 (s, 1H, H-13); 4.80–4.12 (m, 5H, H-7, H-8 and H-11); 3.24–3.00 (m, 2H, H-9); 2.25–2,17 (m, 1H, CONH); 1.42–1.32 (m, 9H, H-18, H-19 and H-20); ^13^C NMR (150 MHz, CDCl_3_), δ/ppm: 142.2 (C-15); 134.0 (C-4 or C-5); 128.0–126.3 (C-1, C-2, C-3 and C-6); 110.5 (C-13 and C-14); 81.5 (C-17); 56.9 (C-8); 44.7 (C-7); 36.3 (C-11); 32.1 (C-9); 28.6 and 28.4 (C-18, C-19 and C-20); HRMS (*m*/*z*) [M + H]^+^ calcd for C_15_H_17_N_2_O_2_^+^, 257.1285 (without Boc group); found, 257.1287.

#### 3.1.3 *tert*-Butyl (*R*)-3-((Furan-2-ylmethyl)­carbamoyl)­3,4-dihydroisoquinoline-2­(1*H*)-carboxylate (**2b**)

According to the general methodology described in 3.1.1, the reaction was conducted using 496 mg (1.8 mmol) of carboxylic acid **1b**, 160 μL (176 mg, 1.8 mmol, 1 equiv) of furfurylamine, 10 mg of DMAP, and 7 mL of dichloromethane. After the reaction mixture reached 0 °C, 279 mg (1.5 mmol, 0.8 equiv) of EDAC.HCl was added. The reaction was kept under magnetic stirring at room temperature for 5 h to obtain 510 mg (80% yield) of **2b** as an orange pasty solid. [α]_D_ (MeOH, 1% w/v): +20°; ^1^H NMR (600 MHz, CDCl_3_), δ/ppm: 7.25–7.10 (m, 5H, H-1, H-2, H-3, H-6 and H-15); 6.23 (m, 1H, H-14); 5.93 (s, 1H, H-13); 4.88–4.20 (m, 5H, H-7, H-8 and H-11); 3.33–3.03 (m, 2H, H-9); 2.26–2.16 (m, 1H, CONH); 1.50–1.40 (m, 9H, H-18, H-19 and H-20); ^13^C NMR (150 MHz, CDCl_3_), δ/ppm: 142.2 (C-15); 134.0 (C-4 or C-5); 128.0–126.4 (C-1, C-2, C-3 and C-6); 110.4 (C-13 and C-14); 81.5 (C-17); 56.9 (C-8); 44.7 (C-7): 36.4 (C-11); 32.2 (C-9); 28.6 and 28.4 (C-18, C-19 and C-20); HRMS (*m*/*z*) [M + H]^+^ calcd for C_15_H_17_N_2_O_2_^+^, 257.1285 (without Boc group); found, 257.1284.

#### 3.1.4 *tert*-Butyl (*S*)-3-(Benzylcarbamoyl)-3,4-dihydroisoquinoline-2­(1*H*)-carboxilate (**2c**)

According to the general methodology described in 3.1.1, the reaction was conducted using 752 mg (2.7 mmol) of carboxylic acid **1a**, 300 μL (294 mg, 2.7 mmol, 1 equiv) of benzylamine, 5 mg of DMAP, and 7 mL of dichloromethane. After the reaction mixture reached 0 °C, 425 mg (2.2 mmol, 0.8 equiv) of EDAC.HCl was added. The reaction was kept under magnetic stirring at room temperature for 6 h to obtain 740 mg (74% yield) of 2c as a yellow pasty solid. [α]_D_ (MeOH, 1% w/v): −20°; ^1^H NMR (600 MHz, CDCl_3_), δ/ppm: 7.19–6.72 (m, 9H, H-1, H-2, H-3, H-6, H-13, H-14, H-15, H-16 and H-17); 4.84–4.11 (m, 5H, H-7, H-8 and H-11); 3.31–2.94 (m, 2H, H-9); 2.18–2.06 (m, 1H, CONH): 1.42–1.35 (m, 9H, H-20, H-21, and H-22); ^13^C NMR (150 MHz, CDCl_3_), δ/ppm: 134.0 (C-4 or C-5); 128.6–126.4 (C-1, C-2, C-3, C-6, C-13, C-14, C-15, C-16, and C-17); 81.4 (C-19); 56.9 (C-8); 43.2 (C-7 or C-11); 32.3 (C-9); 28.5 and 28.4 (C-20, C-21 and C-22); HRMS (*m*/*z*) [M + H]^+^ calc for C_17_H_19_N_2_O^+^, 267.1492 (without Boc group); found, 267.1497.

#### 3.1.5 *tert*-Butyl (*S*)-3-((3-Phenylpropyl)­carbamoyl)-3,4-dihydroisoquinoline-2­(1*H*)-carboxilate (**2d**)

According to the general methodology described in 3.1.1, the reaction was conducted using 505 mg (2.7 mmol) of the carboxylic acid **1a**, 260 μL (247 mg, 1.8 mmol, 1 equiv) of 3-phenyl-1-propylamine, 6 mg of DMAP, and 8 mL of dichloromethane. After the reaction mixture reached 0 °C, 353 mg (1.8 mmol, 1 equiv) of EDAC.HCl was added. The reaction was kept under magnetic stirring at room temperature for 5 h to obtain 691 mg (96% yield) of **2d** as a yellow pasty solid. [α]_D_ (MeOH, 1% w/v): −12°; ^1^H NMR (600 MHz, acetone-*d*_6_), δ/ppm: 7.14–6.96 (m, 9H, H-1, H-2, H-3, H-6, H-15, H-16, H-17, H-18, H-19); 4.98–4.34 (m, 2H, H-7 and H-8); 3.26–2.81 (m, 7H, H-7, H-9, H-11, and H-13); 2.36–2.29 (m, 2H, H-12); 1.96 (s, 1H, CONH); 1.41–1.29 (m, 9H, H-22, H-23, and H-24); ^13^C NMR (150 MHz, acetone-*d*_6_), δ/ppm: 143.3 (C-14); 133.9–126.8 (C-1, C-2, C-3, C-6, C-15, C-16, C-17, C-18, and C-19); 81.8 and 80.8 (C-21); 58.7 and 57.3 (C-8); 46.2 and 45.4 (C-7); 39.9 and 39.7 (C-11); 33.8–30.7 (C-12, C-13, and C-9); 28.9–28.6 (C-22, C-23, and C-24); HRMS (*m*/*z*) [M + H]^+^ calcd for C_19_H_23_N_2_O^+^, 295.1805 (without Boc group); found, 295.1805.

#### 3.1.6 *tert*-Butyl (*S*)-3-(4-Methylpiperazine-1-carbonyl)-3,4-dihydroisoquinoline-2­(1*H*) Carboxylate (**2e**)

According to the general methodology described in 3.1.1, the reaction was conducted using 755 mg (2.7 mmol) of carboxylic acid **1a**, 310 μL (280 mg, 2.8 mmol, 1 equiv) of 1-methylpiperazine, 5 mg of DMAP, and 7 mL of dichloromethane. After the reaction mixture reached 0 °C, 526 mg (2.7 mmol, 1 equiv) of EDAC.HCl was added. The reaction was kept under magnetic stirring at room temperature for 5 h to obtain 927 mg (95% yield) of **2e** as a yellow pasty solid. [α]_D_ (MeOH, 1% w/v): −34°; ^1^H NMR (600 MHz, CDCl_3_), δ/ppm: 7.24–7.04 (m, 4H, H-1, H-2, H-3, and H-6); 5.27 (m, 0.5H, H-8); 4.90–4.79 (m, 1.5H, H-8 and H-7); 4.43–4.34 (m, 1H, H-7); 3.64–3.46 (m, 4H, H-11, and H-14); 3.01–2.92 (m, 2H, H-9); 2.40–2.07 (m, 7H, H-12, H-13, and H-15); 1.45–1.41 (m, 9H, H-18, H-19, and H-20); ^13^C NMR (150 MHz, CDCl_3_), δ/ppm: 133.5 (C-4 or C-5); 128.5–125.9 (C-1, C-2, C-3, and C-6); 80.8 (C-17); 55.4 and 55.0 (C-12 and C-13); 52.3 and 49.9 (C-8); 46.2 (C-15); 45.6, 44.8, 44.3, and 42.2 (C-7, C-11, and C-14); 31.4 and 30.7 (C-9); 28.6 (C-18, C-19, C-20); HRMS (*m*/*z*) [M + H]^+^ calcd for C_20_H_30_N_3_O_3_^+^, 360.2282; found, 360.2281.

#### 3.1.7 *tert*-Butyl (*S*)-3-((3-Morpholinopropyl)­carbamoyl)-3,4-dihydroisoquinoline-2­(1*H*) Carboxylate (**2f**)

According to the general methodology described in 3.1.1, the reaction was conducted using 519 mg (1.9 mmol) of carboxylic acid **1a**, 274 μL (270 mg, 1.9 mmol, 1 equiv) of morpholino-1-propylamine, 10 mg of DMAP, and 8 mL of dichloromethane. After the reaction mixture reached 0 °C, 359 mg (1.9 mmol, 1 equiv) of EDAC.HCl was added. The reaction was kept under magnetic stirring at room temperature for 21 h to obtain 708 mg (94% yield) of **2f** as a yellow pasty solid. [α]_D_ (MeOH, 1% w/v): −12°; ^1^H NMR (600 MHz, acetone-*d*_6_), δ/ppm: 7.12–7.05 (m, 4H, H-1, H-2, H-3, and H-6); 4.66–4.32 (m, 2H, H-7, and H-8); 3.46–3.43 (m, 3H, H-15 or H-16 and H-7); 3.17–2.94 (m, 6H, H-9, H-11 and H-15 or H-16); 2.16–1.94 (m, 7H, H-13, H-14, H-17, and CONH); 1.92 (qt, *J*_11,12_ and *J*_12,13_ = 2.25 Hz, 2H, H-12); 1.40–1,30 (m, 9H, H-20, H-21, H-22); ^13^C NMR (150 MHz, acetone-*d*_6_), δ/ppm: 128.9–127.2 (C-1, C-2, C-3, and C-6); 80.8 (C-19); 67.7 (C-15 and C-16); 64.5 or 57.5 and 57.3 (C-13); 54.9 (C-14 and C-17); 45.3 (C-7); 38.6 (C-11); 33.1 or 31.0 and 30.7 (C-12); 28.9 (C-20, C-21, C-22); 27.2 (C-9); HRMS (*m*/*z*) [M + H]^+^ calcd for C_22_H_34_N_3_O_4_^+^, 404.2544; found, 404.2548.

#### 3.1.8 General Methodology for the Synthesis of Substances **3a**–**3f**

To a 50 mL round-bottom flask was added the respective amide (**2a** to **2f**) and dichloromethane. The solution was kept under magnetic stirring and ice bath until 0 °C, and then, 2 mL of trifluoroacetic acid was added. The reaction was kept under these conditions and monitored by TLC (eluent: ethyl acetate; stain: iodine) until evidence of consumption of the starting material. Then, trifluoroacetic acid was first eliminated by blowing compressed air over the reaction mixture. The remaining material was then dissolved in dichloromethane (15 mL) and transferred to a separatory funnel. The organic phase was sequentially washed with distilled water (10 mL), 0.5 mol L^–1^ of aqueous NaOH solution (25 mL), a saturated aqueous NH_4_Cl solution (10 mL), and finally distilled water (10 mL). After drying over anhydrous Na_2_SO_4_, the solution was filtered and concentrated under reduced pressure to afford the desired products.

#### 3.1.9 (*S*)–*N*-(Furan-2-ylmethyl)-1,2,3,4-tetrahydroisoquinoline-3-carboxamide (**3a**)

According to the general methodology described in 3.1.8, the reaction was conducted using 400 mg of the amide **2a**, 7 mL of dichloromethane, and kept under magnetic stirring at 0 °C for 2 h after the addition of trifluoroacetic acid to obtain 244 mg (85% yield) of **3a** as an orange solid. m.p.: degradation before melting; [α]_D_ (MeOH, 1% w/v): −70°; ^1^H NMR (600 MHz, acetone-*d*_6_), δ/ppm: 7.44 (br s, 1H, H-15); 7.13–7.04 (m, 4H, H-1, H-2, H-3, and H-6); 6.34–6.33 (m, 1H, H-14); 6.23 (m, 1H, H-13); 4.41 (m, 2H, H-11); 4,03 (s, 2H, H-7, and H-8); 3.65 (dd, *J*_7,7’_ e *J*_7,NH_ = 7.65 Hz, 1H, H-7); 3.08 (dd, *J*_8,9_ = 7.2 Hz e *J*_9,9’_ = 16.8 Hz, 1H, H-9); 2,87 (dd, *J*_8,9_ = 9 Hz e *J*_9’,9_ = 15.6 Hz, 1H, H-9′); 2.09 (s, 2H, NH and CONH); ^13^C NMR (150 MHz, acetone-*d*_6_), δ/ppm: 173.1 (C-10); 153.8 (C-12); 143.2 (C-15); 136.6 (C-4 or C-5); 135.4 (C-4 or C-5); 130.2–127.0 (C-1, C-2, C-3, and C-6); 111.6 (C-13 or C-14); 107.9 (C-13 or C-14); 57.4 (C-8); 47.8 (C-7); 36.8 (C-11); 32.0 (C-9); HRMS (*m*/*z*) [M + H]^+^ calcd for C_15_H_17_N_2_O_2_^+^, 257.1285; found, 257.1285.

#### 3.1.10 (*R*)-*N*-(Furan-2-ylmethyl)-1,2,3,4-tetrahydroisoquinoline-3-carboxamide (**3b**)

According to the general methodology described in 3.1.8, the reaction was conducted using 400 mg of the amide **2b**, 6 mL of dichloromethane, and kept under magnetic stirring at 0 °C for 2 h after the addition of trifluoroacetic acid to obtain 253 mg (88% yield) of 3b as an orange solid. M.P.: degradation before melting; [α]_D_ (MeOH, 1% w/v): +80°; ^1^H NMR (600 MHz, acetone-*d*_6_), δ/ppm: 7.44 (br s, 1H, H-15); 7.11–7.01 (m, 4H, H-1, H-2, H-3, and H-6); 6,34 (dd, *J*_13,14_ = 2.1 Hz e *J*_14,15_ = 1.2 Hz, 1H, H-14); 6.22 (d, *J*_13,14_ = 3 Hz, 1H, H-13); 4.41 (d, *J*_CONH,11_ = 4.2 Hz, 2H, H-11); 3.96 (s, 2H, H-7, and H-8); 3.52 (dd, *J*_7,7_ = 5.4 Hz and *J*_7,NH_ = 4.8 Hz, 1H, H-7); 3.04 (dd, *J*_8,9_ = 4.8 Hz and *J*_9,9_ = 11.4 Hz, 1H, H-9); 2.81 (dd, *J*_8,9_ = 6 Hz and *J*_9,9_ = 10.2 Hz, 1H, H-9); 2.08 (s, 1H, CONH); 2.07–2.06 (m, 1H, NH); ^13^C NMR (150 MHz, acetone-*d*_6_), δ/ppm: 173.2 (C-10); 153.6 (C-12); 142.8 (C-15); 137.2 (C-4); 135.5 (C-5); 129.9–126.6 (C-1, C-2, C-3, and C-6); 111.2 (C-14); 107.5 (C-13); 57.3 (C-8); 47.9 (C-7); 36.4 (C-11); 31,8 (C-9); HRMS (*m*/*z*) [M + H]^+^ calcd for C_15_H_17_N_2_O_2_^+^, 257.1285; found, 257.1283.

#### 3.1.11 (*S*)–*N*-Benzyl-1,2,3,4-tetrahydroisoquinoline-3-carboxamide (**3c**)

According to the general methodology described in 3.1.8, the reaction was conducted using 671 mg of amide **2c**, 10 mL of dichloromethane, and kept under magnetic stirring at 0 °C for 4 h after the addition of 2.5 mL of trifluoroacetic acid. After the workup described in 3.1.8, the combined aqueous layers were extracted with dichloromethane (3 × 15 mL). The combined organic layers were dried over anhydrous Na_2_SO_4_, filtered, and concentrated under reduced pressure to yield 481 mg of crude material. A portion of this material (250 mg) was subsequently dissolved in dichloromethane (15 mL) and placed in a separatory funnel, where it was subjected to successive washes with distilled water (3 × 20 mL) followed by 1 mol L^–1^ of aqueous HCl (2 × 15 mL). The resulting aqueous layers were combined and then alkalinized with aqueous solution of NaOH 1 mol/L until pH 12 and then extracted with dichloromethane (5 × 20 mL). The organic layers obtained from this step were again combined and dried over anhydrous Na_2_SO_4_, filtered, and the solvent was removed under reduced pressure, obtaining 214 mg (44% yield) of **3c** as a beige solid. M.P.: 100.5–103.5 °C; [α]_D_ (MeOH, 1% w/v): −72°; ^1^H NMR (600 MHz, acetone-*d*_6_), δ/ppm: 7.31–7.28 (m, 4H, H-2, H-3, H-14, and H-16); 7.24–7.21 (m, 1H, H-15); 7.14–7.10 (m, 3H, H-1 or H-6, H-13, and H-17); 7.04–7.02 (m, 1H, H-1, or H-6); 4.46–4.40 (m, 2H, H-11); 3.97 (s, 2H, H-7, and H-8); 3.56 (dd, *J*_7,7_ = 5.4 Hz and *J*_7,NH_ = 4.8 Hz, 1H, H-7); 3.06 (dd, *J*_8,9_ = 4.8 Hz and *J*_9,9_ = 11.3 Hz, 1H, H-9); 2.85 (dd, *J*_8,9_ = 6.1 Hz e *J*_9,9_ = 11.3 Hz, 1H, H-9); 2.08–2.06 (m, 1H, NH); ^13^C NMR (150 MHz, acetone-*d*_6_), δ/ppm: 173.4 (C-10); 140.7 (C-4, C-5 or C-12); 137.3 (C-4, C-5, or C-12); 135.6 (C-4, C-5, or C-12); 129.9–126.6 (C-1, C-2, C-3, C6, C-13, C-14, C-15, C-16, and C-17); 57.4 (C-8); 47.9 (C-7); 43.2 and 43.1 (C-11); 32.0 (C-9); HRMS (*m*/*z*) [M + H]^+^ calcd for C_17_H_19_N_2_O^+^, 267.1492; found, 267.1493.

#### 3.1.12 (*S*)–*N*-(3-Phenylpropyl)-1,2,3,4-tetrahydroisoquinoline-3-carboxamide (**3d**)

According to the general methodology described in 3.1.8, the reaction was conducted using 270 mg of amide **2d**, 6 mL of dichloromethane and kept under magnetic stirring at 0 °C for 2 h after the addition of trifluoroacetic acid, obtaining 179 mg (89% yield) of **3d** as an orange pasty solid. [α]^D^ (MeOH, 1% w/v): −60°; ^1^H NMR (400 MHz, acetone-*d*_6_), δ/ppm: 7.25–7.10 (m, 9H, H-1, H-2, H-3, H-6, H-15, H-16, H-17, H-18, and H-19); 3.97 (s, 1.5H, H-8, and H-7); 3.54 (t, *J*_8,9_ = 7 Hz, 0.5H, H-8); 3.47 (dd, *J*_7,7_ = 5.2 Hz and *J*_7,NH_ = 4.8 Hz, 1H, H-7); 3.27 (td, *J*_11,12_ = 10.5 Hz e *J*_11,CONH_ = 5.4 Hz, 2H, H-11); 3.03 (dd, *J*_8,9_ = 4.8 Hz e *J*_9,9_ = 11.6 Hz, 1H, H-9); 2.80 (dd, *J*_8,9_ = 6 Hz e *J*_9,9_ = 10.4 Hz, 1H, H-9); 2.64 (t, *J*_12,13_ = 7.6 Hz, 2H, H-13); 2.09–2.07 (m, 2H, NH, and CONH); 1.83 (qt, *J*_12,13_ and *J*_12,13_ = 7.4 Hz, 2H, H-12); ^13^C NMR (150 MHz, acetone-*d*_6_), δ/ppm: 173.4 (C-10); 143.0 (C-14); 137.2 (C-4); 135.6 (C-5); 132.6–126.6 (C-1, C-2, C-3, C-6, C-15, C-16, C-17, C-18, C-19); 57.4 (C-8); 48.0 (C-7); 39.2 (C-11); 33.9 (C-9); 32.4 (C-13); 32,1 (C-12); HRMS (*m*/*z*) [M + H]^+^ calcd for C_19_H_23_N_2_O^+^, 295.1805; found, 295.1808.

#### 3.1.13 (*S*)-(4-Methylpiperazin-1-yl)­(1,2,3,4-tetrahydroisoquinolin-3-yl)­methanone (**3e**)

According to the general methodology described in 3.1.8, the reaction was conducted using 850 mg of amide **2e**, 10 mL of dichloromethane and kept under magnetic stirring at 0 °C for 2 h after the addition of 2.5 mL of trifluoroacetic acid, obtaining 511 mg (83% yield) of **3e** as an orange pasty solid. [α]_D_ (MeOH, 1% w/v): −52°; ^1^H NMR (400 MHz, acetone-*d*_6_), δ/ppm: 7.13–7.03 (m, 4H, H-1, H-2, H-3, and H-6); 4.06 (m, 3H, H-7, and H-8); 3.72–3.54 (m, 4H, H-11, and H-14); 2.91 (dd, *J*_8,9_ = 6 Hz and *J*_9,9_ = 10.4 Hz, 1H, H-9); 2.82 (dd, *J*_8,9_ = 5 Hz and *J*_9,9_ = 11.6 Hz, 1H, H-9); 2.45–2.36 (m, 4H, H-12, and H-13); 2.26 (s, 3H, H-15); 2.09–2.07 (m, 1H, NH); ^13^C NMR (100 MHz, acetone-*d*_6_), δ/ppm: 130.0–126.6 (C-1, C-2, C-3, and C-6); 56.1 and 55.4 (C-12 and C-13); 53.6 (C-8); 47.4 (C-7, C-11 or C-14); 46.2 (C-15); 46.0 (C-7, C-11 or C-14); 42.4 (C-7, C-11, or C-14); 31.7 (C-9).

#### 3.1.14 (*S*)–*N*-(3-Morpholinopropyl)-1,2,3,4-tetrahydroisoquinoline-3-carboxamide (**3f**)

According to the general methodology described in 3.1.8, the reaction was conducted using 650 mg of amide **2f**, 10 mL of dichloromethane and kept under magnetic stirring at 0 °C for 5 h after the addition of 2.5 mL of trifluoroacetic acid, obtaining 212 mg (43% yield) of **3f** as an orange pasty solid. [α]_D_ (MeOH, 1% w/v): −60°; ^1^H NMR (400 MHz, acetone-*d*_6_), δ/ppm: 7.13–7.03 (m, 4H, H-1, H-2, H-3, and H-6); 3.97–3.72 (m, 2H, H-7, and H-8); 3.65–3.63 (t, *J*_14,15_ and *J*_16,17_ = 4.6 Hz, 4H, H-15, and H-16); 3.45 (dd, *J*_7,7_ = 5.2 Hz and *J*_7,NH_ = 4.8 Hz, 1H, H-7); 3.29 (t, *J*_11,12_ = 6.6 Hz, 2H, H-11); 3.05 (dd, *J*_9,9_ = 11.6 Hz and *J*_9,8_ = 4.8 Hz, 1H, H-9); 2.78 (dd, *J*_8,9_ = 6 Hz e *J*_9,9_ = 10.2 Hz, 1H, H-9); 2.38 (m, 6H, H-13, H-14, and H-17); 1.67 (qt, *J*_11,12_ e *J*_12,13_ = 6.7 Hz, 2H, H-12); ^13^C NMR (100 MHz, acetone-*d*_6_), δ/ppm: 137.2 (C-4); 135.6 (C-5); 129.9–126.6 (C-1, C-2, C-3, and C-6); 67.4 (C-15 and C-16); 57.9 (C-13); 57.4 (C-8); 54.7 (C-14 and C-17); 48.0 (C-7); 38.6 (C-11); 32.0 (C-9); 26.6 (C-12); HRMS (*m*/*z*) [M + H]^+^ calcd for C_17_H_26_N_3_O_2_^+^, 304.2020; found, 304.2019.

#### 3.1.15 General Methodology for the Synthesis of Hydrochlorides **4a**, **4c,** and **4d**

The respective amide was solubilized in 15 mL of AcOEt, transferred to a separation funnel, and extracted with 3 × 20 mL of HCl 0.1 mol/L. The aqueous layers were combined and then dried over compressed air flow.

#### 3.1.16 (*S*)-3-((Furan-2-ylmethyl)­carbamoyl)-1,2,3,4-tetrahydroisoquinolin-2-ium Chloride (**4a**)

According to the general methodology described in 3.1.15, the reaction was conducted using 51 mg of amide **3a**, obtaining 56 mg (96% yield) of **4a** as a brownish solid. m.p.: degradation before melting; [α]_D_ (MeOH, 1% w/v): −90°; ^1^H NMR (600 MHz, DMSO-*d*_6_), δ/ppm: 10.13 (br s, 1H, NH_2_); 9.63 (br s, 1H, NH_2_); 9.36 (t, *J*_CONH,11_ = 5.4 Hz, 1H, CONH); 7.60–7.58 (m, 1H, H-15); 7.24 (s, 4H, H-1, H-2, H-3, and H-6); 6.41 (br s, 1H, H-13, or H-14); 6.34 (br s, 1H, H-13, or H-14); 4.38–4.20 (m, 5H, H-7, H-8, and H-11); 3.38–3.34 (m, 1H, H-9); 3.02 (dd, *J*_9,9_ = 12 Hz and *J*_9,8_ = 4 Hz, 1H, H-9); ^13^C NMR (150 MHz, DMSO-*d*_6_), δ/ppm: 167.7 (C-10); 151.3 (C-12); 142.3 (C-15); 131.0 (C-5); 128.6 (C-3); 128.5 (C-4); 127.4 (C-6); 126.8 (C-1 or C-2); 126.5 (C-1 or C-2); 110.5 (C-13 or C-14); 107.2 (C-13 or C-14); 53.8 (C-8); 43.7 (C-7); 35.6 (C-11); 29.3 (C-9).

#### 3.1.17 (*R*)-3-((Furan-2-ylmethyl)­carbamoyl)-1,2,3,4-tetrahydroisoquinolin-2-ium Chloride (**4b**)

To a 50 mL round-bottom flask containing 74 mg of **3b** solubilized in 10 mL of acetone was added, under ice bath, 1 mL of 1.0 mol/L HCl. The mixture was kept under magnetic stirring for 5 min and then dried over compressed air flow, obtaining 68 mg (81% yield) of **4b** as a brownish solid. M.P.: degradation before melting; [α]_D_ (MeOH, 1% w/v): +76°; ^1^H NMR (600 MHz, DMSO-*d*_6_), δ/ppm: 10.11 (s, 1H, NH_2_); 9.62 (s, 1H, NH_2_); 9.35 (t, *J*_CONH,11_ = 5.4 Hz, 1H, CONH); 7.61 (m, 1H, H-15); 7.27–7.22 (m, 4H, H-1, H-2, H-3, H-6); 6.42 (dd, *J*_13,14_ = 1.8 Hz and *J*_14,15_ = 1.2 Hz, 1H, H-14); 6.34 (m, 1H, H-13); 4.38 (t, *J*_7,NH_ = 5.4 Hz, 2H, H-11); 4.33–4.26 (m, 2H, H-7); 4.23–4.18 (m, 1H, H-8); 3.36 (dd, *J*_8,9_ = 4.2 Hz e *J*_9,9_ = 12.6 Hz, 1H, H-9); 3.02 (dd, *J*_8,9_ = 4.8 Hz e *J*_9,9_ = 12 Hz, 1H, H-9); ^13^C NMR (150 MHz, DMSO-*d*_6_), δ/ppm: 167.8 (C-10); 151,4 (C-12); 142.4 (C-15); 131.0 (C-4 and C-5); 128.6–126.6 (C-1, C-2, C-3 and C-6); 110.5 (C-13 or C-14); 107.3 (C-13 or C-14); 53.9 (C-8); 43.8 (C-7); 35.7 (C-11); 29.4 (C-9); HRMS (*m*/*z*) [M + H]^+^ calcd for C_15_H_17_N_2_O_2_^+^, 257.1285; found, 257.1287.

#### 3.1.18 (*S*)-3-(Benzylcarbamoyl)-1,2,3,4-tetrahydroisoquinolin-2-ium Chloride (**4c**)

According to the general methodology described in 3.1.15, the reaction was conducted using 100 mg of amide **3c**, obtaining 112 mg (98% yield) of **4c** as a beige solid. m.p.: 184.9–188,9 °C; [α]_D_ (MeOH, 1% w/v): −92°; ^1^H NMR (600 MHz, DMSO-*d*_6_), δ/ppm: 10.04 (br s, 1H, NH_2_); 9.57 (br s, 1H, NH_2_); 9.40 (t, *J*_CONH,11_ = 5.7 Hz, 1H, CONH); 7.35–7.25 (m, 9H, H-1, H-2, H-3, H-6, H-13, H-14, H-15, H-16, and H-17); 4.38–4.23 (m, 5H, H-7, H-8, and H-11); 3.39 (dd, *J*_8,9_ = 4.2 Hz and *J*_9,9_ = 12.6 Hz, 1H, H-9); 3.06 (dd, *J*_8,9_ = 4.2 Hz and *J*_9,9_ = 12 Hz, 1H, H-9); ^13^C NMR (150 MHz, DMSO-*d*_6_), δ/ppm: 167.9 (C-10); 138.6 (C-12); 131.0 (C-4 and C-5); 128.7–126.6 (C-1, C-2, C-3, C-6, C-13, C-14, C-15, C-16, and C-17); 54.0 (C-8); 43.9 (C-7 or C-11); 42.2 (C-7 or C-11); 29.4 (C-9); HRMS (*m*/*z*) [M + H]^+^ calcd for C_17_H_19_N_2_O^+^, 267.1492; found, 257.1493.

#### 3.1.19 (*S*)-3-((3-Phenylpropyl)­carbamoyl)-1,2,3,4-tetrahydroisoquinolin-2-ium Chloride (**4d**)

According to the general methodology described in 3.1.15, the reaction was conducted using 109 mg of amide **3d**, obtaining 122 mg (quantitative yield) of **4d** as a white solid. m.p.: degradation before melting; [α]_D_ (MeOH, 1% w/v): −90°; ^1^H NMR (600 MHz, DMSO-*d*_6_), δ/ppm: 10.10 (br s, 1H, NH_2_); 9.54 (br s, 1H, NH_2_); 9.00 (t, *J*_CONH,11_ = 5.4 Hz, 1H, CONH); 7.30–7.17 (m, 9H, H-1, H-2, H-3, H-6, H-15, H-16, H-17, H-18, and H-19); 4.34–4.17 (m, 3H, H-7, and H-8); 3.36 (dd, *J*_8,9_ = 4.2 Hz and *J*_9,9_ = 12.6 Hz, 1H, H-9); 3.18–3.16 (m, 2H, H-11); 3.02 (dd, *J*_8,9_ = 4.2 Hz and *J*_9,9_ = 12 Hz, 1H, H-9); 2.63 (t, *J*_12,13_ = 7.5 Hz, 2H, H-13); 1.77 (qt, *J*_11,12_ and *J*_12,13_ = 7.2 Hz, 2H, H-12); ^13^C NMR (150 MHz, DMSO-*d*_6_), δ/ppm: 167.6 (C-10); 141.6 (C-14); 131.1 (C-4 or C-5); 128.6–125.8 (C-1, C-2, C-3, C-6, C-15, C-16, C-17, C-18, and C-19); 53.9 (C-8); 43.7 (C-7); 38.3 (C-11); 32.4 (C-9); 30.7 (C-12 or C-13); 29.4 (C-12 or C-13).

### 3.2 In Silico Studies

We analyzed the properties of THIQA derivatives using a computational workflow designed to compare the relevant conformations of those compounds. Initially, conformational analysis was performed using the SPARTAN’20 V1.1.4 software,^13^ employing the MMFF94s force field^14^ to generate a diverse set of low-energy conformers. All conformers generated in this step were retained for further analysis to capture the potential conformational flexibility. Each of these conformers is then subjected to geometry optimization using the semiempirical PM6 method^15^ Then, another geometry optimization was carried out using DFT at the B3LYP functional^16–18^ with the 6-31G* basis set.^19,20^

### 3.3 Biological Assays

#### 3.3.1 Cruzain Inhibition Assay

Recombinant cruzain was kindly provided by Allison Doak and Brian Shoichet (University of California, San Francisco, CA, USA). Cruzain inhibition assays were performed according to previously reported procedures.^21,22^ Briefly, enzymatic activity was monitored through the hydrolysis of the fluorogenic substrate Z-Phe-Arg-AMC using recombinant cruzain in sodium acetate buffer (pH 5.5) supplemented with β-mercaptoethanol and Triton X-100. Fluorescence was recorded over time at appropriate excitation (340 nm) and emission (440 nm) wavelengths with DMSO and E-64 employed as negative and positive controls, respectively. Compounds were initially screened at 100 μM, and inhibition percentages were calculated from initial reaction rates in the presence of compounds to those observed for the DMSO control. IC_50_ values were obtained by nonlinear regression analysis, with all measurements performed in triplicate in independent experiments.

#### 3.3.2 In Vitro Activity against *T. cruzi* Intracellular Amastigotes and Early Trypomastigotes

The in vitro anti-*T. cruzi* activity was evaluated on L929 mouse fibroblasts infected with Tulahuen strain of the *T. cruzi* expressing the *Escherichia coli* β-galactosidase as a reporter gene, following the procedure described previously.^23^ This assay format allows for the assessment of compound activity across the intracellular developmental cycle of the parasite, including the early trypomastigote stage and the replicative amastigote stage. Briefly, for the bioassay, 4000 L929 cells were added to each well of a 96-well microtiter plate. After an overnight incubation, 40,000 trypomastigotes were added to the cells and incubated for 2 h. Then, the medium containing extracellular parasites was replaced with 150 μL of fresh medium, and the plate was incubated for an additional 48 h to establish the infection. During this period, internalized trypomastigotes naturally differentiate into amastigotes, while extracellular parasites are eliminated; thus, both early intracellular trypomastigotes and amastigotes are present within host cells. For IC_50_ determination, the cells were exposed to each synthesized compound at serial decreasing dilutions, and the plate was incubated for 96 h. This extended exposure period covers the full intracellular cycle of *T. cruzi*, enabling phenotypic evaluation of the compound activity against both parasite stages. After this period, 50 μL of 500 μM chlorophenol red beta-d-galactopyranoside (CPRG) in 0.5% Nonidet P40 was added to each well, and the plate was incubated for 16–20 h, after which the absorbance at 570 nm was measured. Controls with uninfected cells, untreated infected cells, infected cells treated with benznidazole at 3.8 μM (1 μg/mL—positive control), or DMSO 1% were used. The results were expressed as the percentage of *T. cruzi* growth inhibition in compound tested cells compared to that of the infected cells and untreated cells. The IC_50_ values were calculated by linear interpolation. Quadruplicates were run on the same plate, and the experiments were repeated at least once.

#### 3.3.3 In Vitro Cytotoxic Activity Test of Compounds and CC_50_ Determination over L929 Cell Line

For this assay, L929 fibroblasts (4000 cells per well) were seeded in 96-well microplates containing 150 μL of RPMI-1640 medium (pH 7.2–7.4; Gibco BRL) supplemented with 10% fetal bovine serum and 2 mM glutamine and incubated at 37 °C for 3 days. The culture medium was subsequently replaced, and the cells were treated with increasing concentrations of the test compounds, starting at the IC_50_ value determined for *T. cruzi*. Following 96 h of exposure, alamarBlue was added, and absorbance was measured at 570 and 600 nm after 4–6 h. Untreated cells and cells exposed to 1% DMSO were included as controls. Cell viability was calculated based on the differential reduction of alamarBlue between treated and control wells, and the concentration required to reduce cell viability by 50% (CC_50_) was determined. Quadruplicates were run in the same plate, and the experiments were repeated at least once.

IC_50_ over *T. cruzi* and L929 cells were determined by linear interpolation, and the selectivity index (Supporting Information) was calculated by the ratio of CC_50_ L929 cells/IC_50_ *T. cruzi*.

## 4 Conclusion

In the present study, we synthesized and characterized six final amide derivatives of 1,2,3,4-tetrahydroisoquinoline-3-carboxylic acid (**3a**–**f**) and four hydrochlorides (**4a**–**d**). The compounds were subjected to enzymatic and cellular assays to evaluate their activity against cruzain and *T. cruzi*. Among the tested molecules, **4a** and **4b** displayed the highest cruzain inhibition, and their IC_50_ values were determined. However, both compounds exhibited low potency when compared to commonly applied hit-identification (IC_50_ < 5 μM). In cellular assays, **3d** and **4d** showed the best activity against *T. cruzi*, with IC_50_ values comparable to those of the positive control benznidazole. Furthermore, the activity of these substances against *T. cruzi* appears to be independent of cruzain inhibition since their inhibition percentages were low. In light of the modest activity observed against cruzain, structural optimization is required in an effort to obtain more potent inhibitors. Accordingly, additional modifications—such as alterations to the tetrahydroisoquinoline ring, introduction of strategic substituents, and optimization of the side chain—may further enhance the potency of these 1,2,3,4-tetrahydroisoquinoline amide derivatives toward this enzyme.

Moreover, substance **3d** showed excellent results against *T. cruzi* and may be a candidate for further testing, such as in vivo tests. Furthermore, studies can be carried out to investigate by which mechanism this substance inhibits parasite growth.

## Supplementary Material



## Notes

Spectroscopic characterization of synthesized compounds (NMR and HRMS) (PDF)
