# Supplementary material for: Synthesis and Biological Evaluation of Tetrahydroisoquinoline Derivatives as Trypanocidal Agents
Source: ACS Omega. 2026 Jan 21;11(4):6275–84. doi: 10.1021/acsomega.5c11033 (PMC12878744; doi:10.1021/acsomega.5c11033)

SUPPORTING INFORMATION

Synthesis and Biological Evaluation of Tetrahydroisoquinoline Derivatives as Trypanocidal Agents

João Paulo de Moura Lopes,[a] Gabriel Vitor de Lima Marques,[a] Lucas Abreu Diniz,[b] Viviane Corrêa Santos,[b,d] Daniela de Melo Resende,[c] Silvane Maria Fonseca Murta,[c] Markus Kohlhoff,[c] Vinícius Gonçalves Maltarollo,[a] Rafaela Salgado Ferreira,[b] and Renata Barbosa Oliveira[a]*

[a]Departamento de Produtos Farmacêuticos, Universidade Federal de Minas Gerais, Belo Horizonte, Brazil

[b]Departamento de Bioquímica e Imunologia, Universidade Federal de Minas Gerais, Belo Horizonte, Brazil

[c]Instituto René Rachou - FIOCRUZ Minas, Belo Horizonte, Brazil

[d]Department of Chemistry, Grand Valley State University, Allendale, Michigan 49401, United States

*Corresponding authors: Tel: +553134096395; fax: +553134096935.

E-mail address: renatabo.ufmg@gmail.com

(*S*)-2-(tert-butoxycarbonyl)-1,2,3,4-tetrahydroisoquinoline-3-carboxylic acid (**1a**)

^{1}H NMR (600 MHz, $CDCl_3$)

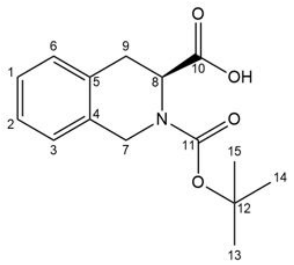

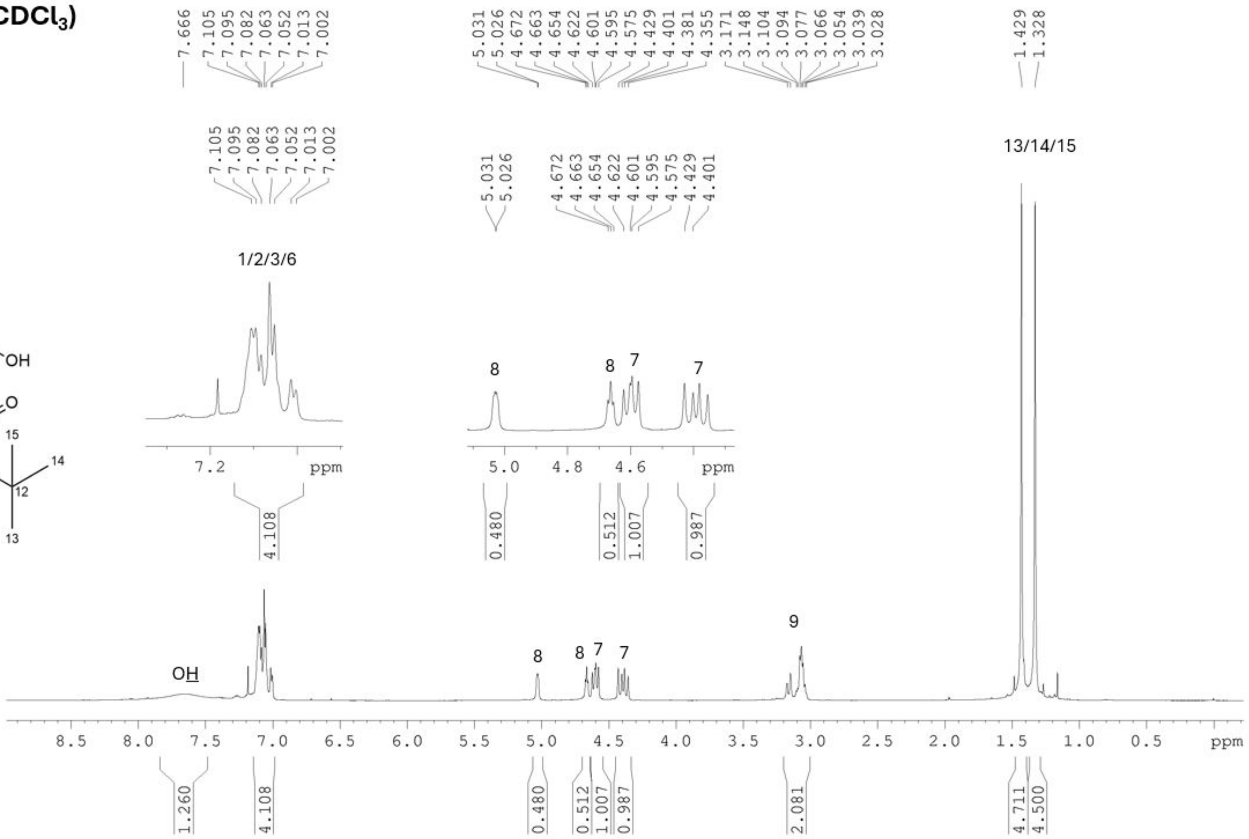

^{13}C NMR/DEPT135 (150 MHz, $CDCl_3$)

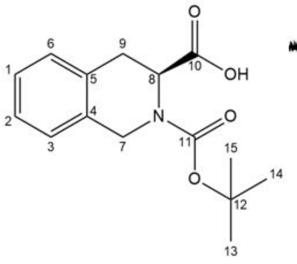

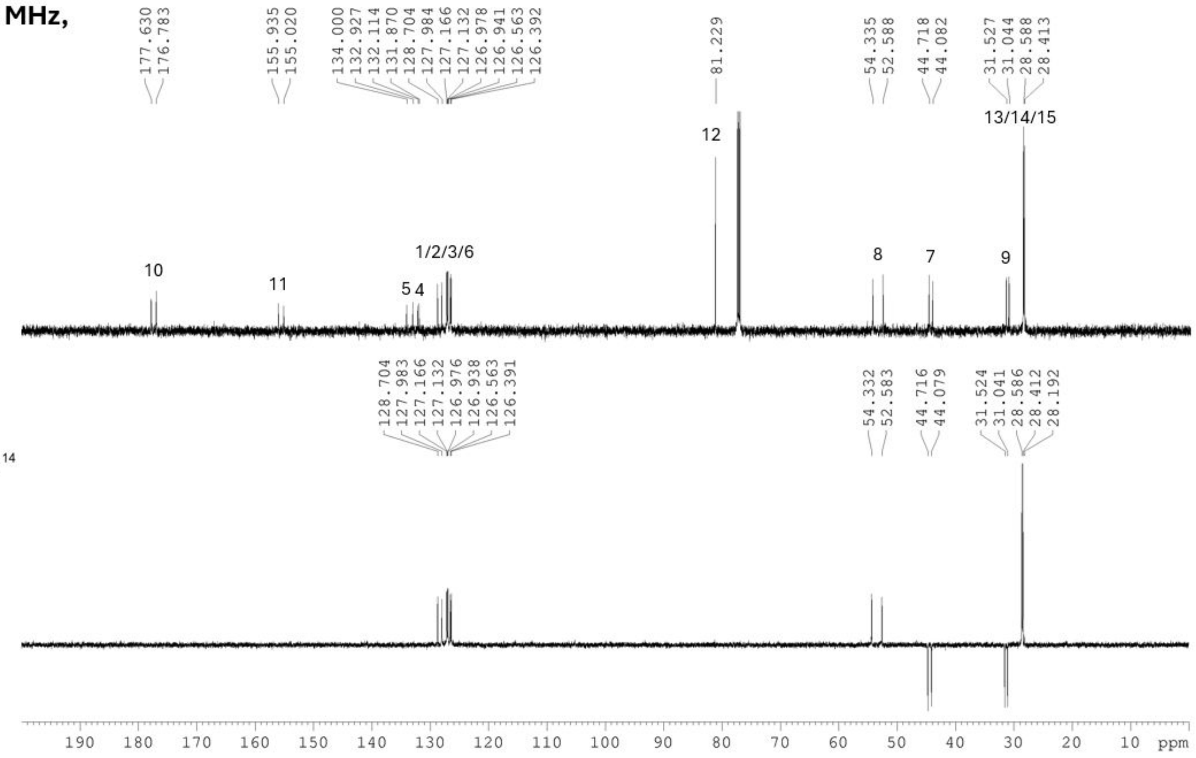

HRMS/MS spectrum of compound of 1a

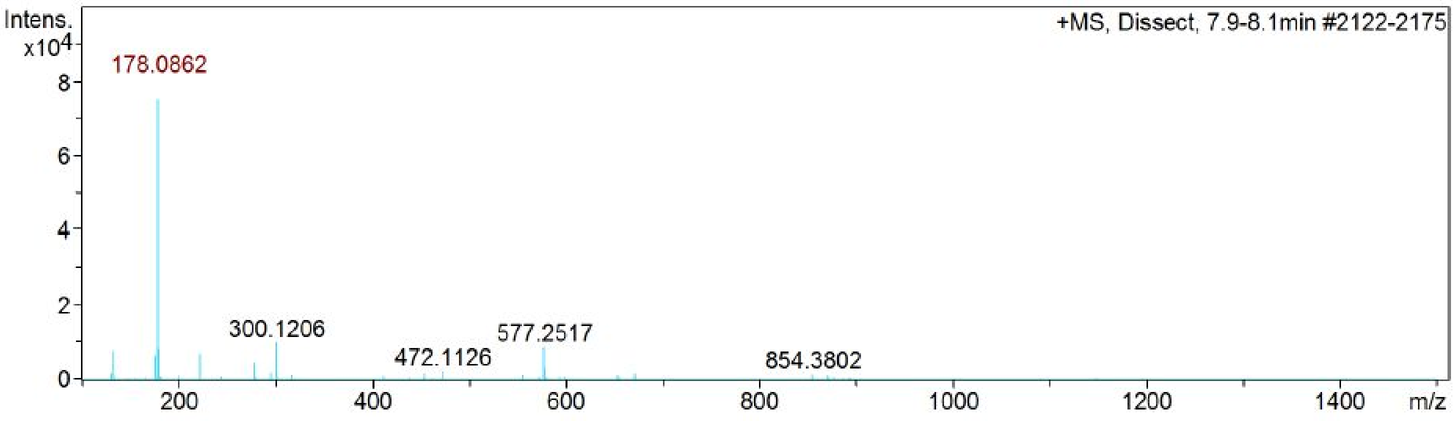

Meas. m/z	#	Ion Formula	Adduct	Sum Formula	Score	mSigma	m/z	err [mDa]	err [ppm]	rdb	e¯ Conf	N-Rule
178.0862	1	C10H12NO2	M+H	C10H11NO2	100.00	3.4	178.0863	0.1	0.4	5.5	even	ok

(*R*)-2-(tert-butoxycarbonyl)-1,2,3,4-tetrahydroisoquinoline-3-carboxylic acid (**1b**)

^{1}H NMR (600 MHz, acetone-d6)

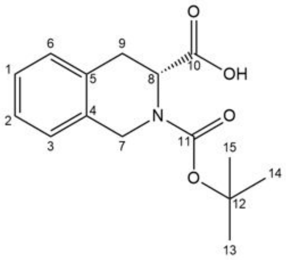

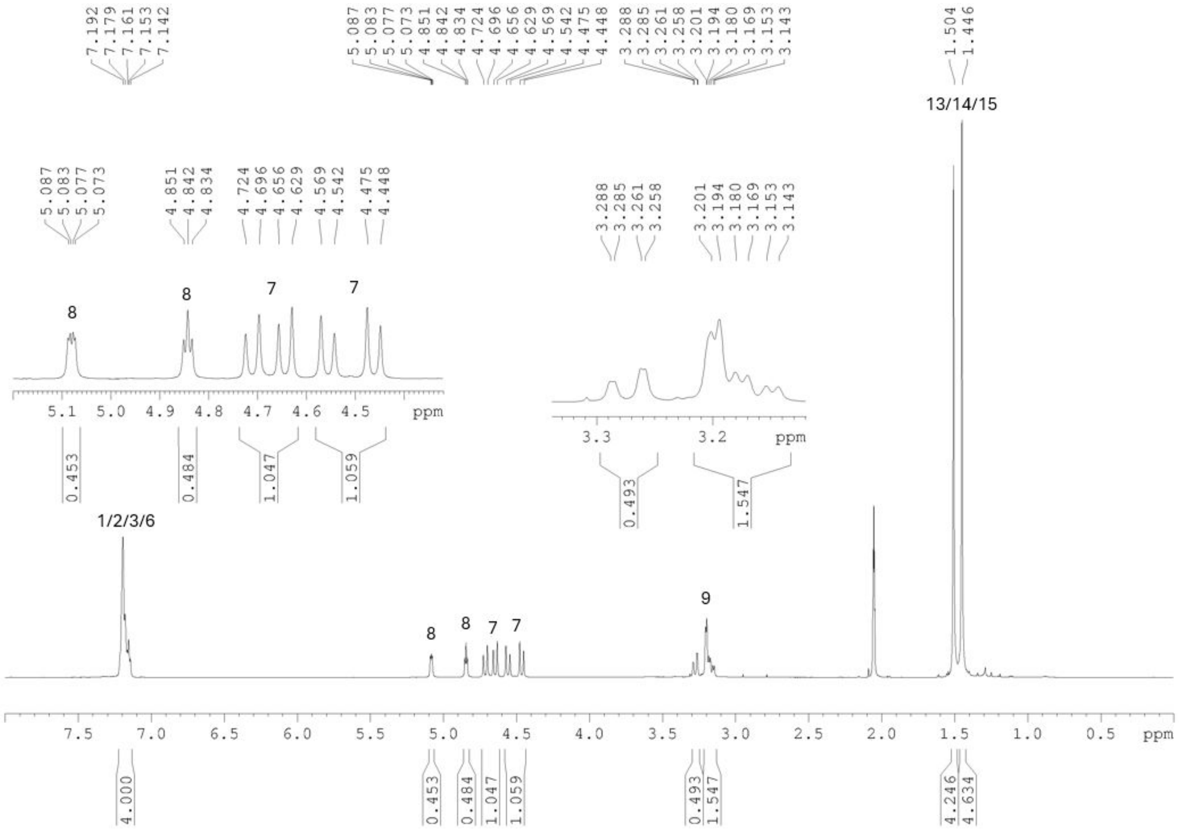

^{13}C NMR/DEPT135 (150 MHz, acetone-d6)

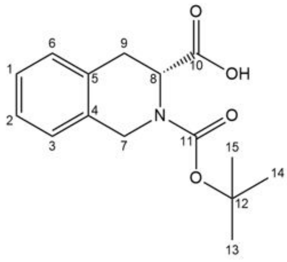

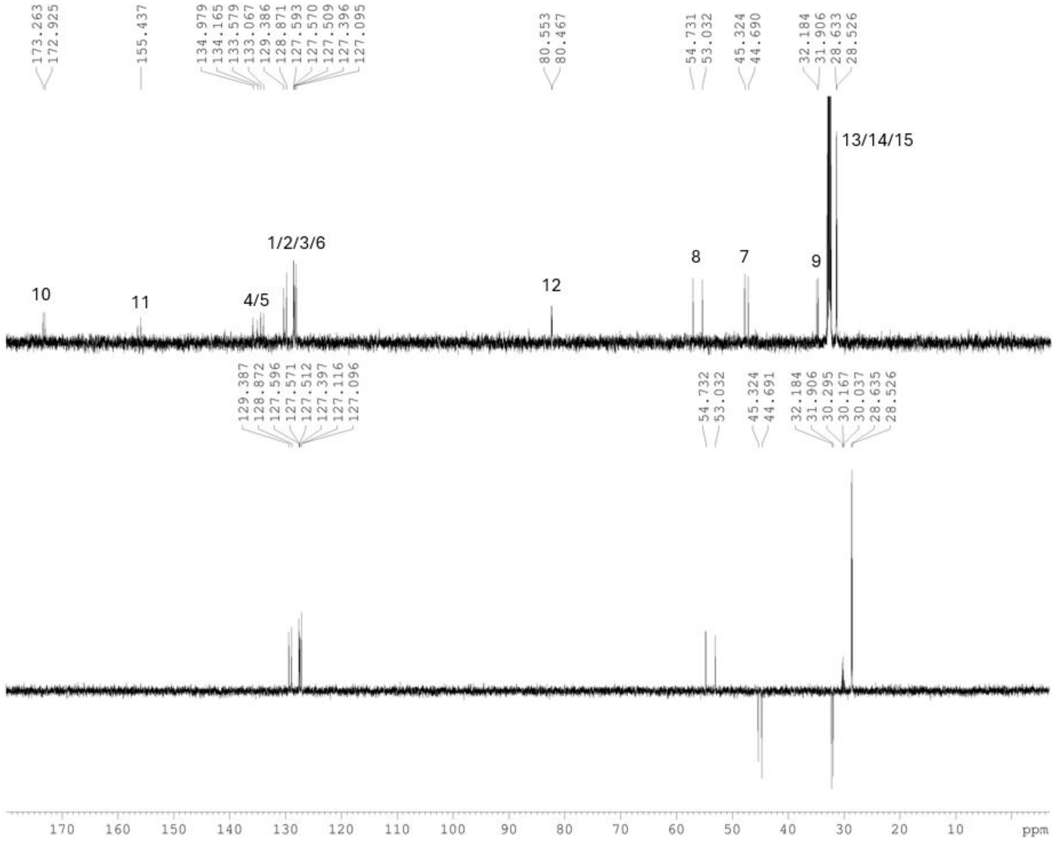

HRMS/MS spectrum of compound of 1b

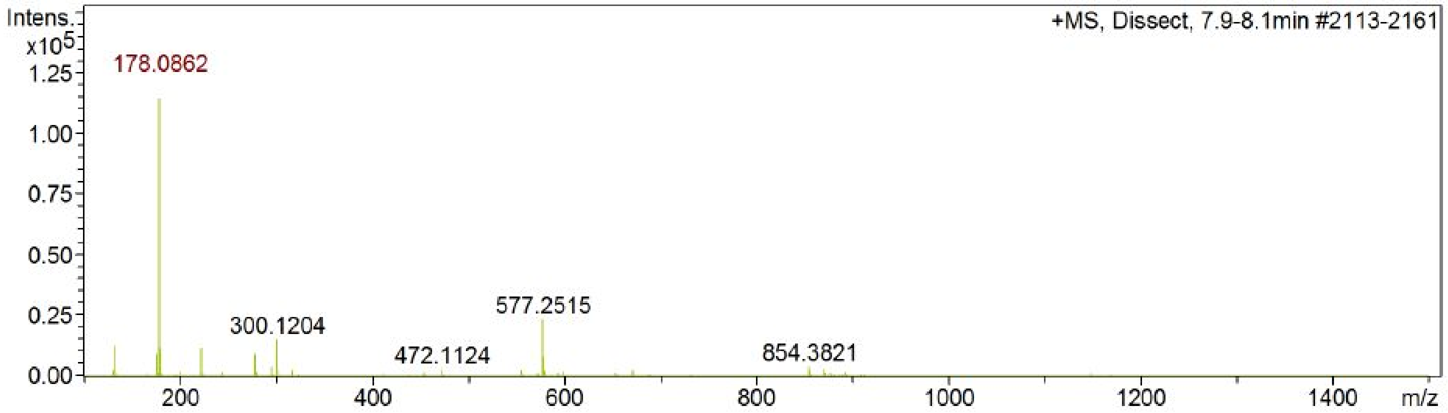

Meas. m/z	#	Ion Formula	Adduct	Sum Formula	Score	mSigma	m/z	err [mDa]	err [ppm]	rdb	e⁻ Conf	N-Rule
178.0862	1	C10H12NO2	M+H	C10H11NO2	100.00	8.2	178.0863	0.1	0.5	5.5	even	ok

Tert-butyl (*S*)-3-((furan-2-ylmethyl)carbamoyl)3,4-dihydroisoquinoline-2(1H)-carboxylate (**2a**)

^{1}H NMR (600 MHz, CDCl$_3$)

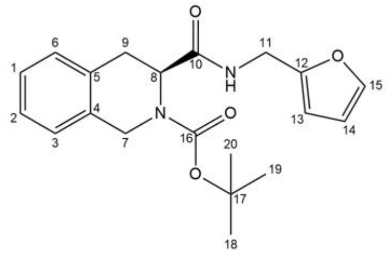

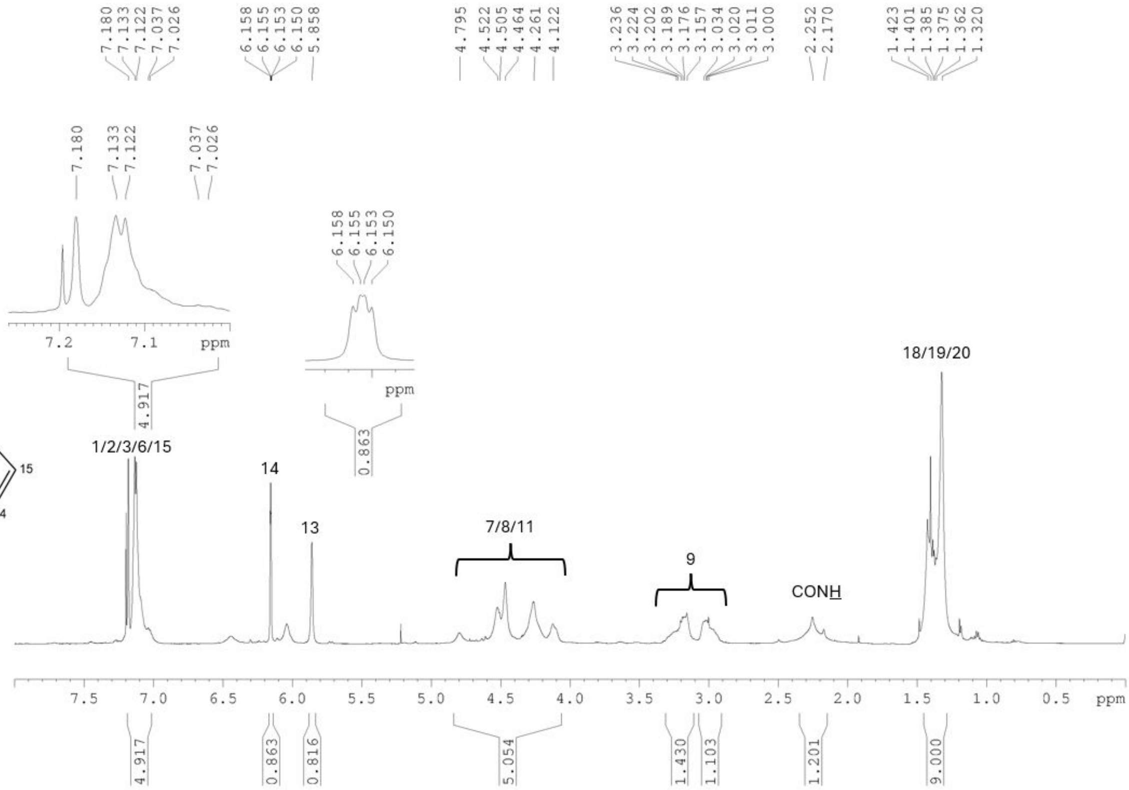

^{13}C NMR/DEPT135 (150 MHz, CDCl$_3$)

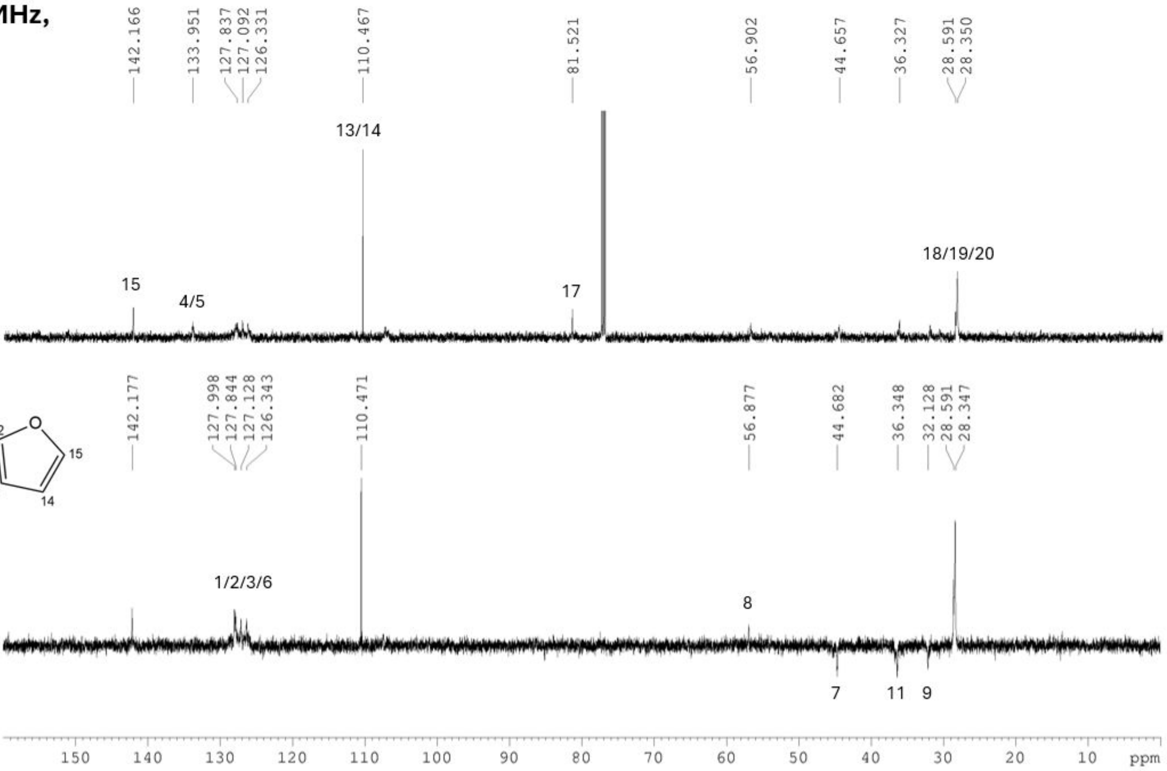

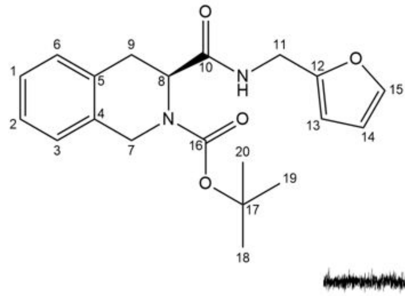

HRMS/MS spectrum of compound of 2a

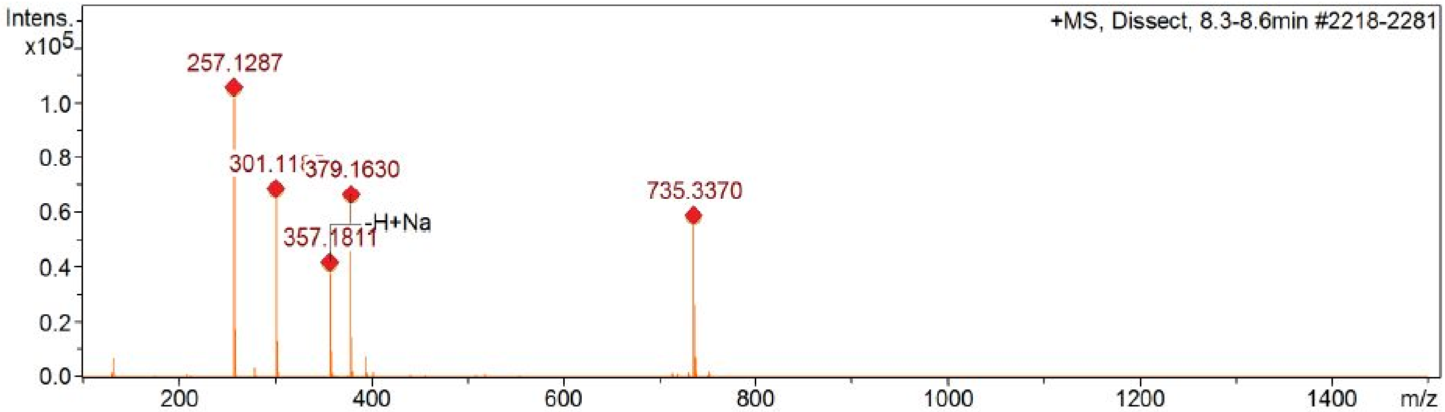

Meas. m/z	#	Ion Formula	Adduct	Sum Formula	Score	mSigma	m/z	err [mDa]	err [ppm]	rdb	e¯ Conf	N-Rule
257.1287	1	C15H17N2O2	M+H	C15H16N2O2	100.00	4.6	257.1285	-0.2	-0.8	8.5	even	ok

Tert-butyl (*R*)-3-((furan-2-ylmethyl)carbamoyl)3,4-dihydroisoquinoline-2(1H)-carboxylate (**2b**)

^{1}H NMR (600 MHz, $CDCl_3$)

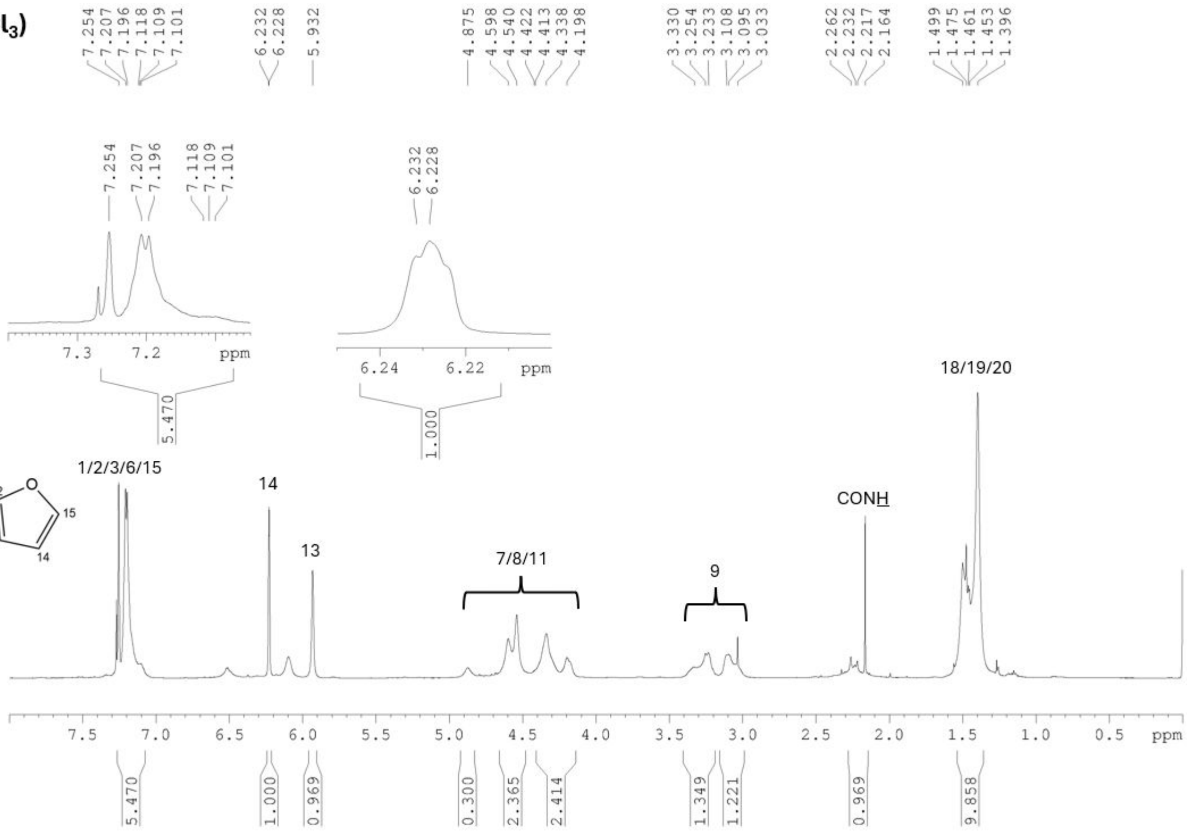

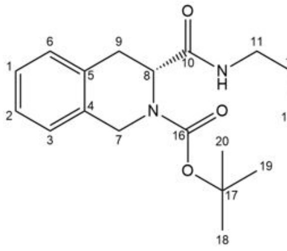

^{13}C NMR/DEPT135 (150 MHz, $CDCl_3$)

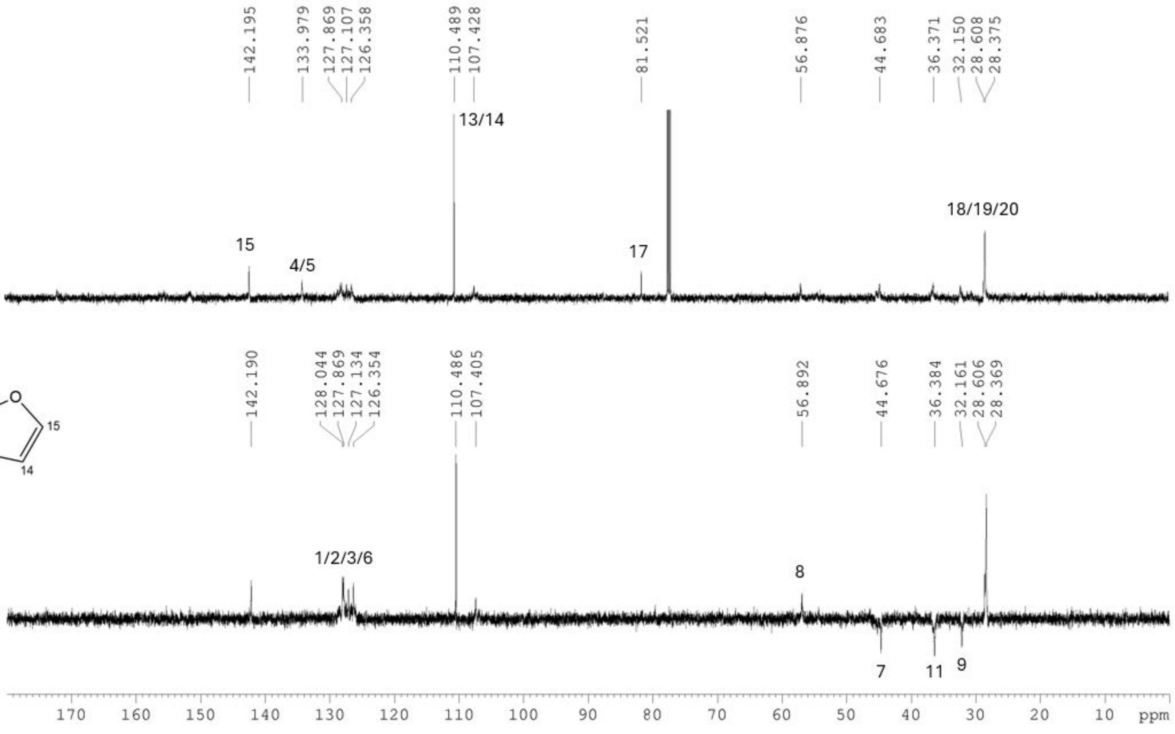

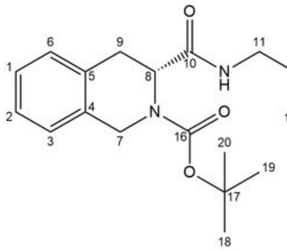

HRMS/MS spectrum of compound of 2b

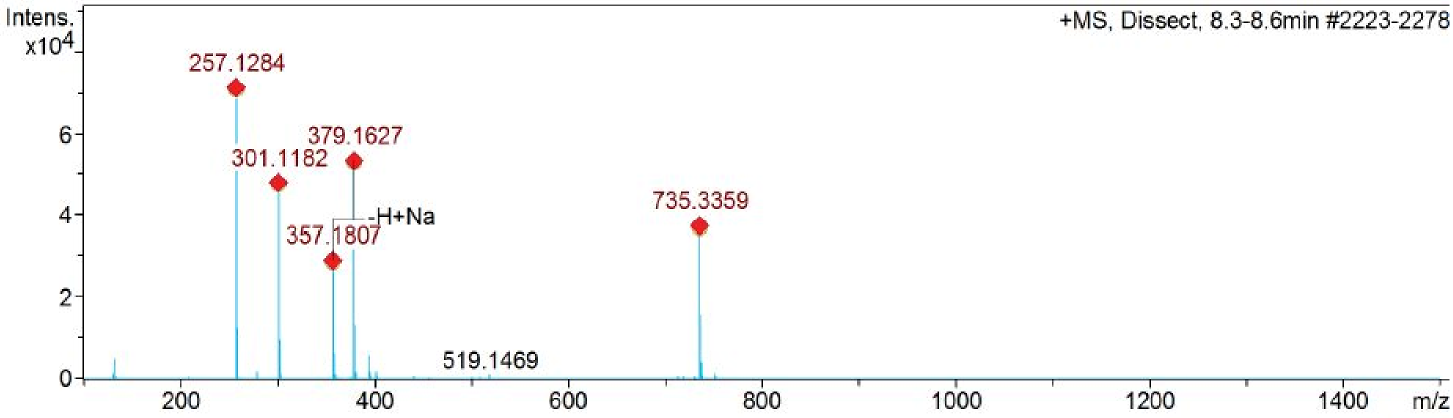

Meas. m/z	#	Ion Formula	Adduct	Sum Formula	Score	mSigma	m/z	err [mDa]	err [ppm]	rdb	e¯ Conf	N-Rule
257.1284	1	C15H17N2O2	M+H	C15H16N2O2	100.00	9.0	257.1285	0.0	0.2	8.5	even	ok

Tert-butyl (*S*)-3-(benzylcarbamoyl)-3,4-dihydroisoquinoline-2(1H)-carboxilate (**2c**)

^{1}H NMR (600 MHz, $CDCl_3$)

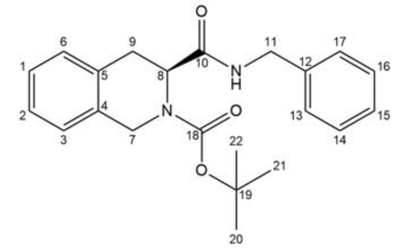

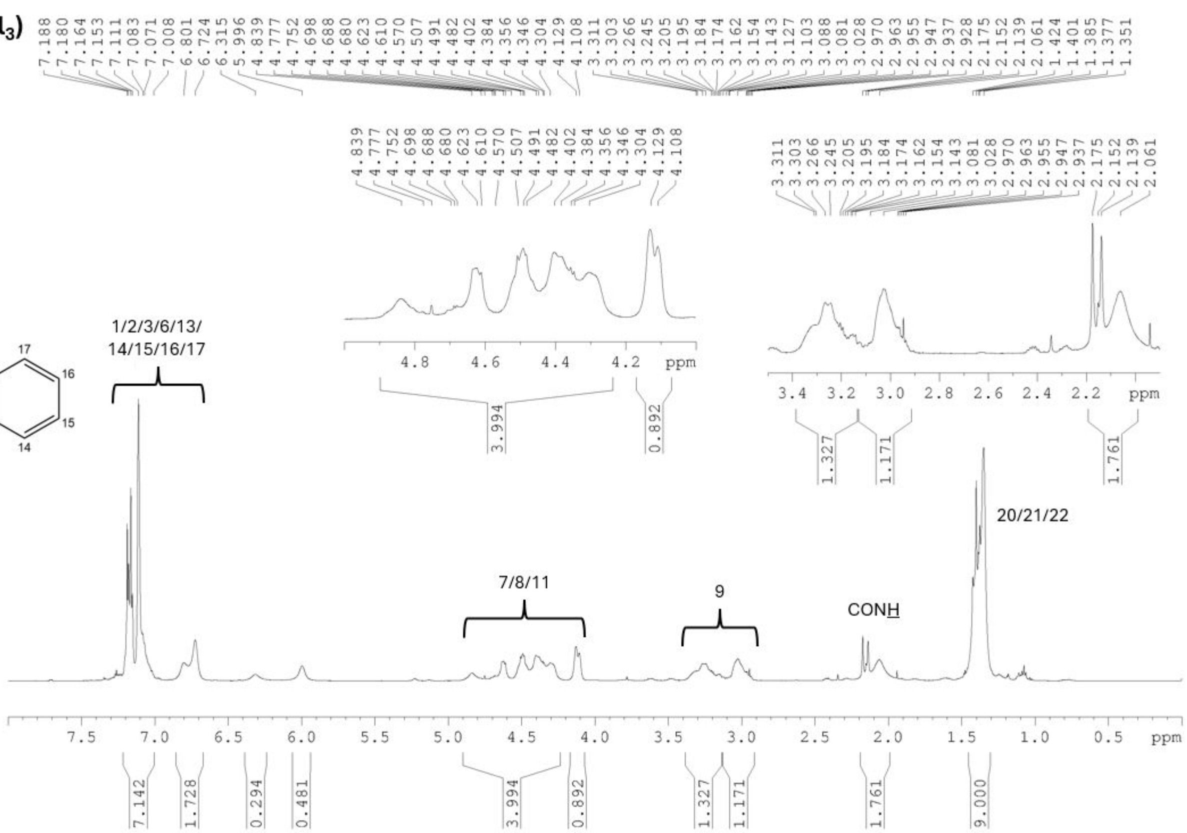

^{13}C NMR/DEPT135 (150 MHz, $CDCl_3$)

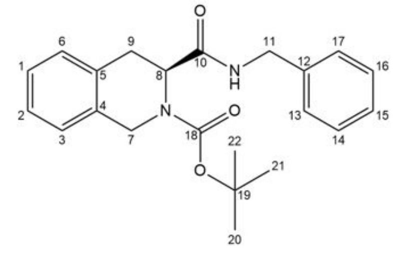

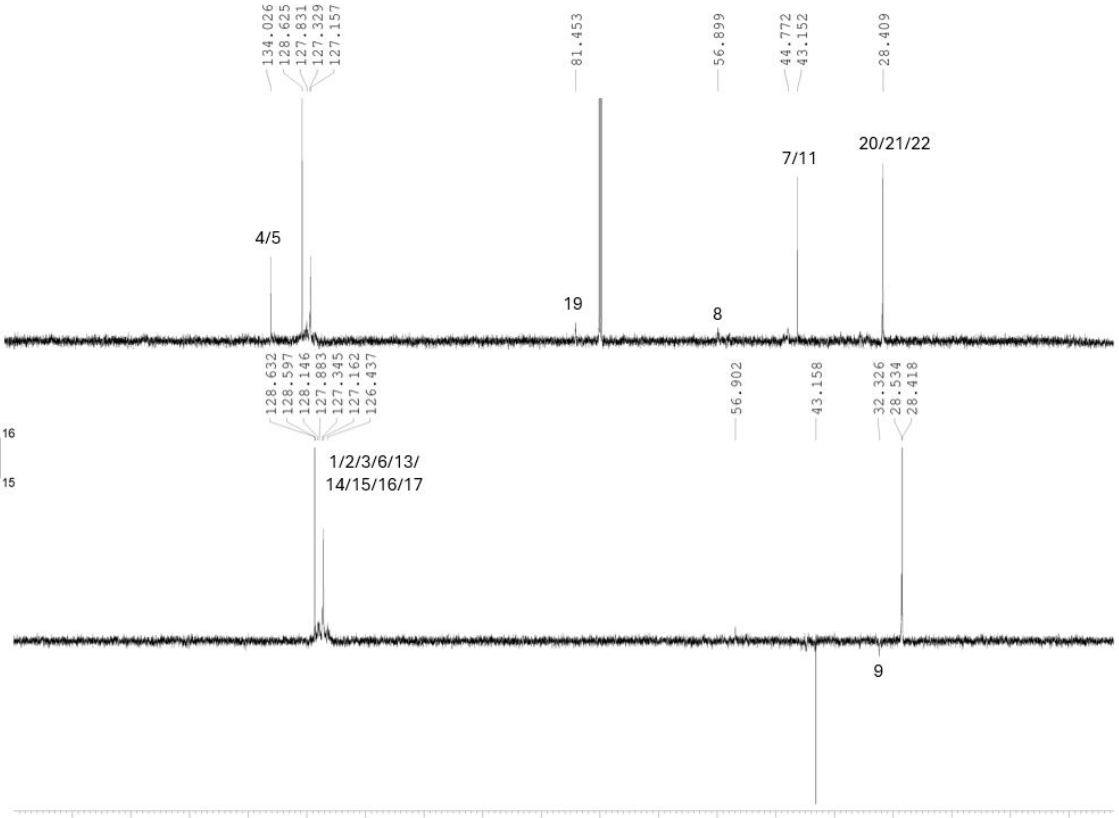

HRMS/MS spectrum of compound of 2c

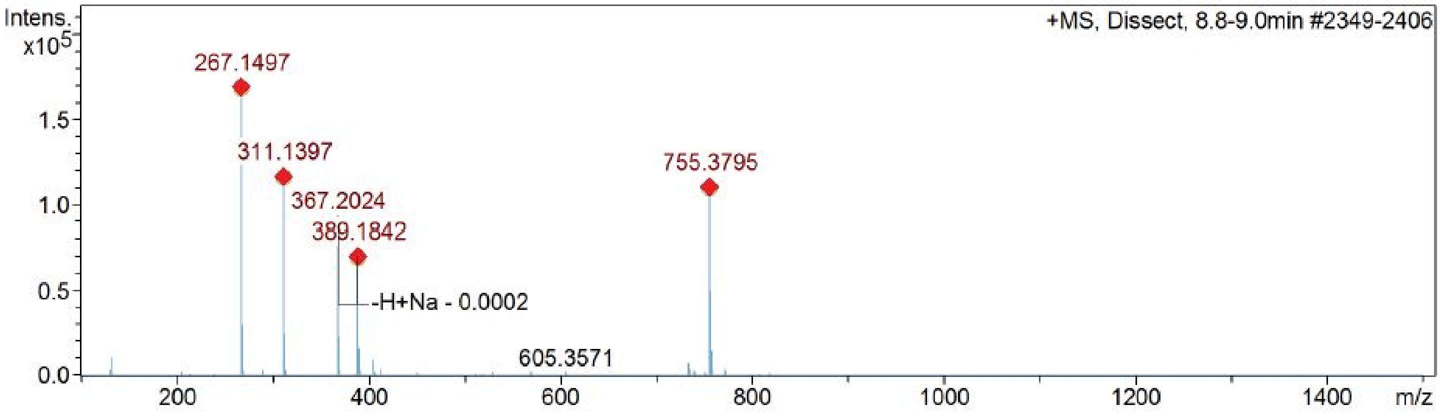

Meas. m/z	#	Ion Formula	Adduct	Sum Formula	Score	mSigma	m/z	err [mDa]	err [ppm]	rdb	e⁻ Conf	N-Rule
267.1497	1	C17H19N2O	M+H	C17H18N2O	100.00	7.4	267.1492	-0.5	-2.1	9.5	even	ok

Tert-butyl (*S*)-3-((3-phenylpropyl)carbamoyl)-3,4-dihydroisoquinoline-2(1H)-carboxilate (**2d**)

^{1}H NMR (600 MHz, acetone-d6)

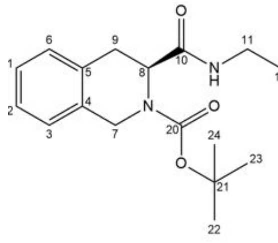

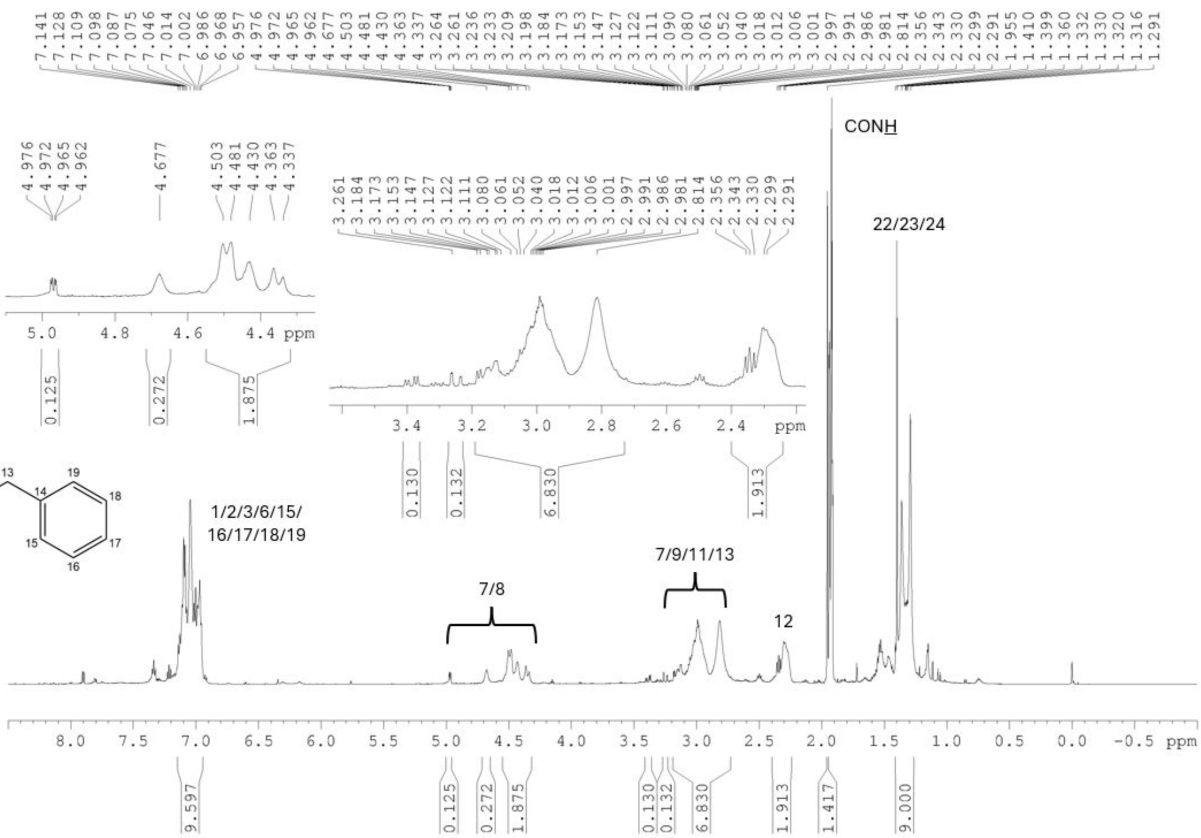

^{13}C NMR/DEPT (150 MHz, acetone-d6)

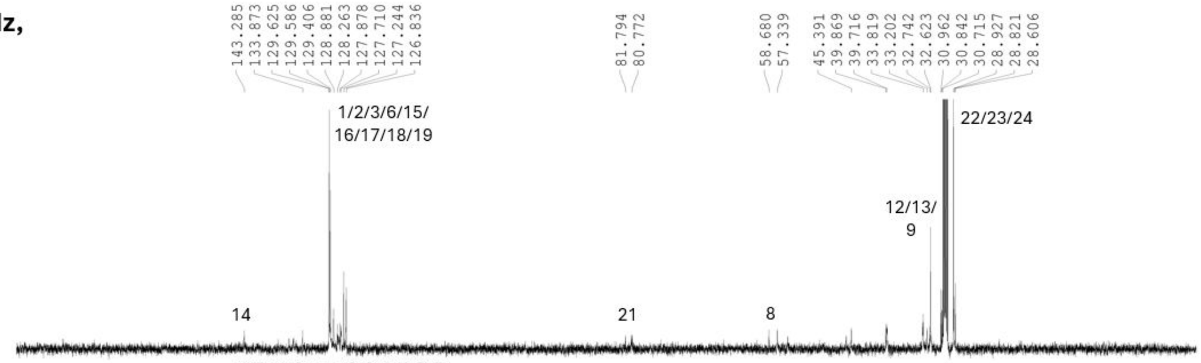

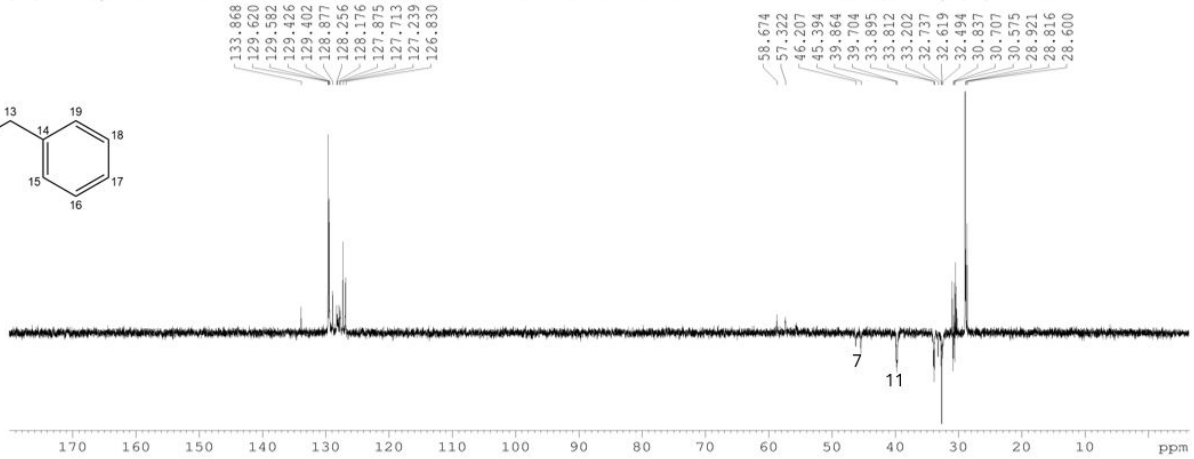

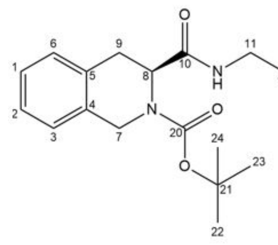

HRMS/MS spectrum of compound of 2d

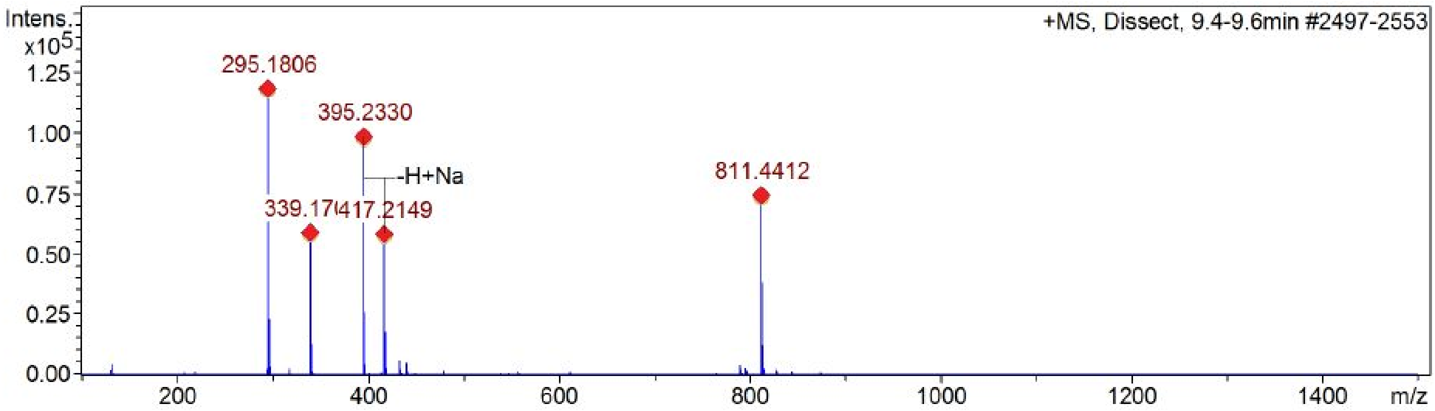

Meas. m/z	#	Ion Formula	Adduct	Sum Formula	Score	mSigma	m/z	err [mDa]	err [ppm]	rdb	e⁻ Conf	N-Rule
295.1806	1	C19H23N2O	M+H	C19H22N2O	100.00	7.5	295.1805	-0.1	-0.4	9.5	even	ok

Tert-butyl (*S*)-3-(4-methylpiperazine-1-carbonyl)-3,4-dihydroisoquinoline-2(1H) carboxylate (**2e**)

^{1}H NMR (600 MHz, $CDCl_3$)

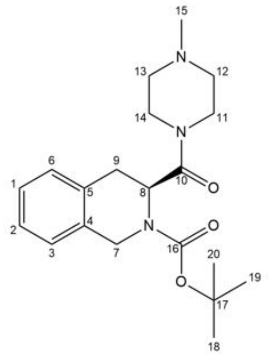

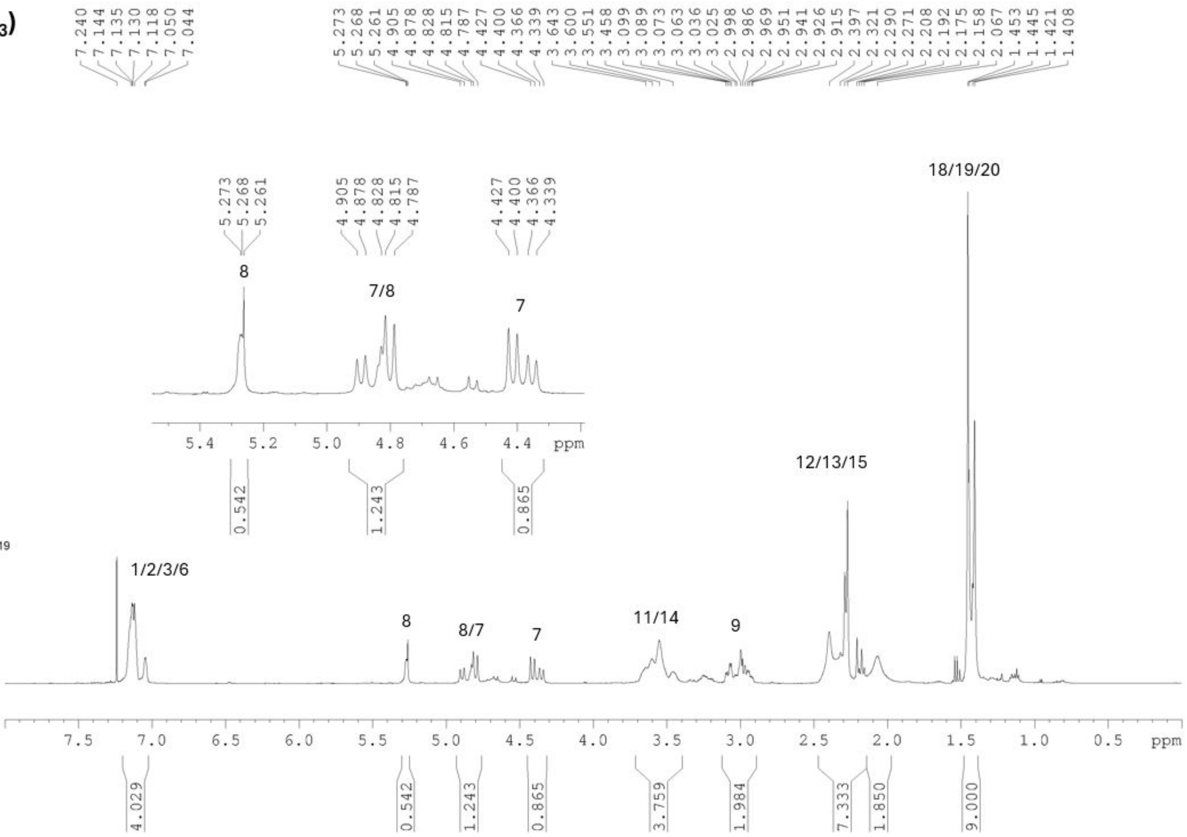

^{13}C NMR/DEPT135 (150 MHz, $CDCl_3$)

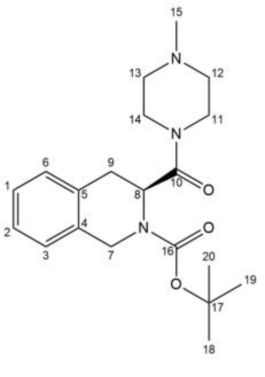

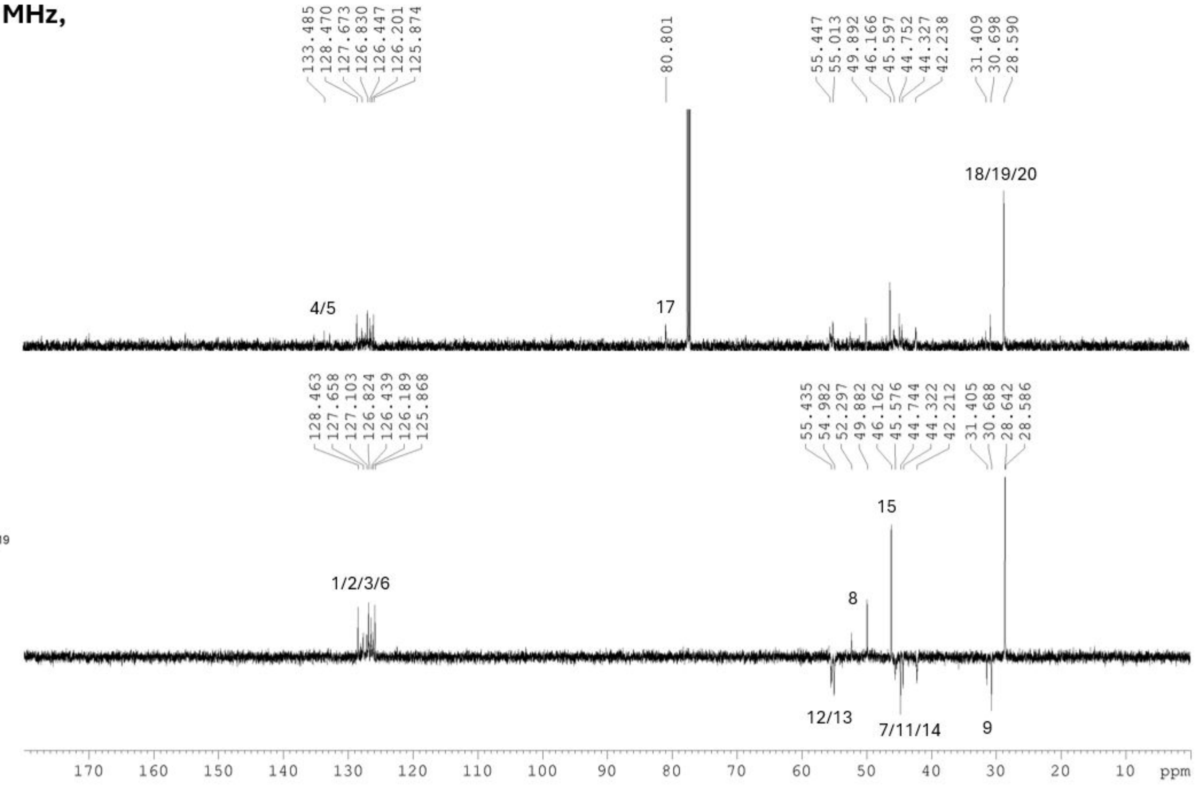

HRMS/MS spectrum of compound of 2e

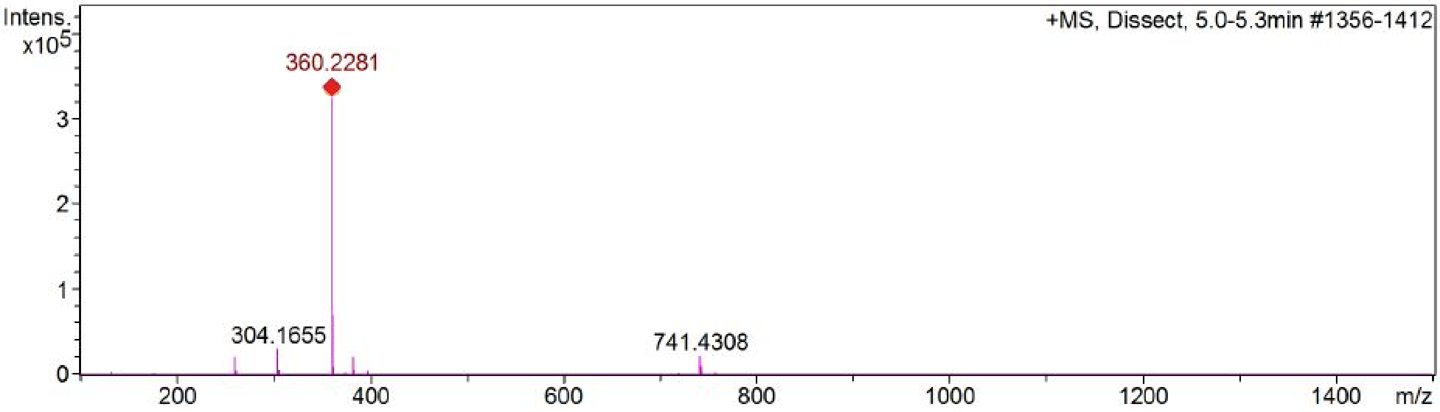

Meas. m/z	#	Ion Formula	Adduct	Sum Formula	Score	mSigma	m/z	err [mDa]	err [ppm]	rdb	e⁻ Conf	N-Rule
360.2281	1	C20H30N3O3	M+H	C20H29N3O3	100.00	8.0	360.2282	0.1	0.2	7.5	even	ok

Tert-butyl (*S*)-3-((3-morpholinopropyl)carbamoyl)-3,4-dihydroisoquinoline-2(1H) carboxylate (**2f**)

^{1}H NMR (600 MHz, acetone-d6)

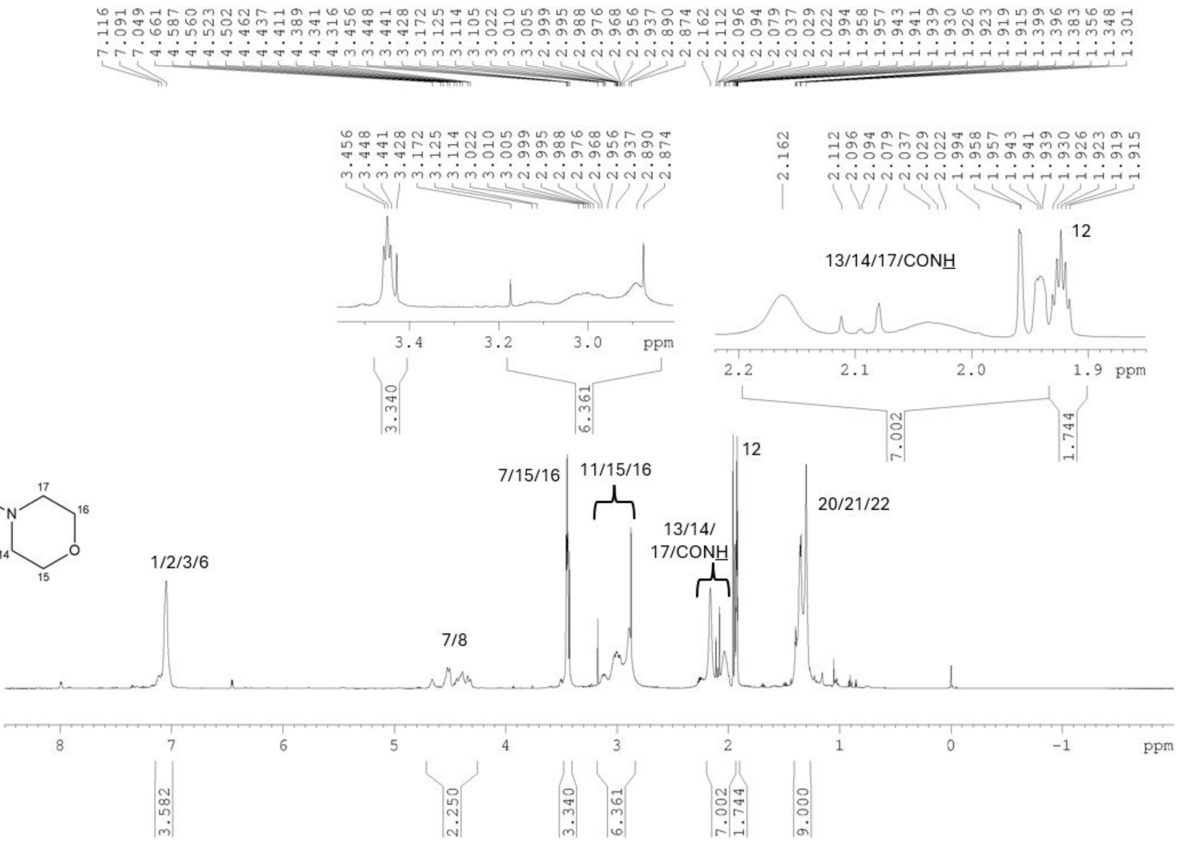

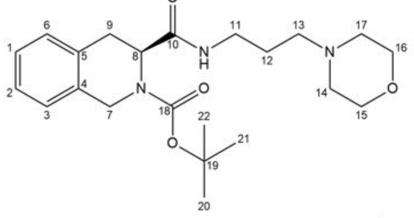

^{13}C NMR/DEPT135 (150 MHz, acetone-d6)

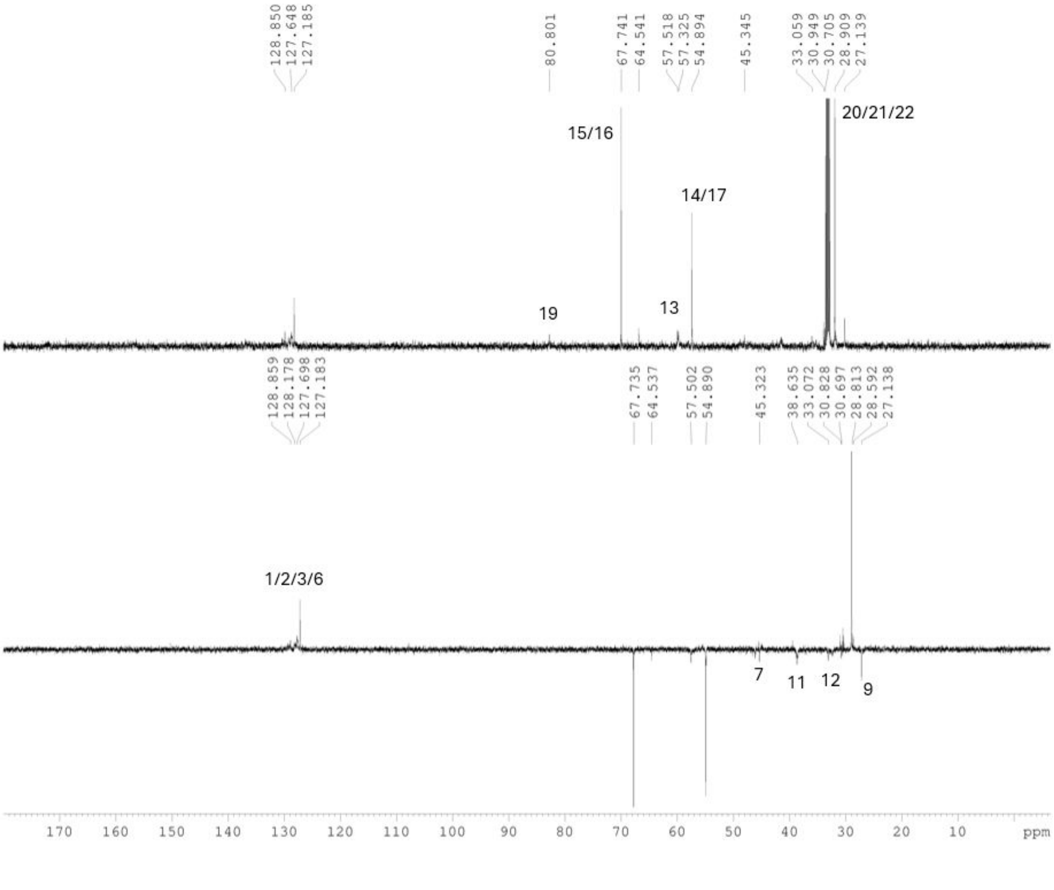

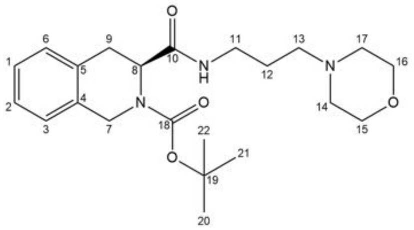

HRMS/MS spectrum of compound of 2f

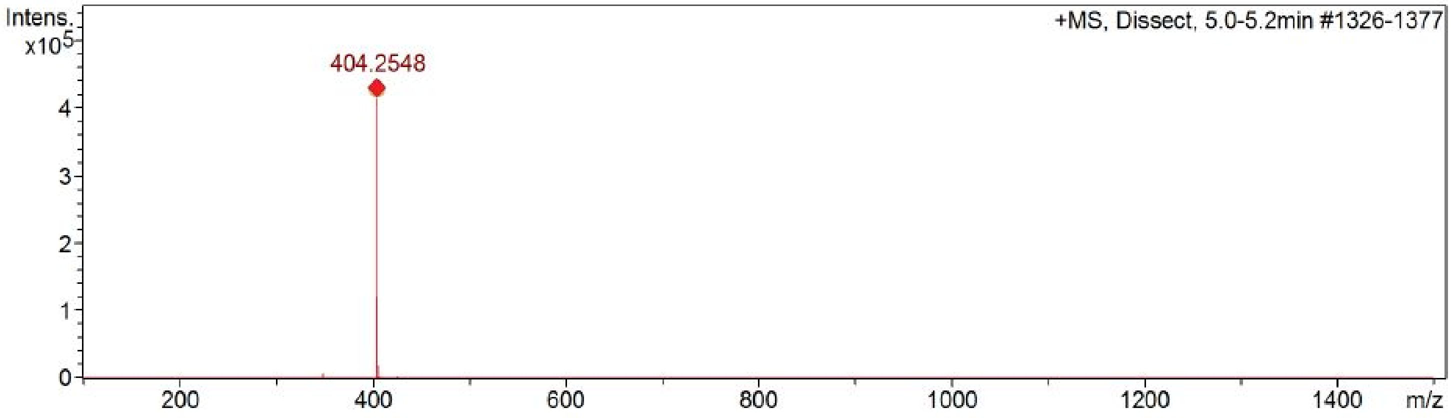

Meas. m/z	#	Ion Formula	Adduct	Sum Formula	Score	mSigma	m/z	err [mDa]	err [ppm]	rdb	e⁻ Conf	N-Rule
404.2548	1	C22H34N3O4	M+H	C22H33N3O4	100.00	20.2	404.2544	-0.4	-1.0	7.5	even	ok

(*S*)-*N*-(furan-2-ylmethyl)-1,2,3,4-tetrahydroisoquinoline-3-carboxamide (**3a**)

^{1}H NMR (600 MHz, acetone-d6)

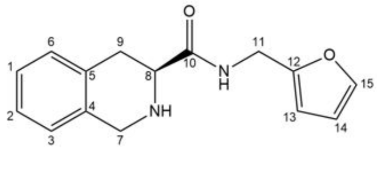

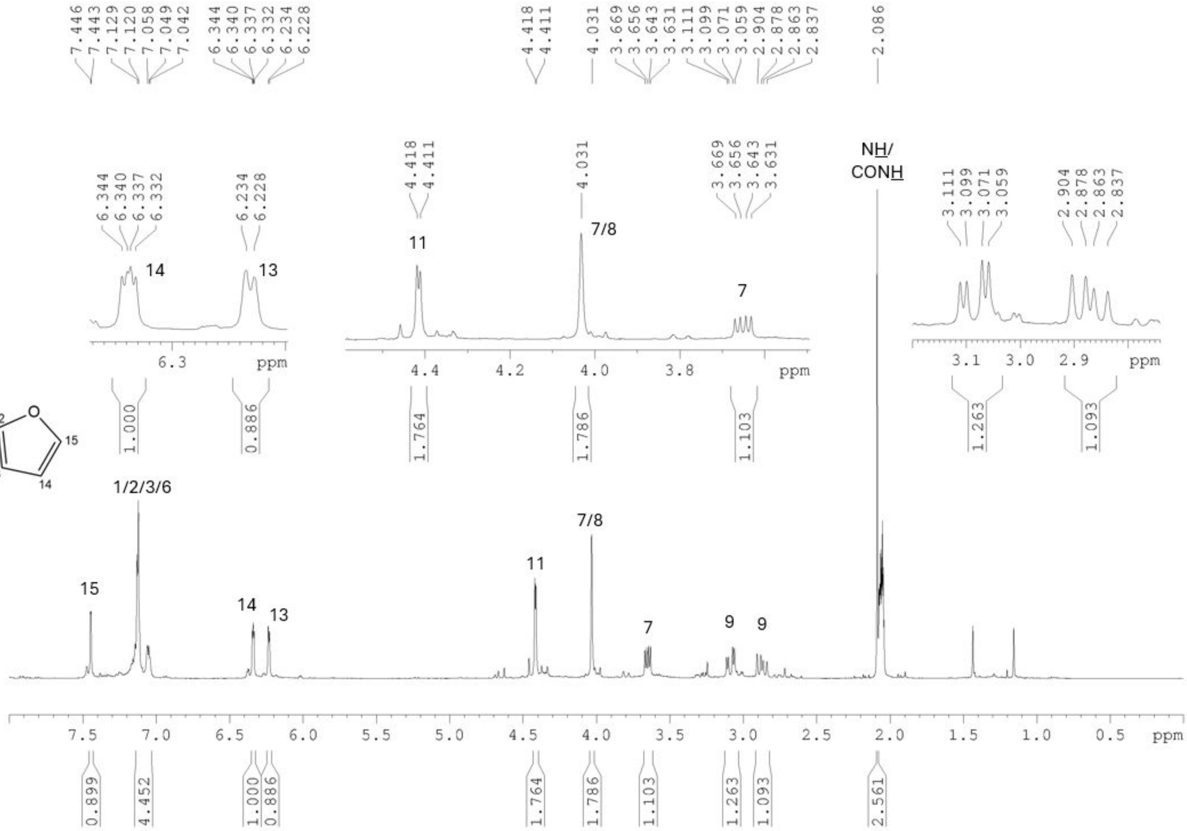

^{13}C NMR/DEPT135 (150 MHz, acetone-d6)

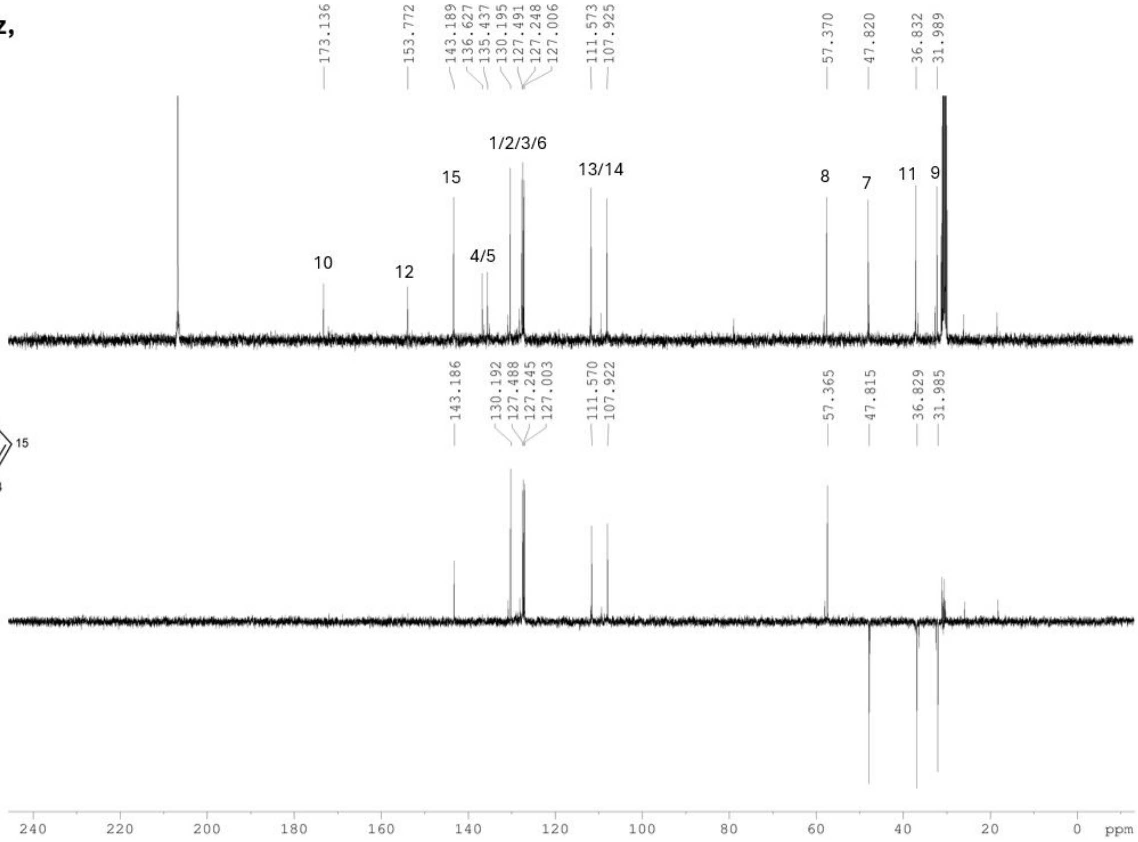

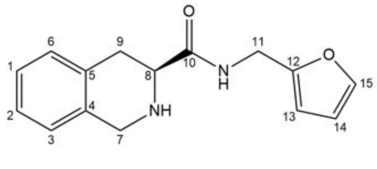

HRMS/MS spectrum of compound of 3a

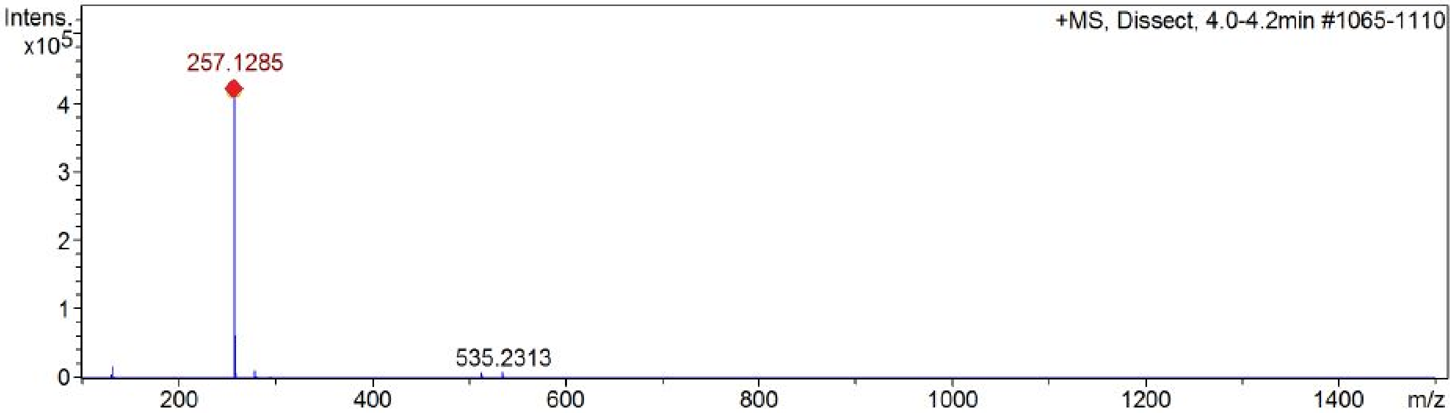

Meas. m/z	#	Ion Formula	Adduct	Sum Formula	Score	mSigma	m/z	err [mDa]	err [ppm]	rdb	e⁻ Conf	N-Rule
257.1285	1	C15H17N2O2	M+H	C15H16N2O2	100.00	13.1	257.1285	-0.1	-0.3	8.5	even	ok

(*R*)-*N*-(furan-2-ylmethyl)-1,2,3,4-tetrahydroisoquinoline-3-carboxamide (**3b**)

¹H NMR (600 MHz, acetone-d6)

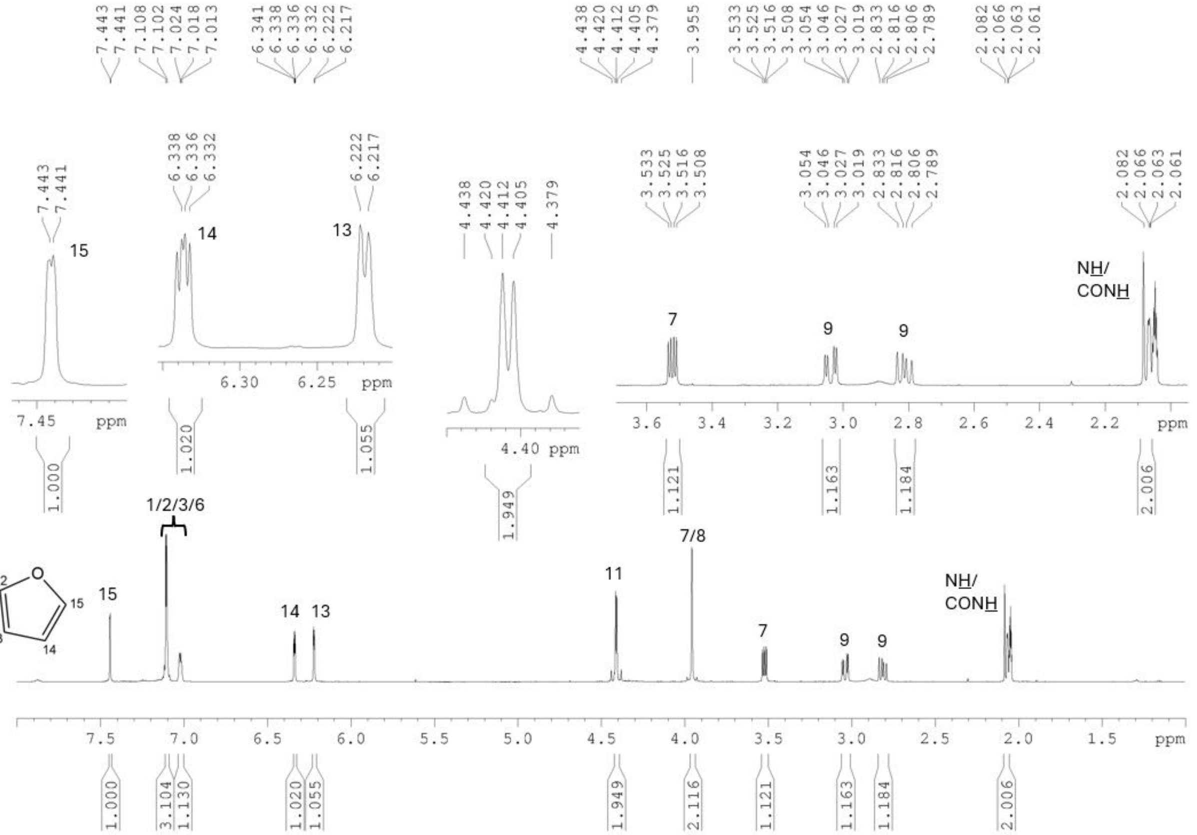

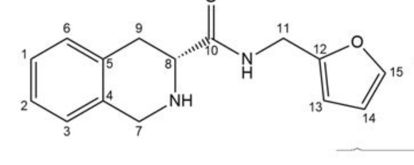

¹³C NMR/DEPT135 (150 MHz, acetone-d6)

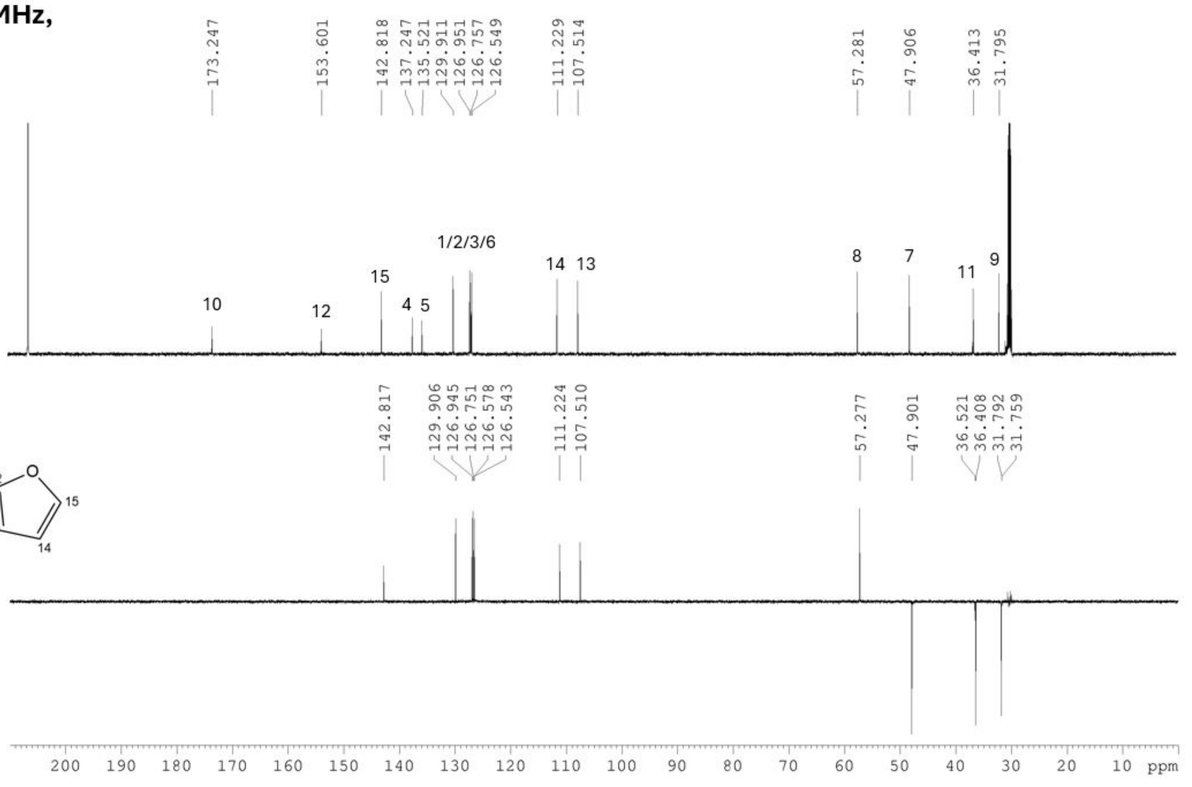

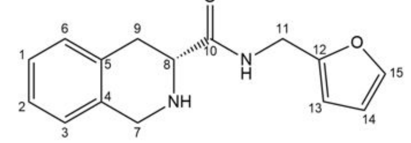

HRMS/MS spectrum of compound of 3b

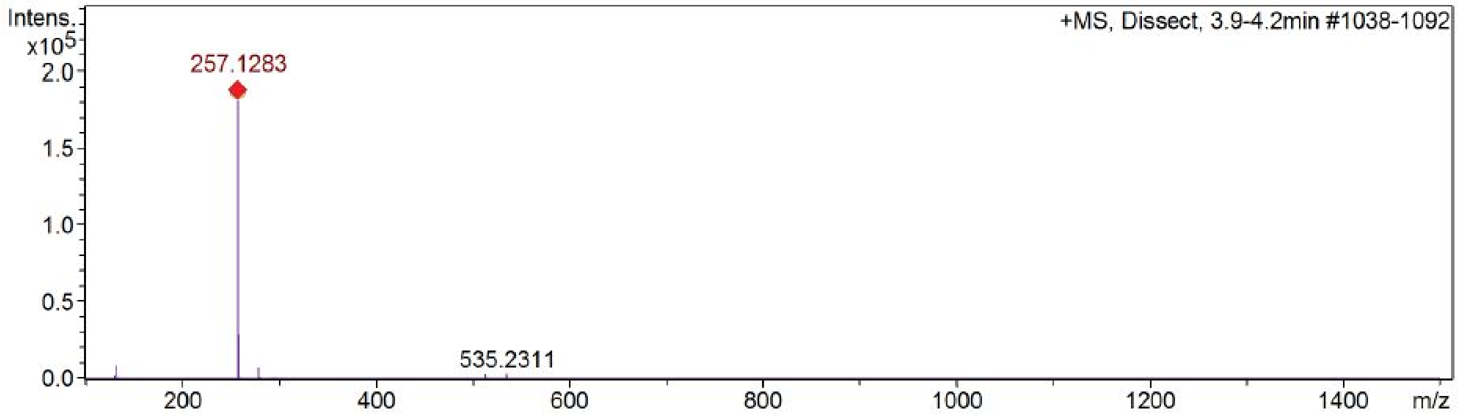

Meas. m/z	#	Ion Formula	Adduct	Sum Formula	Score	mSigma	m/z	err [mDa]	err [ppm]	rdb	e⁻ Conf	N-Rule
257.1283	1	C15H17N2O2	M+H	C15H16N2O2	100.00	11.6	257.1285	0.2	0.7	8.5	even	ok

(*S*)-*N*-benzyl-1,2,3,4-tetrahydroisoquinoline-3-carboxamide (**3c**)

^{1}H NMR (600 MHz, acetone-d6)

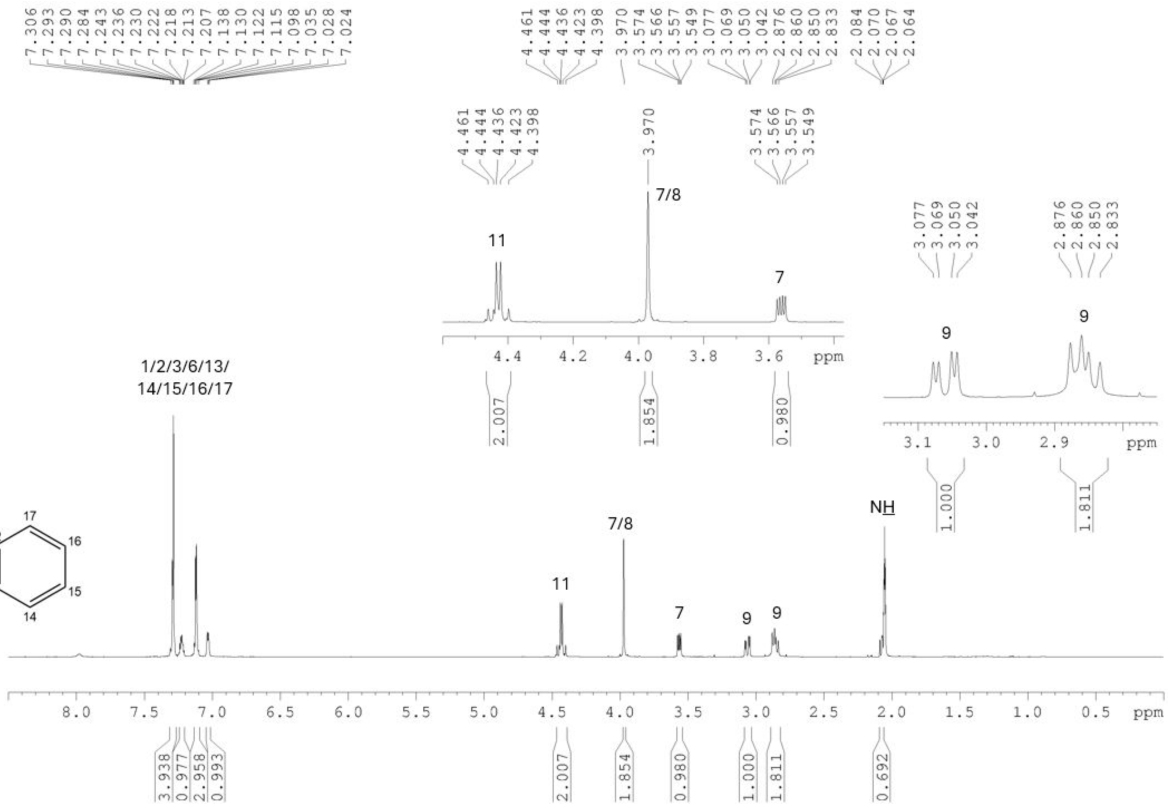

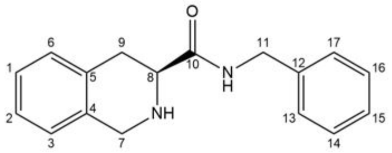

^{13}C NMR/DEPT135 (150 MHz, acetone-d6)

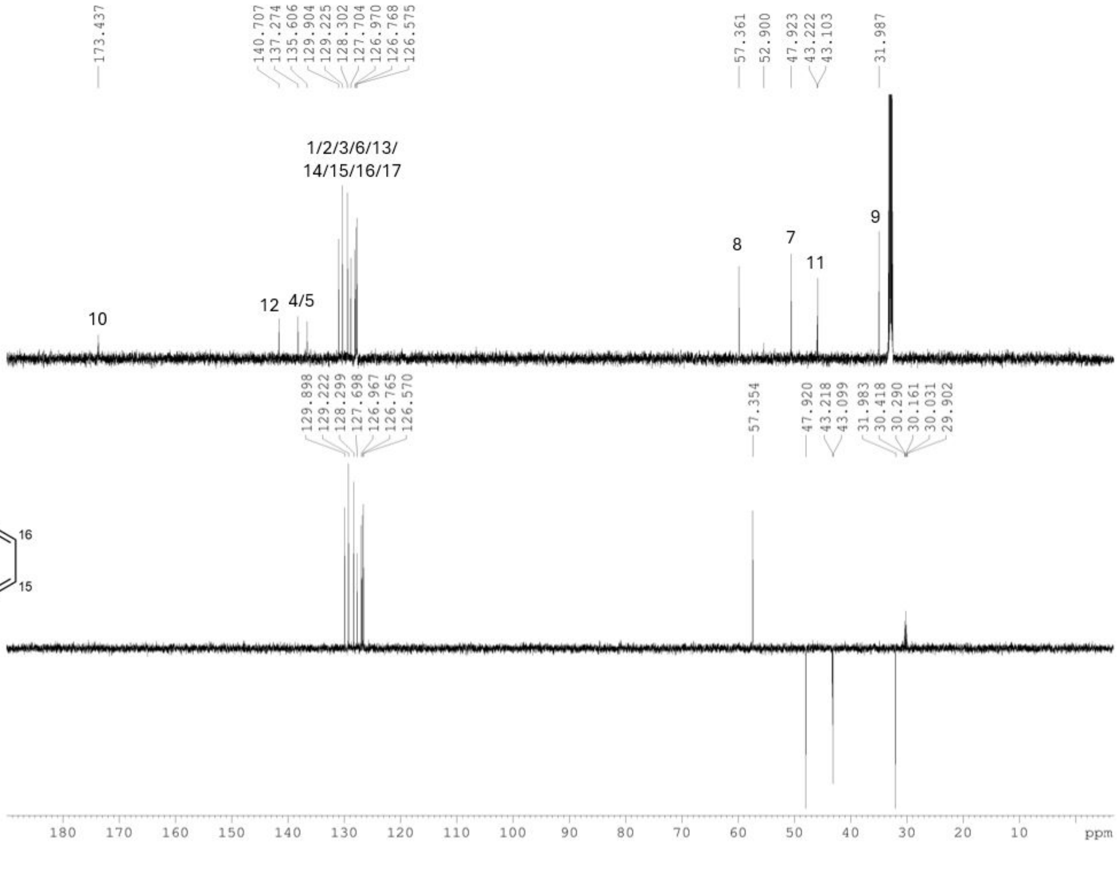

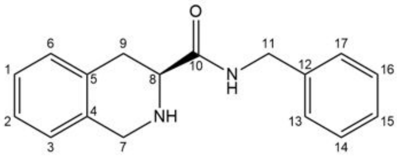

HRMS/MS spectrum of compound of 3c

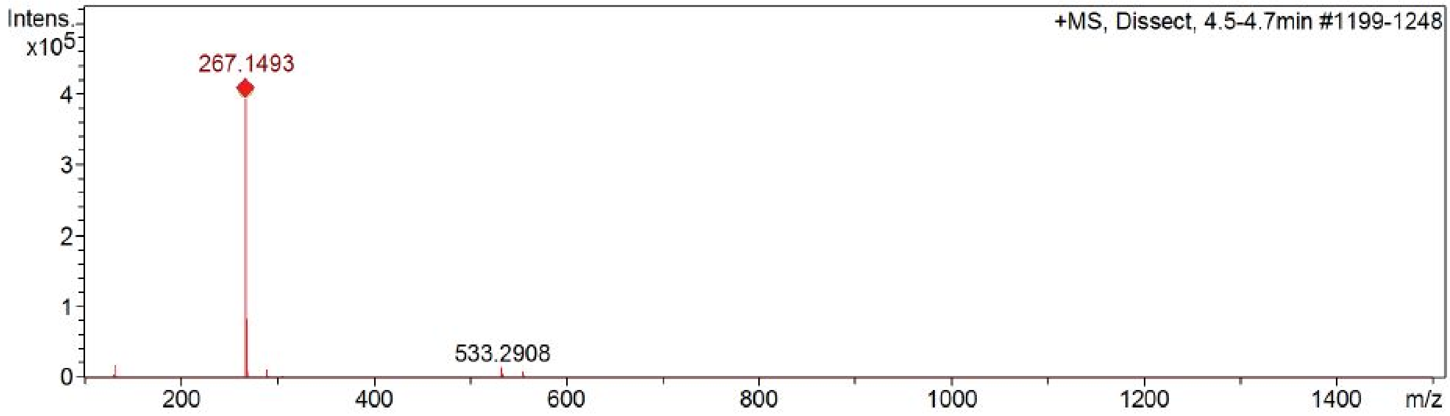

Meas. m/z	#	Ion Formula	Adduct	Sum Formula	Score	mSigma	m/z	err [mDa]	err [ppm]	rdb	e⁻ Conf	N-Rule
267.1493	1	C17H19N2O	M+H	C17H18N2O	100.00	10.1	267.1492	-0.1	-0.5	9.5	even	ok

(*S*)-*N*-(3-phenylpropyl)-1,2,3,4-tetrahydroisoquinoline-3-carboxamide (**3d**)

^{1}H NMR (400 MHz, acetone-d6)

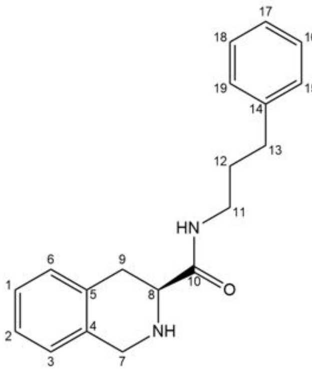

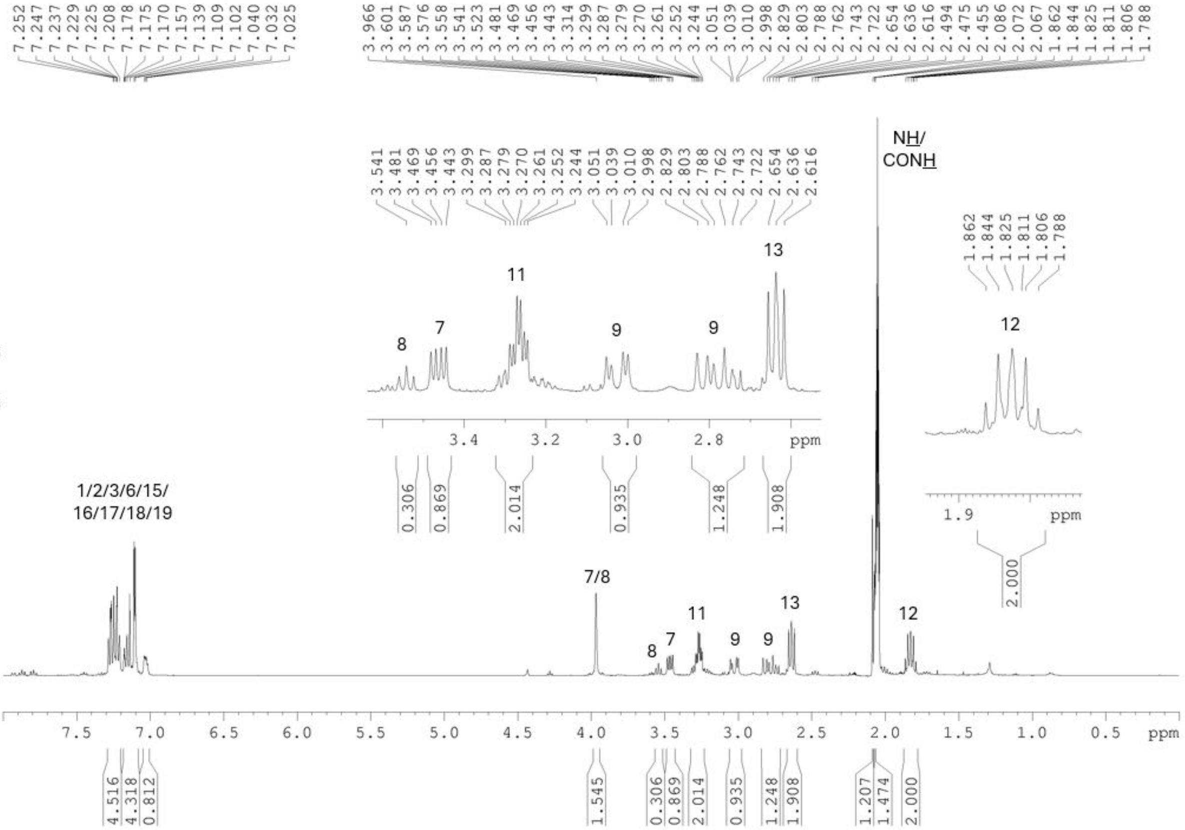

^{13}C NMR/DEPT (150 MHz, acetone-d6)

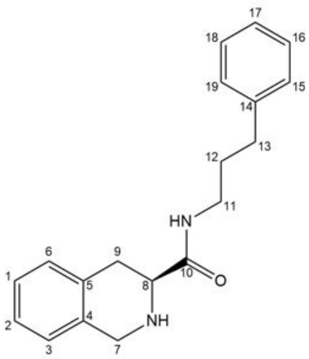

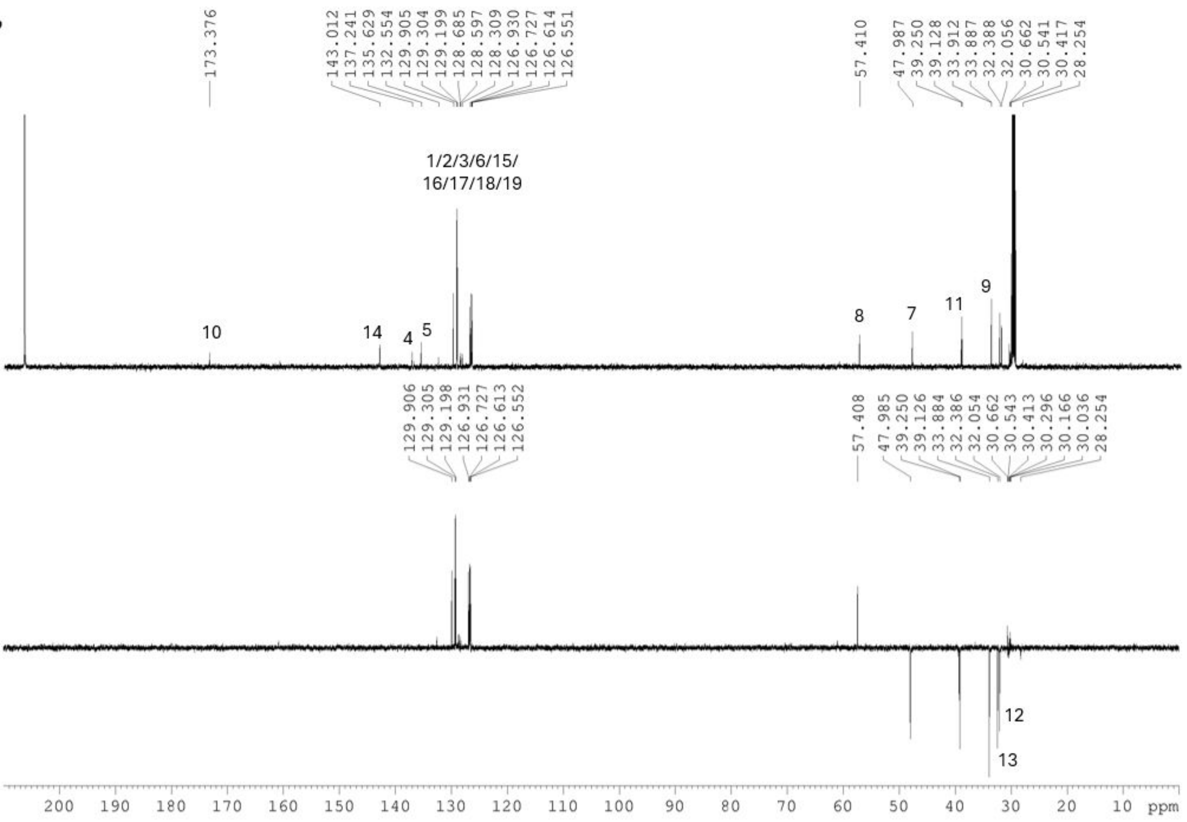

COSY

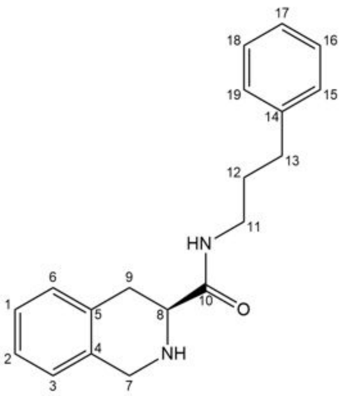
17
18
16
19
15
14
12
13
HN
11
6
9
1
5
8
10
O
2
4
NH
3
7

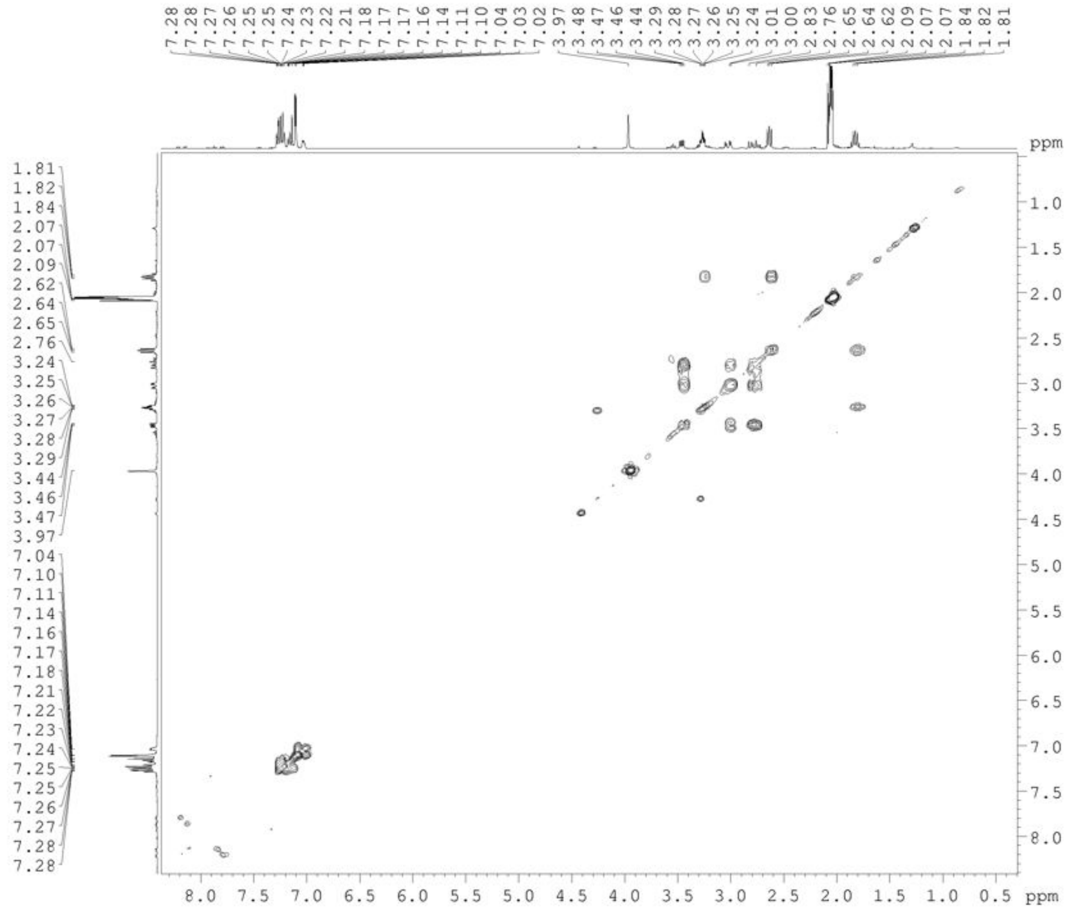
7.28 7.28 7.27 7.26 7.25 7.25 7.24 7.23 7.22 7.21 7.18 7.17 7.17 7.16 7.14 7.11 7.10 7.04 7.03 7.02 3.97 3.48 3.47 3.46 3.44 3.29 3.28 3.27 3.26 3.25 3.24 3.01 3.00 2.83 2.76 2.65 2.64 2.62 2.09 2.07 2.07 1.84 1.82 1.81
1.81 1.82 1.84 2.07 2.07 2.09 2.62 2.64 2.65 2.76 3.24 3.25 3.26 3.27 3.28 3.29 3.44 3.46 3.47 3.97 7.04 7.10 7.11 7.14 7.16 7.17 7.18 7.21 7.22 7.23 7.24 7.25 7.25 7.26 7.27 7.28 7.28
ppm

HSQC

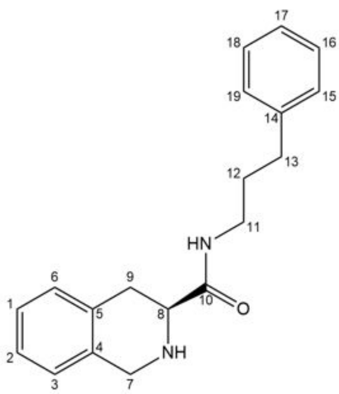
17
18
16
19
15
14
12
13
HN
11
6
9
1
5
8
10
O
2
4
NH
3
7

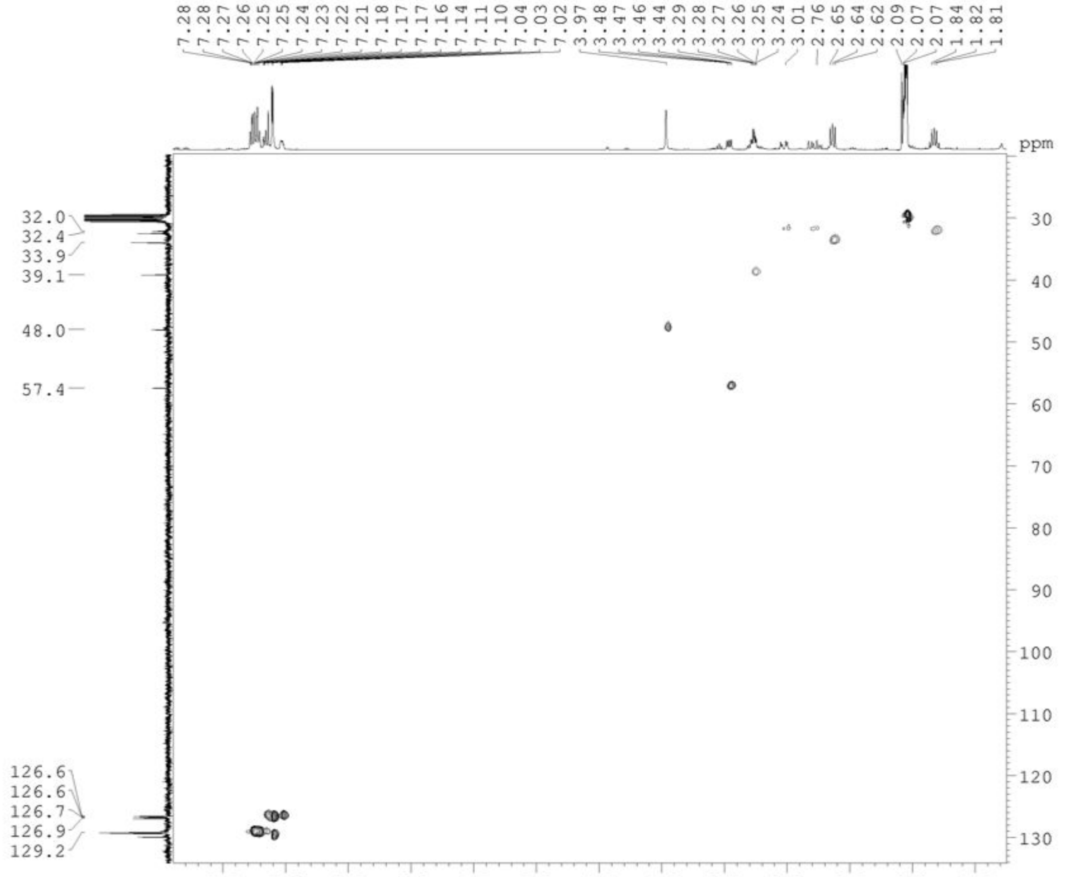
7.28 7.28 7.27 7.26 7.25 7.25 7.24 7.23 7.22 7.21 7.18 7.17 7.17 7.16 7.14 7.11 7.10 7.04 7.03 7.02 3.97 3.48 3.47 3.46 3.44 3.29 3.28 3.27 3.26 3.25 3.24 3.01 2.76 2.65 2.64 2.62 2.09 2.07 2.07 1.84 1.82 1.81
32.0 32.4 33.9 39.1 48.0 57.4 126.6 126.6 126.7 126.9 129.2
ppm

HRMS/MS spectrum of compound of 3d

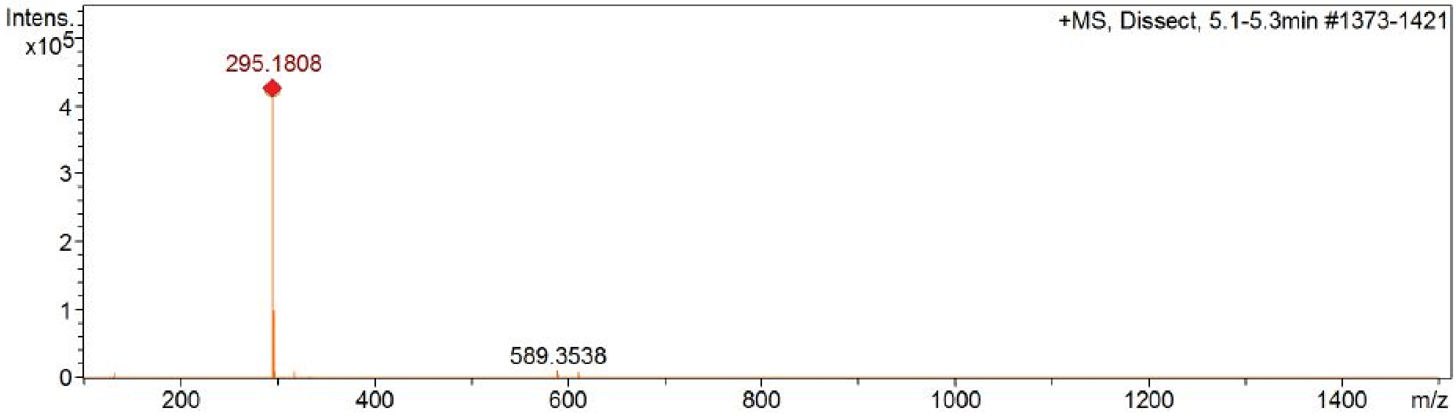

Meas. m/z	#	Ion Formula	Adduct	Sum Formula	Score	mSigma	m/z	err [mDa]	err [ppm]	rdb	e⁻ Conf	N-Rule
295.1808	1	C19H23N2O	M+H	C19H22N2O	100.00	15.3	295.1805	-0.3	-1.0	9.5	even	ok

(*S*)-(4-methylpiperazin-1-yl)(1,2,3,4-tetrahydroisoquinolin-3-yl)methanone (**3e**)

^{1}H NMR (400 MHz, acetone-d6)

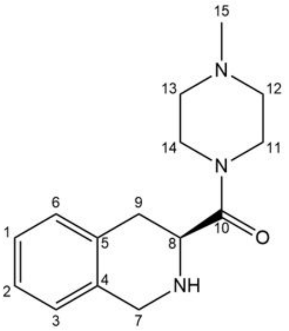

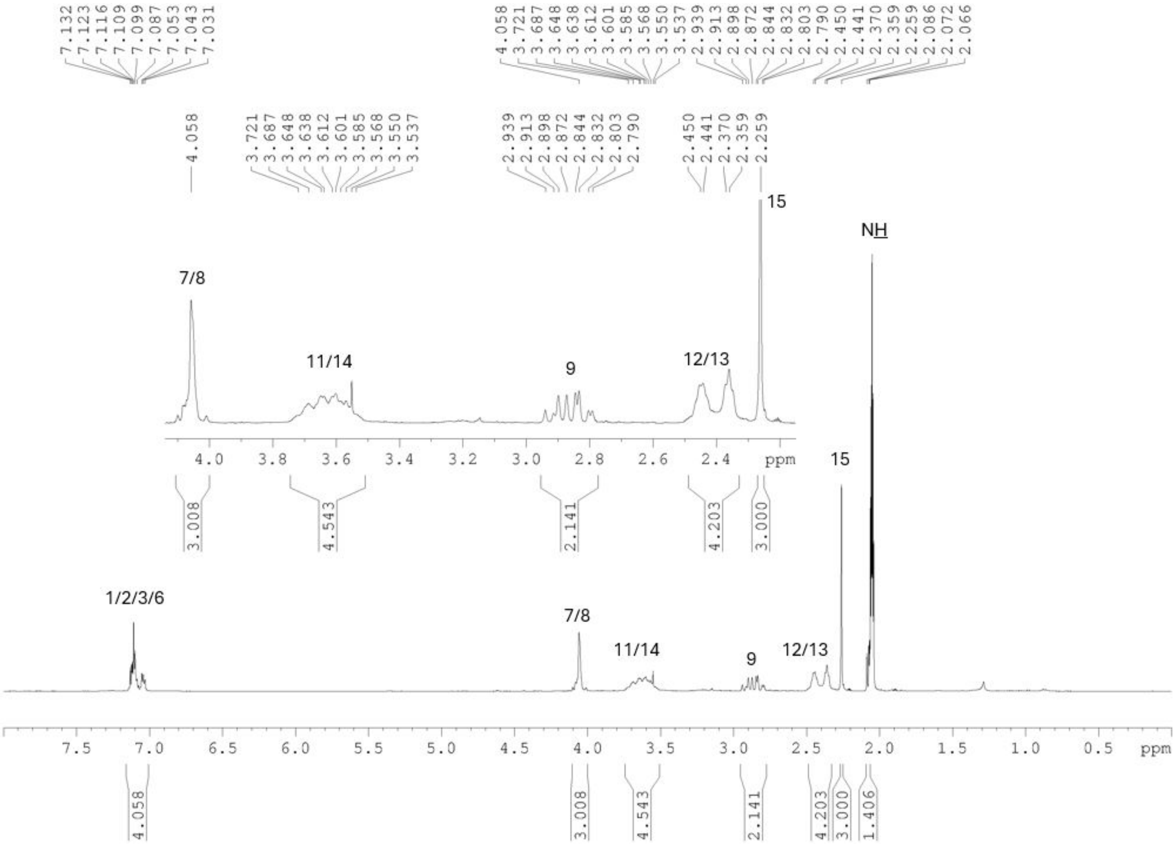

^{13}C NMR/DEPT135 (100 MHz, acetone-d6)

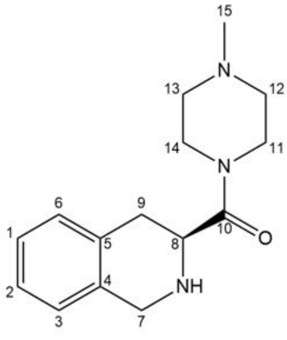

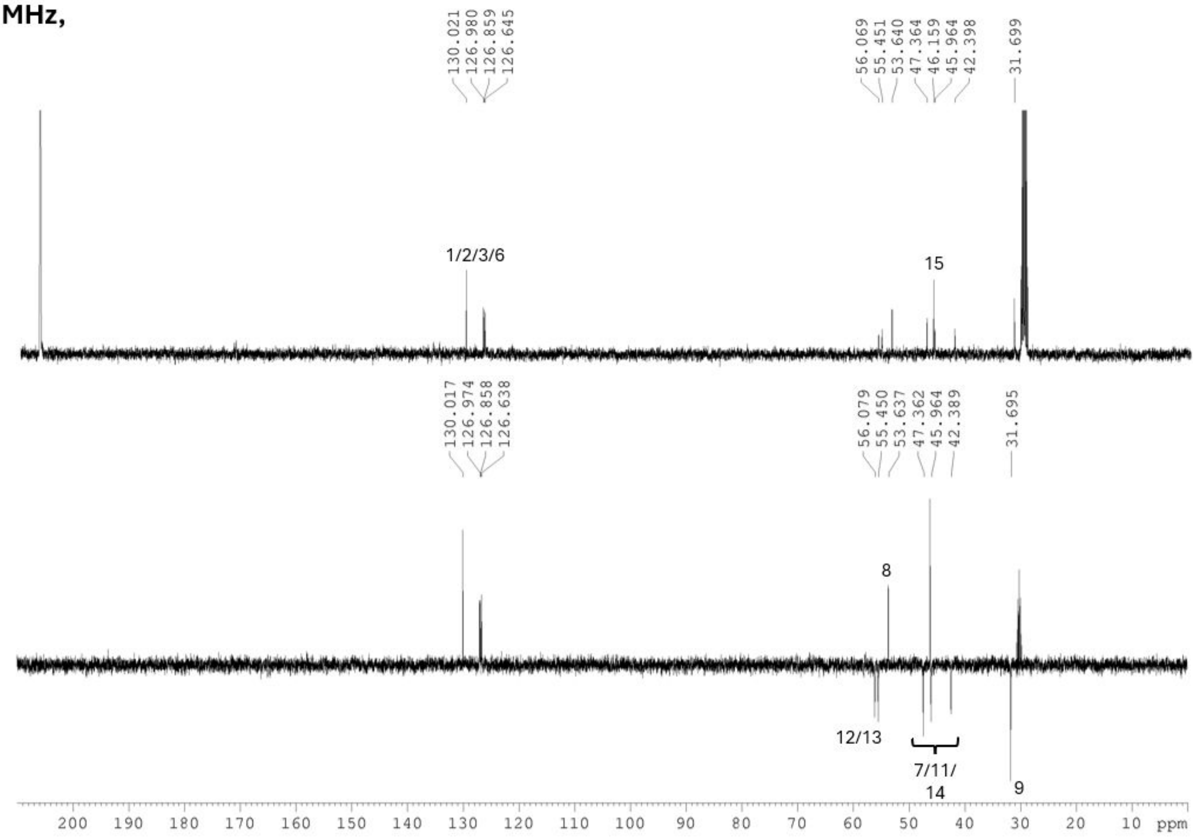

COSY

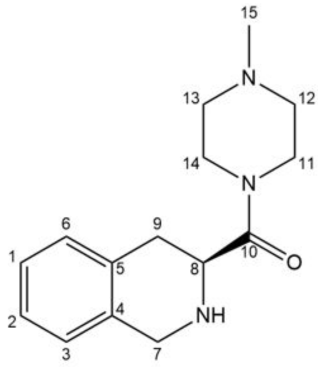
15
13
12
14
11
6
9
1
5
8
10
O
N
N
2
4
NH
3
7

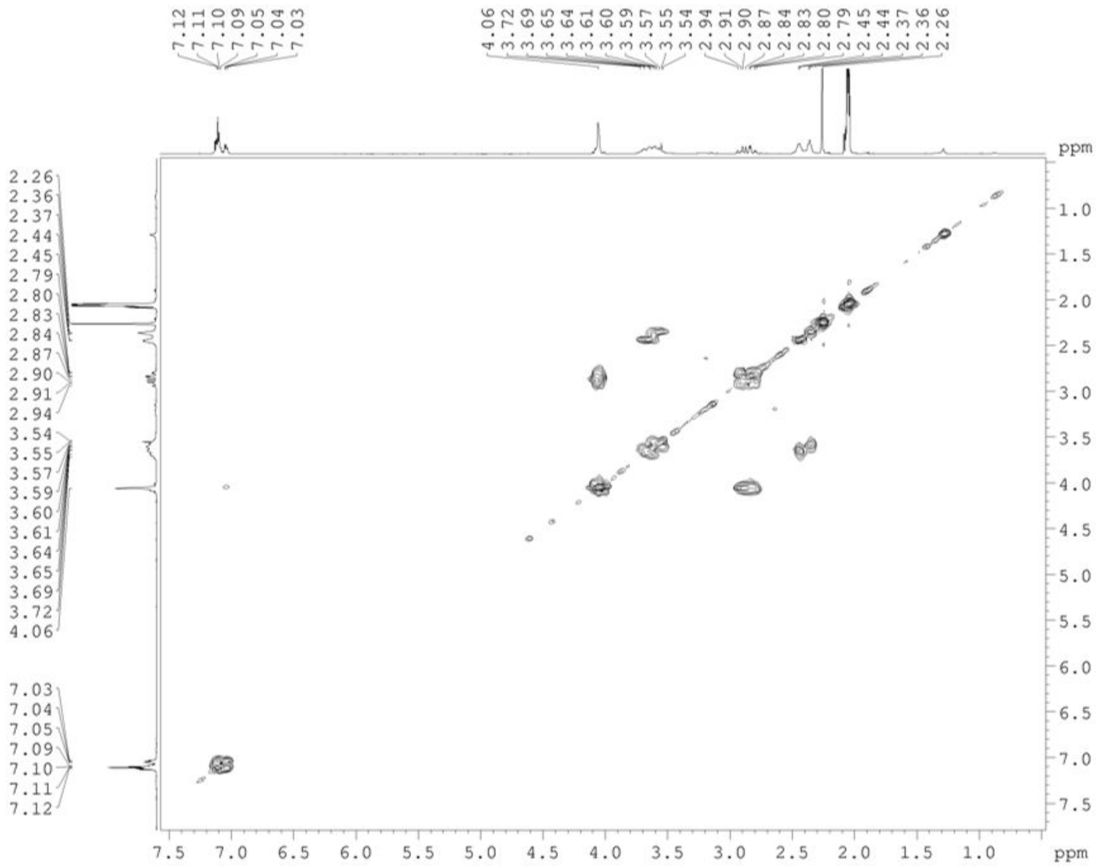
7.12
7.11
7.10
7.09
7.05
7.04
7.03
4.06
3.72
3.69
3.65
3.64
3.61
3.60
3.59
3.57
3.55
3.54
2.94
2.91
2.90
2.87
2.84
2.83
2.80
2.79
2.45
2.44
2.37
2.36
2.26
ppm

HSQC

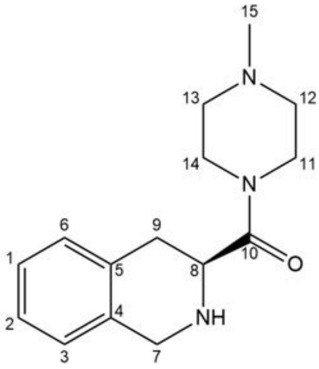
15
13
12
14
11
6
9
1
5
8
10
O
N
N
2
4
NH
3
7

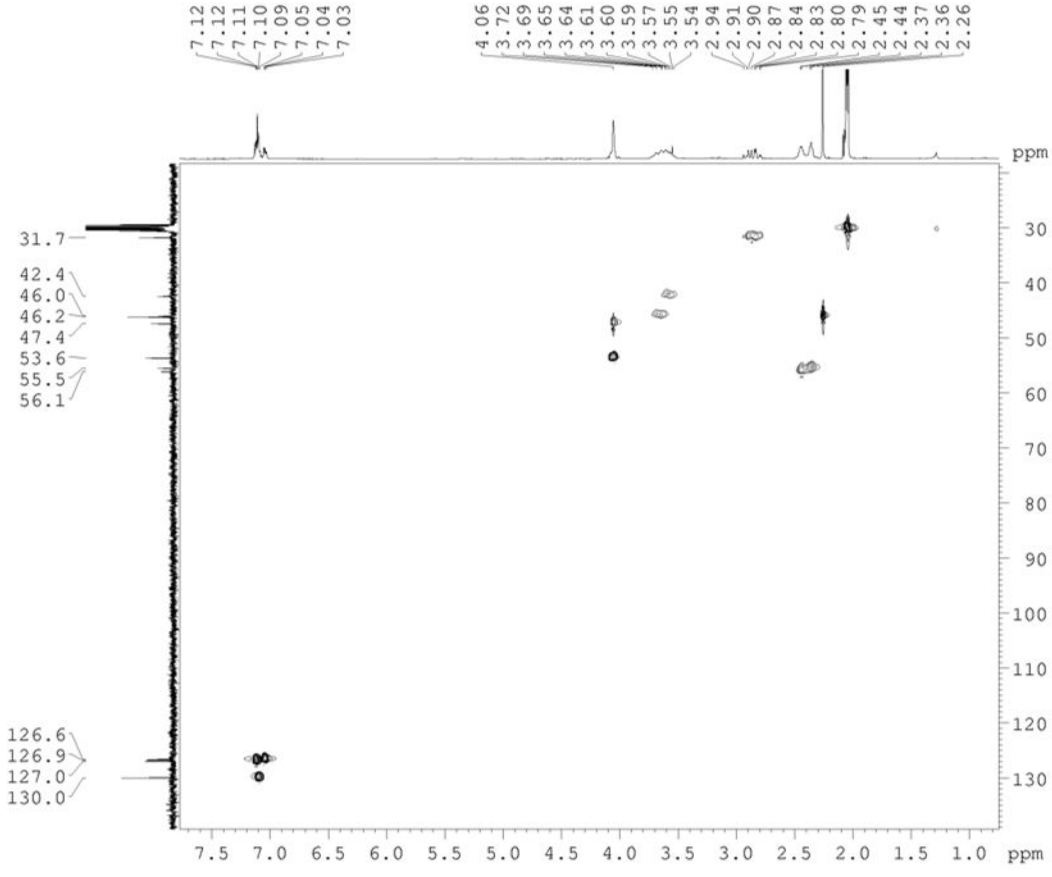
7.12
7.12
7.11
7.10
7.09
7.05
7.04
7.03
4.06
3.72
3.69
3.65
3.64
3.61
3.60
3.59
3.57
3.55
3.54
2.94
2.91
2.90
2.87
2.84
2.83
2.80
2.79
2.45
2.44
2.37
2.36
2.26
31.7
42.4
46.0
46.2
47.4
53.6
55.5
56.1
126.6
126.9
127.0
130.0
ppm

(*S*)-N-(3-morpholinopropyl)-1,2,3,4-tetrahydroisoquinoline-3-carboxamide (**3f**)

^{1}H NMR (400 MHz, acetone-d6)

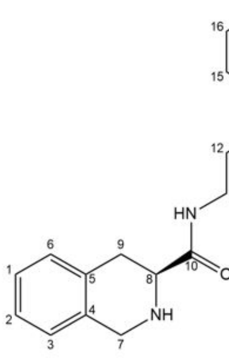

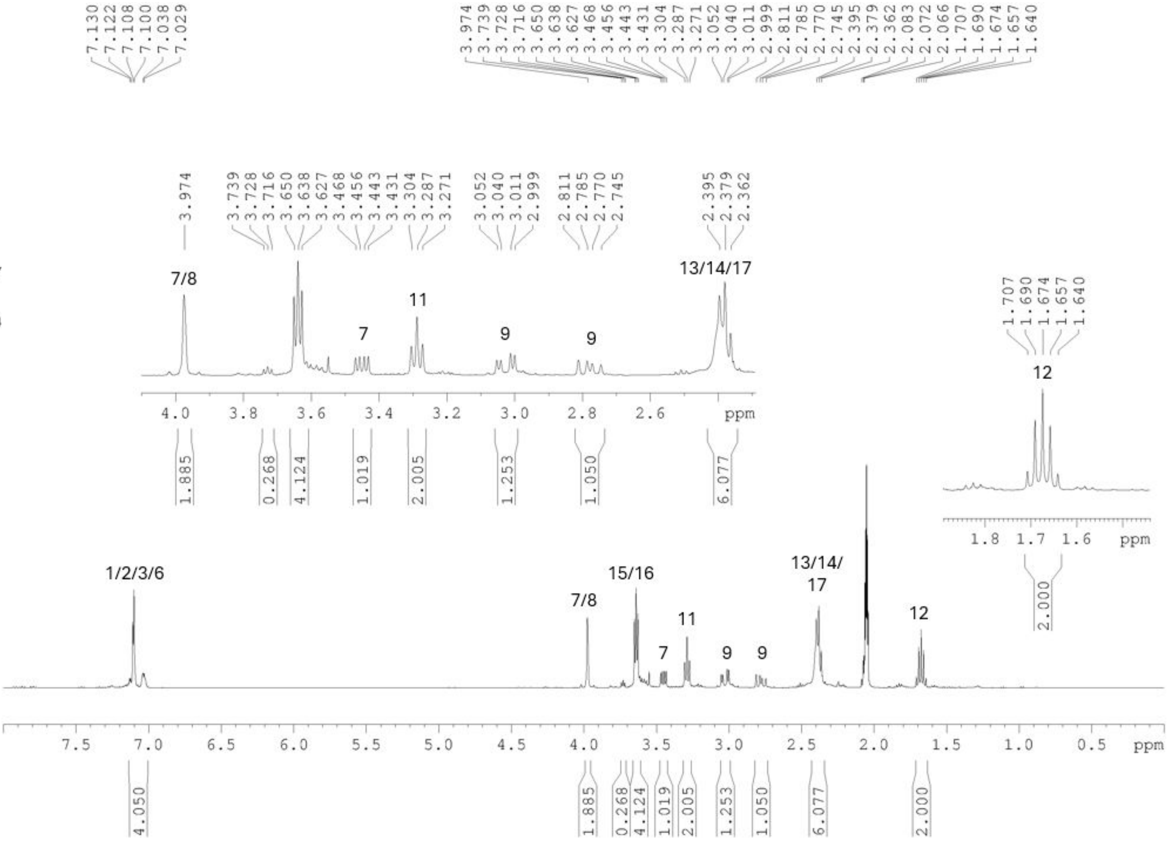

^{13}C NMR/DEPT135 (100 MHz, acetone-d6)

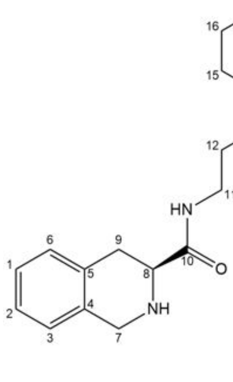

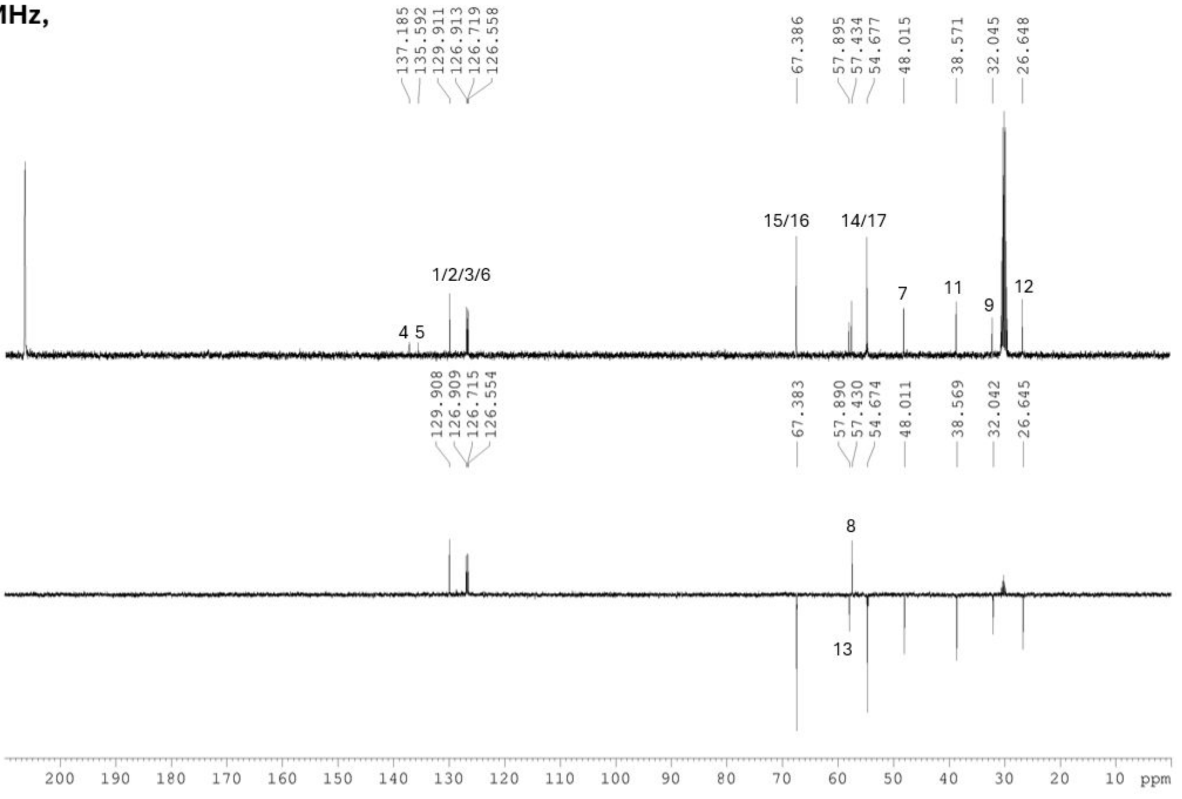

COSY

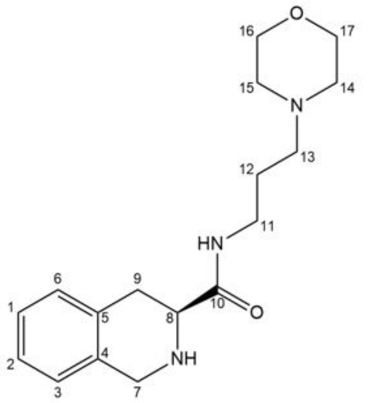
O
N
HN
NH
O
1 2 3 4 5 6 7 8 9 10 11 12 13 14 15 16 17

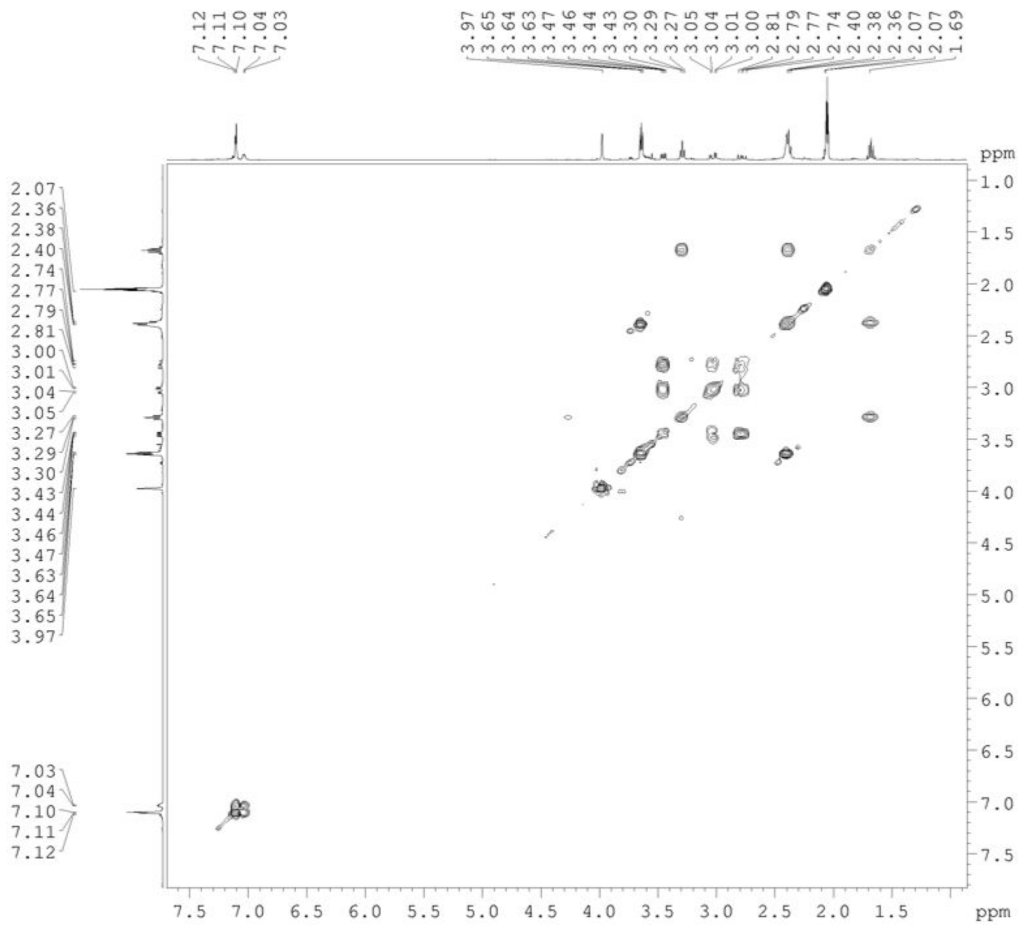
7.12 7.11 7.10 7.04 7.03
3.97 3.65 3.64 3.63 3.47 3.46 3.44 3.43 3.30 3.29 3.27 3.05 3.04 3.01 3.00 2.81 2.79 2.77 2.74 2.40 2.38 2.36 2.07 1.69
2.07 2.36 2.38 2.40 2.74 2.77 2.79 2.81 3.00 3.01 3.04 3.05 3.27 3.29 3.30 3.43 3.44 3.46 3.47 3.63 3.64 3.65 3.97
7.03 7.04 7.10 7.11 7.12
ppm
1.0 1.5 2.0 2.5 3.0 3.5 4.0 4.5 5.0 5.5 6.0 6.5 7.0 7.5
7.5 7.0 6.5 6.0 5.5 5.0 4.5 4.0 3.5 3.0 2.5 2.0 1.5
ppm

HSQC

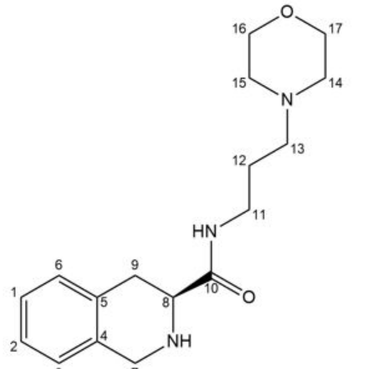
O
N
HN
NH
O
1 2 3 4 5 6 7 8 9 10 11 12 13 14 15 16 17

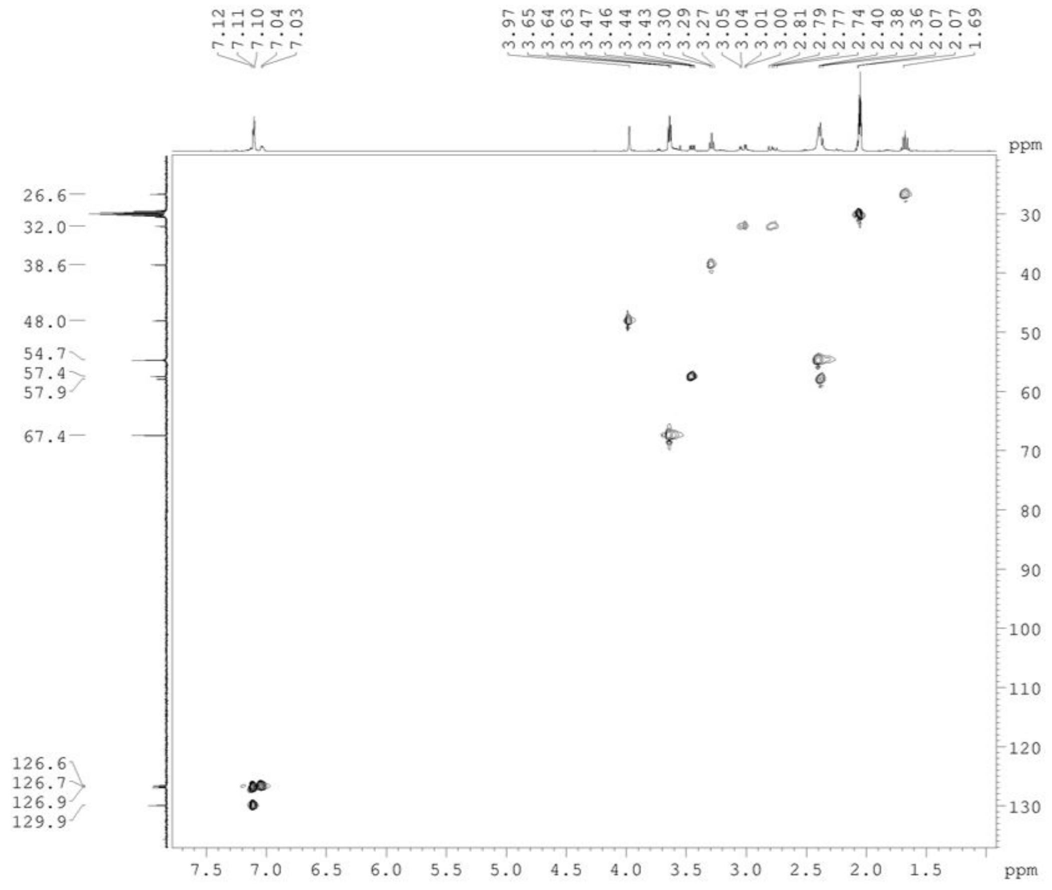
7.12 7.11 7.10 7.04 7.03
3.97 3.65 3.64 3.63 3.47 3.46 3.44 3.43 3.30 3.29 3.27 3.05 3.04 3.01 3.00 2.81 2.79 2.77 2.74 2.40 2.38 2.36 2.07 2.07 1.69
26.6 32.0 38.6 48.0 54.7 57.4 57.9 67.4
126.6 126.7 126.9 129.9
ppm
30 40 50 60 70 80 90 100 110 120 130
7.5 7.0 6.5 6.0 5.5 5.0 4.5 4.0 3.5 3.0 2.5 2.0 1.5
ppm

HRMS/MS spectrum of compound of 3f

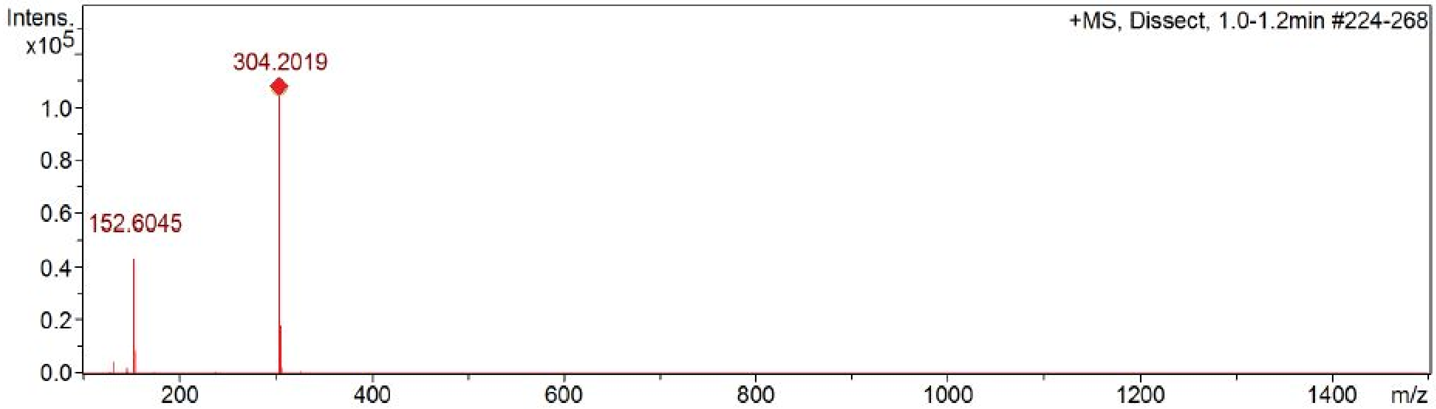

Meas. m/z	#	Ion Formula	Adduct	Sum Formula	Score	mSigma	m/z	err [mDa]	err [ppm]	rdb	e⁻ Conf	N-Rule
304.2019	1	C17H26N3O2	M+H	C17H25N3O2	100.00	14.4	304.2020	0.1	0.2	6.5	even	ok

(*S*)-3-((furan-2-ylmethyl)carbamoyl)-1,2,3,4-tetrahydroisoquinolin-2-ium chloride (**4a**)

^{1}H NMR (600 MHz, DMSO-d6)

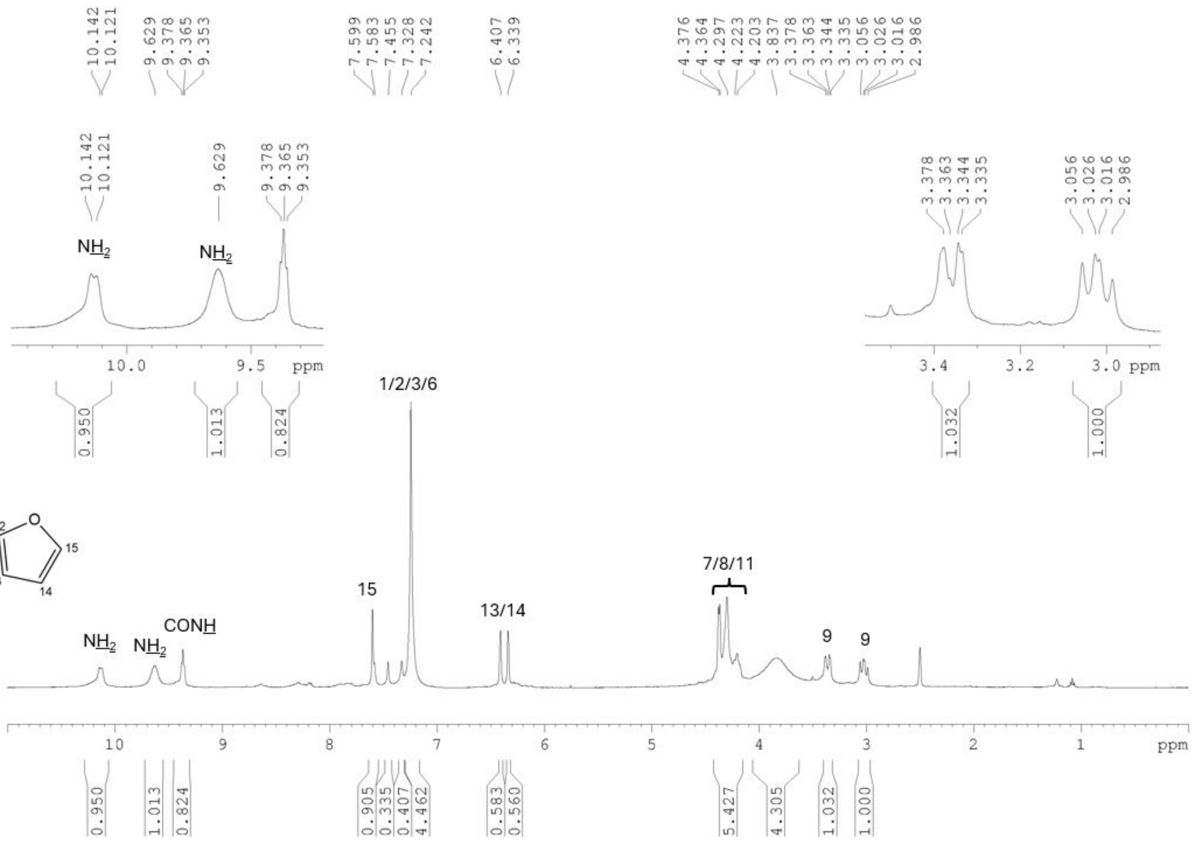

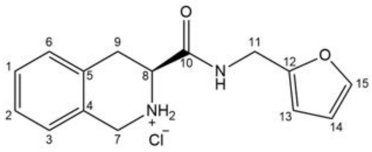

^{13}C NMR/DEPT135 (150 MHz, DMSO-d6)

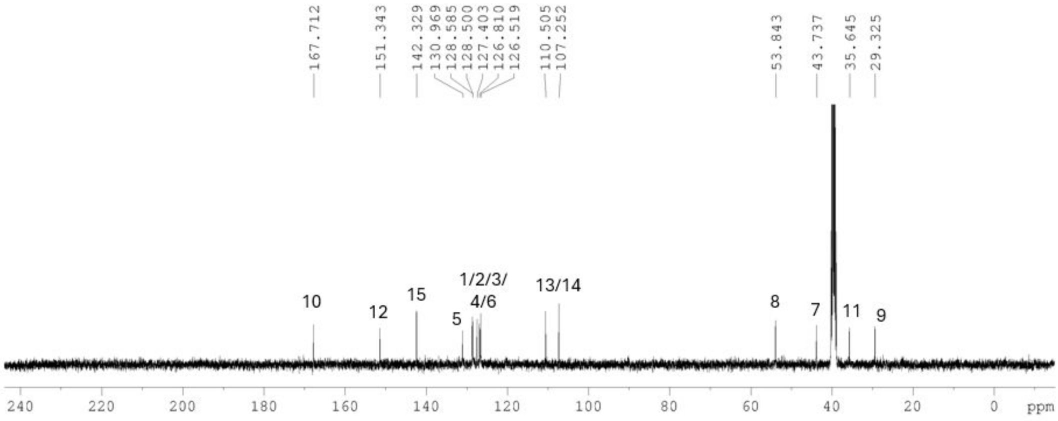

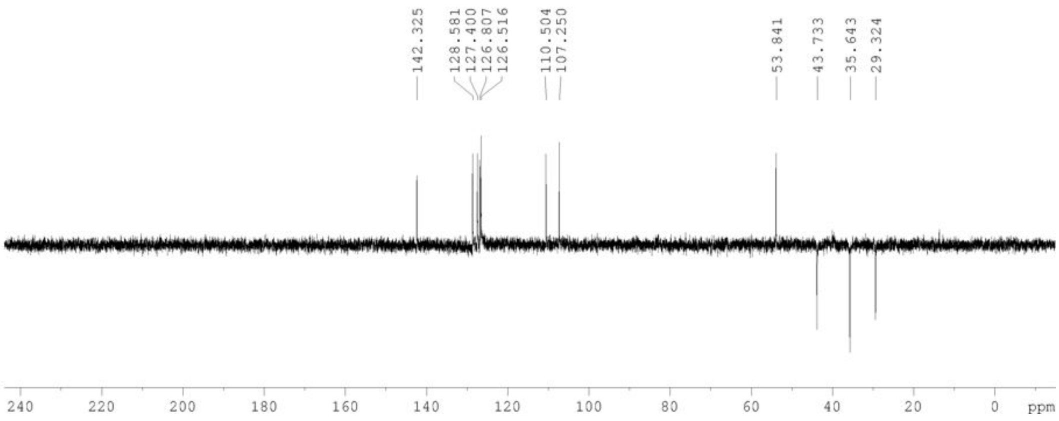

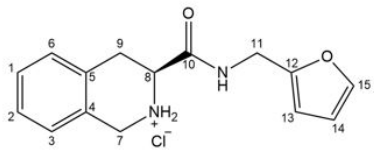

(*R*)-3-((furan-2-ylmethyl)carbamoyl)-1,2,3,4-tetrahydroisoquinolin-2-ium chloride (**4b**)

^{1}H NMR (600 MHz, DMSO-d6)

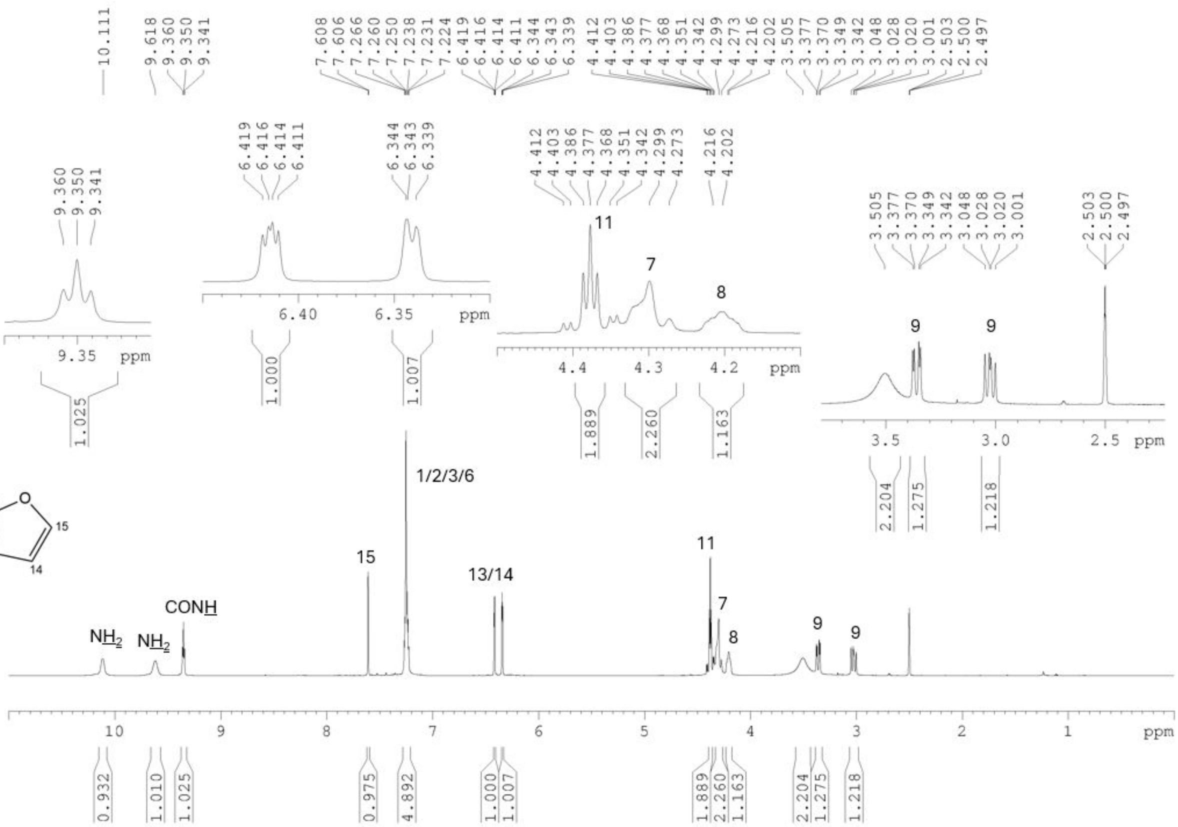

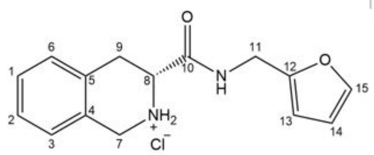

^{13}C NMR/DEPT135 (150 MHz, DMSO-d6)

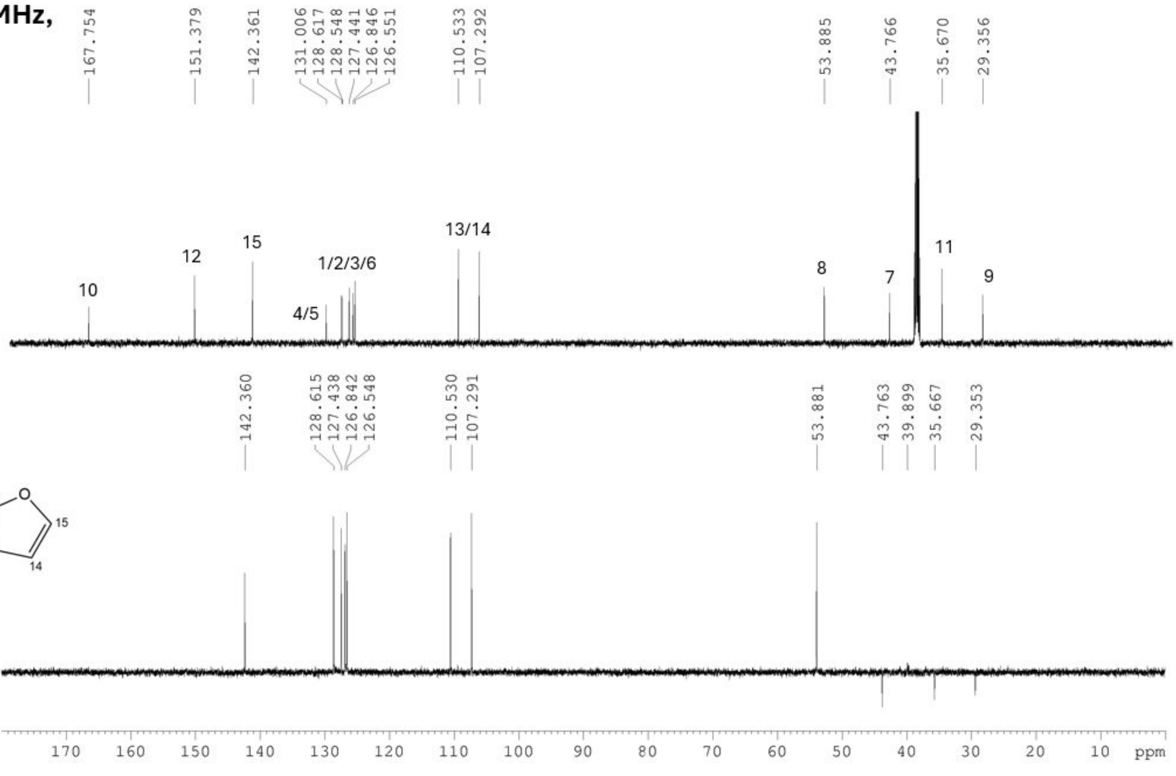

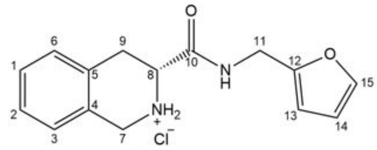

HRMS/MS spectrum of compound of 4b

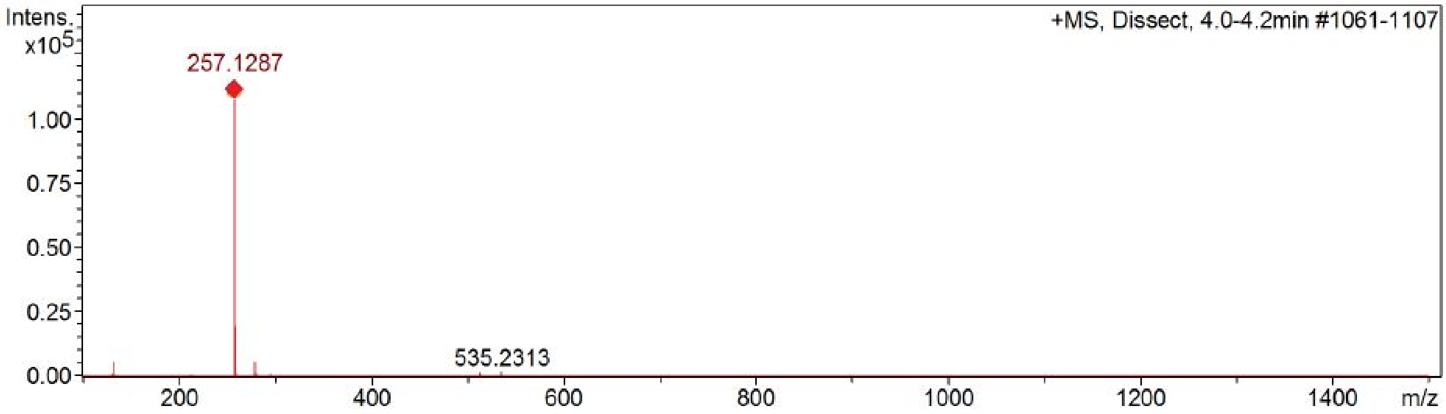

Meas. m/z	#	Ion Formula	Adduct	Sum Formula	Score	mSigma	m/z	err [mDa]	err [ppm]	rdb	e⁻ Conf	N-Rule
257.1287	1	C15H17N2O2	M+H	C15H16N2O2	100.00	6.4	257.1285	-0.3	-1.0	8.5	even	ok

(*S*)-3-(benzylcarbamoyl)-1,2,3,4-tetrahydroisoquinolin-2-ium chloride (**4c**)

^{1}H NMR (600 MHz, DMSO-d6)

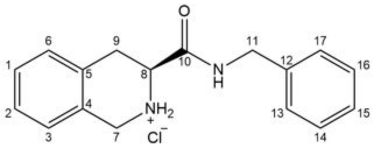

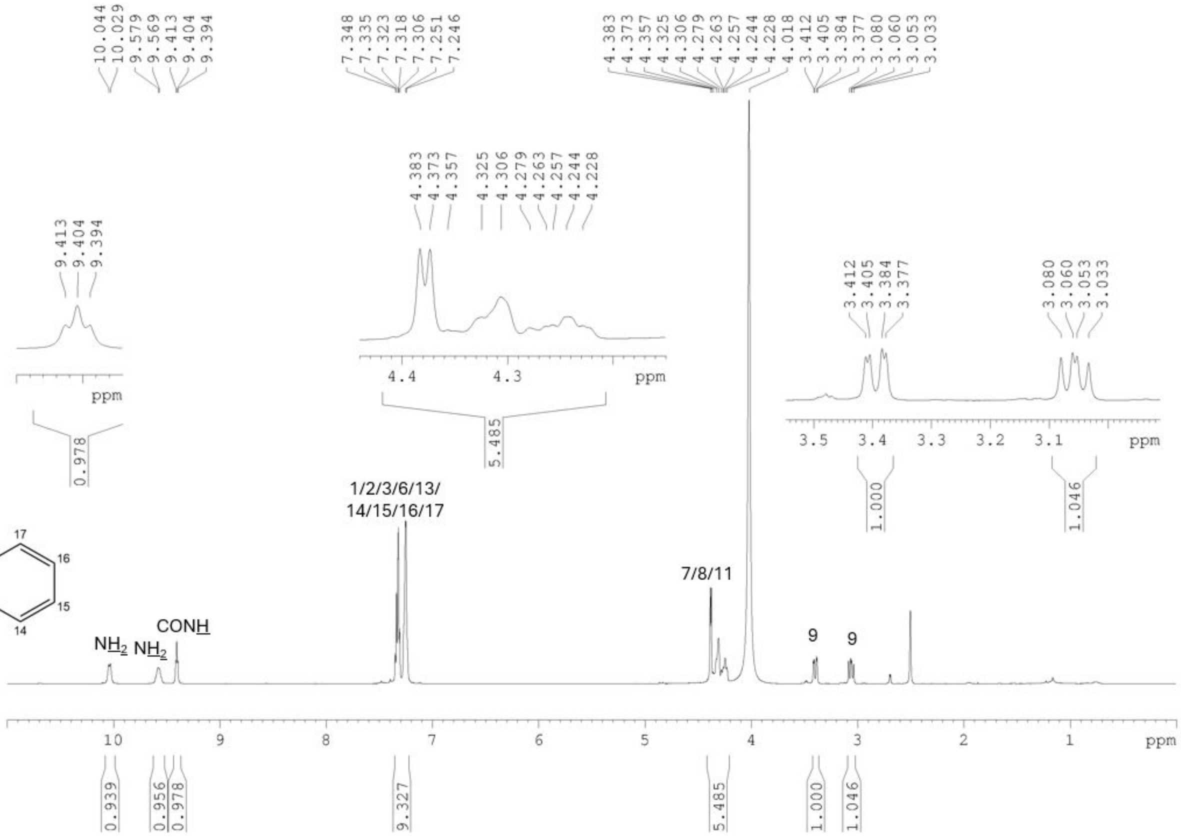

^{13}C NMR/DEPT135 (150 MHz, DMSO-d6)

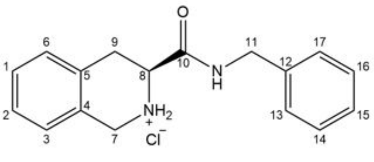

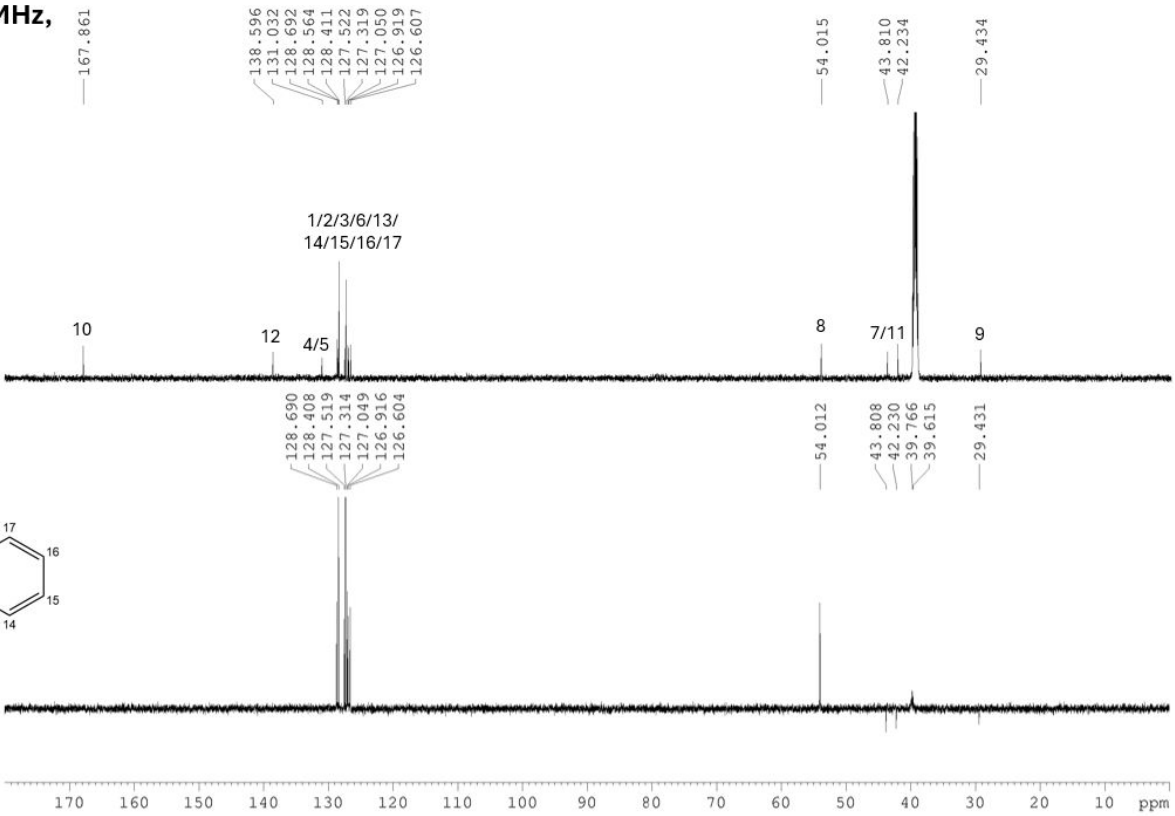

HRMS/MS spectrum of compound of 4c

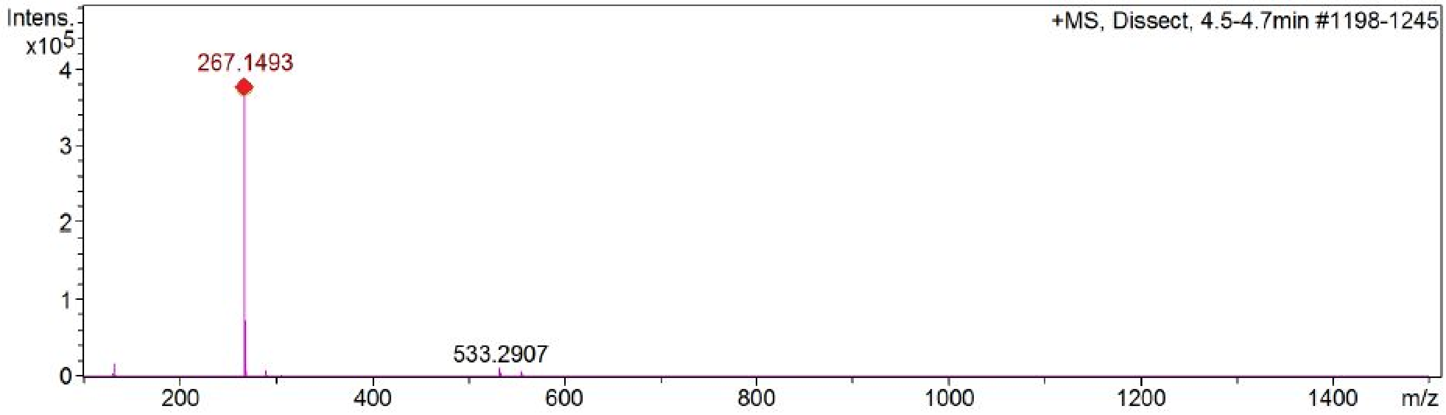

Meas. m/z	#	Ion Formula	Adduct	Sum Formula	Score	mSigma	m/z	err [mDa]	err [ppm]	rdb	e⁻ Conf	N-Rule
267.1493	1	C17H19N2O	M+H	C17H18N2O	100.00	5.7	267.1492	-0.1	-0.4	9.5	even	ok

(*S*)-3-((3-phenylpropyl)carbamoyl)-1,2,3,4-tetrahydroisoquinolin-2-ium chloride (**4d**)

^{1}H NMR (600 MHz, DMSO-d6)

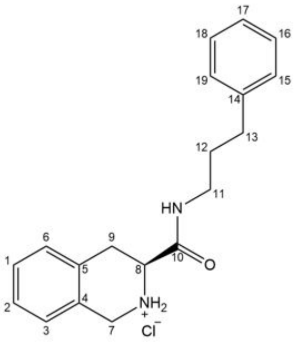

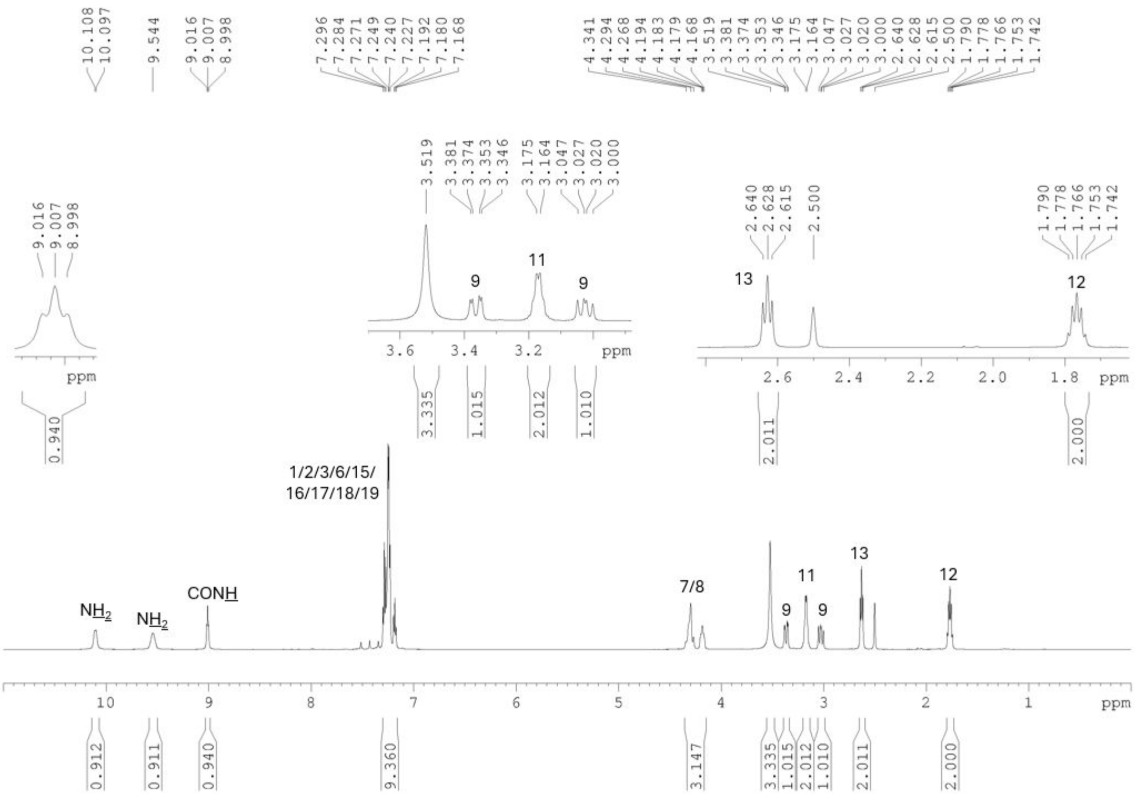

^{13}C NMR/DEPT135 (150 MHz, DMSO-d6)

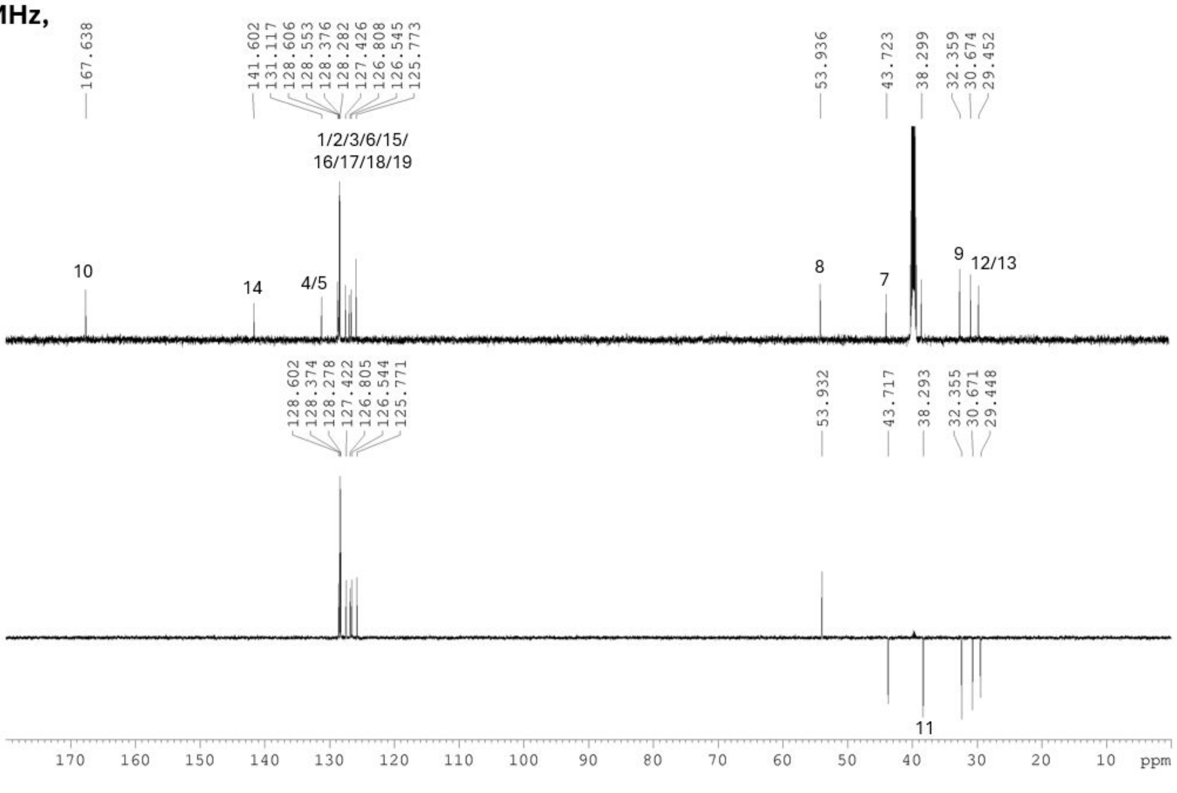

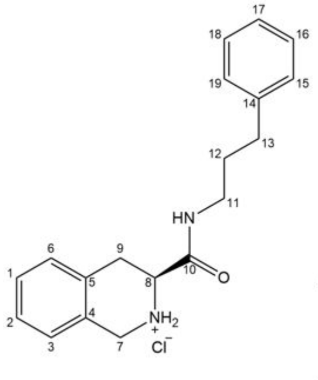

Supplement: Supplementary file 1 [file ao5c11033_si_001.pdf]
